# Physical demands and physiological response of soccer referees in high-level matches: A systematic review

**DOI:** 10.1371/journal.pone.0315403

**Published:** 2025-01-24

**Authors:** Lingling Zhang, Soh Kim Geok, Mohd Rozilee Wazir Norjali Wazir, Liang Qin

**Affiliations:** 1 The National Football Academy, Shandong Sport University, Rizhao, China; 2 Faculty of Educational Studies, Putra Malaysia University, Serdang, Malaysia; 3 China Football Academy, Beijing Sport University, Beijing, China; Tokat Gaziosmanpasa University Tasliciftlik Campus: Tokat Gaziosmanpasa Universitesi, TÜRKIYE

## Abstract

**Background:**

The match physical demands placed on soccer referees are intrinsically connected to their capacity to make accurate judgments, becoming the second most studied theme in associate soccer refereeing.

**Objective:**

This study aims to review the external and internal load performed by soccer referees in high-level competitions, to identify changes in these indicators over different periods as the competition progresses, and to analyze the standards for dividing speed zones and heart rate zones.

**Methods:**

Web of Science, PubMed, Scopus, and EBSCOhost were thoroughly searched. Grey literature sources and Google Scholar were also consulted, with a focus on analysing and comparing the physical demands of soccer referees at different phases of high-level matches.

**Results:**

A total of 14 manuscripts were included in this review. Studies revealed that **the** total distance (TD) covered by referees during a full match ranged from 9 to 12 km. High-intensity running (HIR) constituted 2.0–18.7% of TD, accounting for approximately 38% of total time (TT). Referees reached 80–100% of their maximal heart rate during matches. The standards for dividing speed zones and heart rate zones varied among the selected studies.

**Discussion:**

This systematic review aimed to provide a comprehensive overview of referees’ physical demands (e.g., TD, HIR, and HR) to offer practitioners valuable biological data for training and competition preparation. The lack of uniform criteria for dividing speed and heart rate zones limits data collection, thereby affecting the reporting of distances covered at different exercise intensities.

## Introduction

Soccer players face significant physical and physiological demands during matches [1, 2], a challenge that also extends to referees. To effectively manage match demands, referees must maintain fitness levels that allow them to stay close to the ball, make split-second decisions, and traverse long distances throughout a game [3, 4]. Research has focused on exploring soccer referees’ physical and physiological demands during matches. The physical demands encompass a range of activities from sprints (Sp) or very high-intensity runs (VHI) and high-intensity (HI) runs to moderate (MI) and low-intensity (LI) actions such as jogging (Jg), walking (Wa), and reverse running (RR). The physiological responses of the body are directly influenced by these physical demands. By understanding and comparing the characteristics of both physical and physiological demands during different phases of a match, one can gauge the cumulative physical strain a referee experiences throughout a game. This information is crucial for coaches creating training programs for referees, ensuring that the training categories and loads align with the match demands.

Research has delved into the physical and physiological demands faced by soccer referees during matches, emphasizing the importance of comparing these demands across various time intervals within a game. Weston et al. [5] highlighted the need to assess the overall match intensity before analyzing a referee’s physical performance. Studies by Weston et al. [5], Mascarenhas et al. [6], and Martínez-Torremocha et al. [7] focused on differences in physical requirements between the first and second halves. Conversely, Krustrup and Bangsbo [8], Barbero-Álvarez et al. [2], Fernandes da Silva et al. [9], and Yousefian et al. [10] investigated referees’ physical exertion across different time intervals, including 5-minute, 15-minute, 45-minute segments, and the entire match duration. However, the results from these studies show discrepancies. Referees’ physical performance was found to either decrease or maintain between the later and earlier phases of the matches. Further research is needed to ascertain if diminished physical performance in later match stages is due to fatigue, tactical decisions by referees, or a slower game pace. Discrepancies between studies might stem from varied team strategies and referees’ fitness levels. Additionally, inconsistencies in defining activity categories and heart rate zones across studies might have contributed to the varied findings.

A comprehensive review that systematically evaluates and integrates this research area is lacking, leaving practitioners without a reliable reference for determining training loads. To address this gap, some researchers have undertaken review studies in this domain. Martinho et al. [11] conducted a systematic review covering physical fitness, physiological aspects, nutrition, and age. Although this review collated results and findings from selected studies, it did not summarize data by specific indicators. The study noted that differences in referees’ physical performance relate to game intensity, with referees in top European leagues spending more time in the highest heart rate zone compared to those in the Premier League of Bosnia and Herzegovina [12]– 18% and 10% of match time, respectively. This analysis offers precise match physical demands data, serving as a reference for designing training programs.

Preissler et al. [13] conducted a systematic review of referees’ external loads during matches, focusing on European and South American competitions. While the football development level in these regions is high, exploring other areas can reveal significant gaps, beneficial for less developed regions and organizations like FIFA. This review detailed the classification criteria for speed zones used in the literature and the corresponding data, providing practitioners with detailed and intuitive data references.

While various studies have explored the physical demands and physiological responses experienced by referees, a comprehensive review that systematically evaluates and integrates these studies is lacking. There is a need for a detailed review to accurately understand the challenges football referees face in terms of match physical demands, including the standards for dividing match activity categories and the demands of each category.

Therefore, the primary objective of this review is to elucidate the precise physical requirements and physiological reactions exhibited by football referees during high-level matches. This endeavor aims to consolidate the findings of prior research and provide a comprehensive summary that can serve as a reference for determining the training intensity required for referees at this level.

## Methodology

### Proposal and registration and ethics

**Fig 1:**
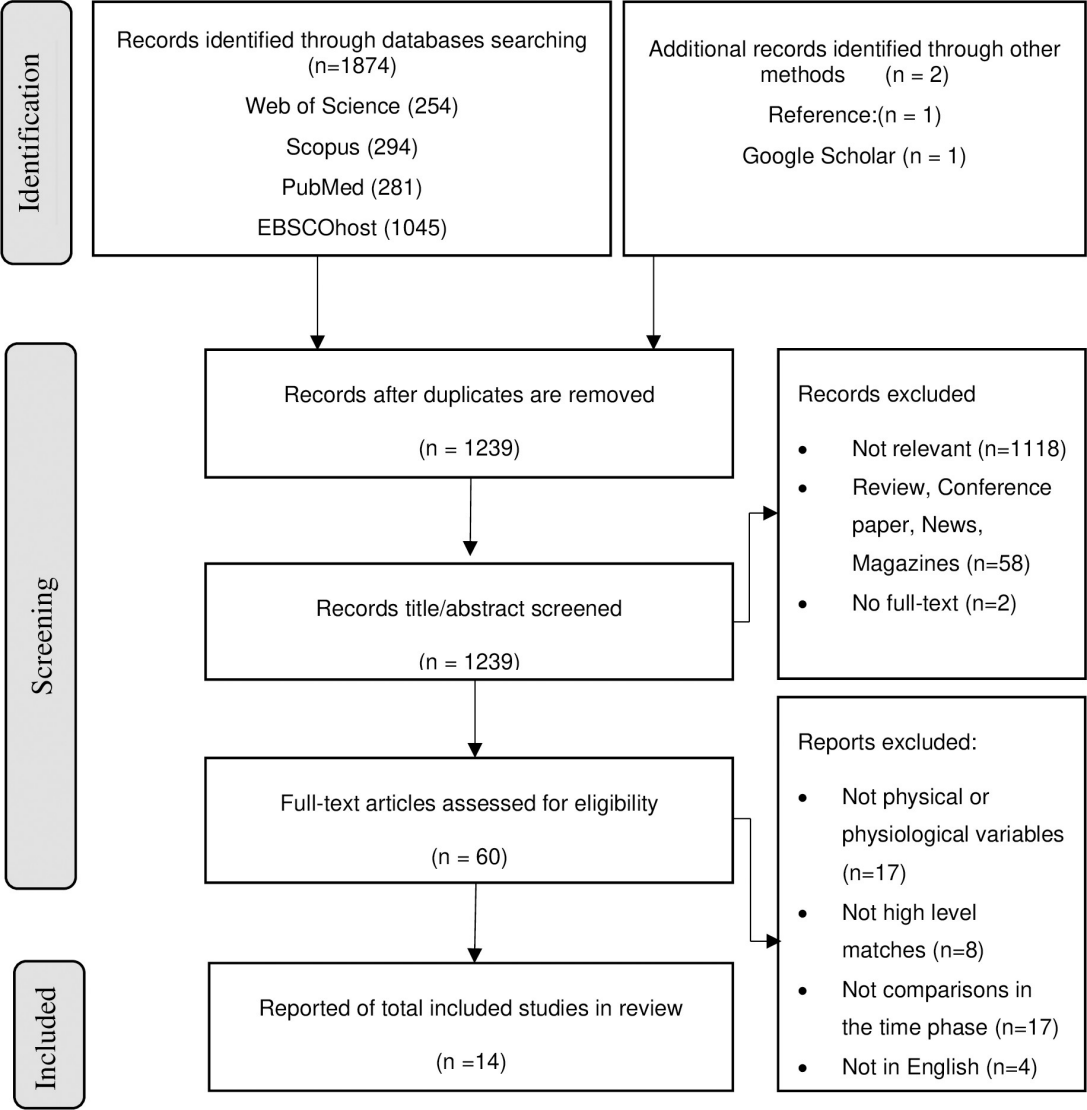
PRISMA summary of the study selection process.

This review adhered to the Preferred Reporting Items for Systematic Reviews and Meta-Analyses (PRISMA) (see Fig 1) guidelines for data selection, collection, and analysis [14]. It was prospectively registered on the International Prospective Register of Systematic Reviews under the registration number INPLASY202220071. The study was conducted following the ethical guidelines of the Institutional Ethics Review Committee of Shandong Sport University.

### Eligibility criteria

The inclusion criteria were based on the PICOS (participation, intervention, comparison, outcome, study design) framework, as outlined in Table 1. The focus was solely on records presenting the physical performance of elite referees in high-level matches. Consequently, studies were included if they: (1) were full-text, peer-reviewed articles published in English that described the physical performance of elite referees (both male and female) in high-level matches; (2) assessed at least one variation of relevant referee match physical performance variables (either internal load or external load); and (3) imposed no restrictions on the sample size, study location, or experimental timeframe of the included studies. Exclusion criteria were applied to studies that: (1) did not involve high-level matches or elite referees; or (2) were published in languages other than English.

**Table 1:** PICO (participation, intervention, comparison, outcome).

Items	Detailed inclusion criteria
Population	Soccer referee
Instrument	High-level match
Comparison	Physical demands and or physiological responses in different match periods (5, 15, 45, and 90 min)
Outcome	Physical performance-total distance, distance covered by high intensity running
	Physiological response-heart rate

### Information sources and search strategy

The literature search was conducted in five international databases: Web of Science, SCOPUS, PubMed, and EBSCOhost, spanning from their inception to June 2023. To ensure the comprehensiveness of the data prior to the publication of this review, the search was repeated in October 2023 across the mentioned databases, and pertinent articles were included. The search strategy utilized key terms such as ’football match’, ’referee’, and ’physical demands’. Searches were performed by title, employing a predefined combination of keywords: "Football", "Soccer", "Referee", "Match Official", "Arbitrator", "Umpire", "physical demand", "fitness demand", "physical load", "physical performance", "physical profile", "match activity", "activity profile", "physical activity", "kinematical activity", "physical exertion", "activity pattern", "movement pattern", "physiological characteristics", "physiological profile", "energy expenditure", "speed", "sprint", "endurance", "yo-yo intermittent", "aerobic", "high intensity". Additionally, reference lists of retrieved papers, authors’ names, and review articles were manually checked for further relevant citations. An example of a PubMed search is as follows ["Football" OR "Soccer" (Title/Abstract)] AND ["Referee" (Title/Abstract) OR "Match Official" (Title/Abstract) OR "Arbitrator" (Title/Abstract) OR "Umpire" (Title/Abstract)] AND ["physical demand" (Title/Abstract) OR "fitness demand" (Title/Abstract) OR "physical load" (Title/Abstract) OR "physical performance" (Title/Abstract) OR "physical profile" (Title/Abstract) OR "match activity" (Title/Abstract) OR "activity profile" (Title/Abstract) OR "physical activity" (Title/Abstract) OR "kinematical activity" (Title/Abstract) OR "physical exertion" (Title/Abstract) OR "activity pattern" (Title/Abstract) OR "movement pattern"(Title/Abstract) OR "physiological characteristics" (Title/Abstract) OR "physiological profile" (Title/Abstract) OR "energy expenditure" (Title/Abstract) OR "speed" (Title/Abstract) OR "sprint" (Title/Abstract) OR "endurance" (Title/Abstract) OR "yo-yo intermittent" (Title/Abstract) OR "aerobic" (Title/Abstract) OR "high intensity" (Title/Abstract)].

### Study selection

The retrieved studies were imported into Mendeley reference management software for duplicate removal. Initially, an experienced librarian assisted in formulating the search strategies. Subsequently, three independent reviewers (Z.L., S.H., and Q.L.) conducted a preliminary screening of titles and abstracts from the identified articles to determine their relevance. Irrelevant materials were excluded from the database before a detailed assessment of the remaining titles and abstracts was carried out according to the pre-established inclusion and exclusion criteria. Articles that satisfied these criteria underwent a qualitative synthesis; this required access to the full text, and any article without available full text was excluded. In instances of disagreement, an additional reviewer (K.G.S.) was consulted until a consensus was reached.

### Data extraction and management

Data extraction was independently carried out by two researchers (Z.L. and S.H.) using a standardized form. The extracted data included the number of games and referees evaluated, the average age and standard deviation of subjects, their gender and nationality, and the level of matches in which the referees were evaluated. Information related to external load variables extracted from the studies comprised: criteria for dividing speed intervals, the total distance covered during the full-time or half match, distance covered in different intensity categories (very high and high), mean heart rate, and maximum heart rate during the match.

### Quality assessment and risk of bias

The Risk of Bias Assessment Tool for Observational Cohort and Cross-Sectional Studies [15] (refer to Table 2) was utilized and includes fourteen items. These parameters relate to the formulation of the research question, the demographic characteristics of the study cohort, the recruitment of groups from a homogeneous population with consistent eligibility criteria, the justification of the sample size, the evaluation of exposure before measuring outcomes, the appropriate duration for observing an effect, the stratification of exposure effects, the quantification of exposures, and the iterative assessment of exposure. Three reviewers (Z.L.L., K.G.S., and M.R.W.N.W.) independently conducted the risk of bias assessments. A thorough evaluation was conducted with each study classified as good, fair, or poor.

**Table 2:** Results of methodological quality assessment for observational cohort and cross-sectional studies.

Study	Quality Assessment Tool for Observational Cohort and Cross-Sectional Studies checklist question number														
	1	2	3	4	5	6	7	8	9	10	11	12	13	14	Quality rating
Asami, Togari & Ohashi (1988)	1	1	1	1	2	3	1	3	1	1	1	3	3	1	**Good**
Krustrup & Bangsbo (2001)	1	1	1	1	2	3	1	3	1	1	1	3	3	1	**Good**
Dottavio & Castagna (2001)	1	1	1	1	2	3	1	3	1	1	1	3	3	1	**Good**
Helsen & Bultynck (2004)	1	1	1	1	2	3	1	3	1	1	1	3	3	1	**Good**
Matthew et al. (2007)	1	1	1	1	2	3	3	3	1	1	1	3	3	1	**Fair**
Mascarenhas et al. (2009)	1	1	1	2	2	3	3	3	1	1	1	3	3	1	**Fair**
Peter Krustrup et al. 2009	1	1	1	1	2	3	1	3	1	1	1	3	3	1	**Good**
Barbero-Álvarez et al. (2012)	1	1	1	1	2	3	3	3	1	1	1	3	3	1	**Fair**
Costa et al. (2013)	1	1	1	1	2	3	3	3	1	1	1	3	3	1	**Fair**
Riiser et al. (2017)	1	1	1	1	2	3	1	3	1	1	1	3	3	1	**Good**
Sánchez et al. (2022)	1	1	1	1	2	3	1	3	1	1	1	3	3	1	**Good**
Martínez-Torremocha et al. (2022)	1	1	1	2	2	3	3	3	1	1	1	3	3	1	**Fair**
Yousefian et al., 2022	1	1	1	1	2	3	1	3	1	1	1	2	3	1	**Good**
Fernandes da Silva et al., 2022	1	1	1	1	2	3	1	3	1	1	1	3	3	1	**Good**

Noted. 1: Was the research question or objective in this paper clearly stated? 2: Was the study population clearly specified and defined? 3: Was the participation rate of eligible persons at least 50%? 4: Were all the subjects selected or recruited from the same or similar populations (including the same time period)? Were inclusion and exclusion criteria for being in the study prespecified and applied uniformly to all participants? 5: Was a sample size justification, power description, or variance and effect estimates provided? 6: For the analyses in this paper, were the exposure(s) of interest measured prior to the outcome(s) being measured? 7: Was the timeframe sufficient so that one could reasonably expect to see an association between exposure and outcome if it existed? 8: For exposures that can vary in amount or level, did the study examine different levels of the exposure as related to the outcome (e.g., categories of exposure, or exposure measured as continuous variable)? 9: Were the exposure measures (independent variables) clearly defined, valid, reliable, and implemented consistently across all study participants? 10: Was the exposure(s) assessed more than once over time? 11: Were the outcome measures (dependent variables) clearly defined, valid, reliable, and implemented consistently across all study participants? 12: Were the outcome assessors blinded to the exposure status of participants? 13: Was loss to follow-up after baseline 20% or less? 14: Were key potential confounding variables measured and adjusted statistically for their impact on the relationship between exposure(s) and outcome(s)?

## Results

### Study selection

Initially, there were 1,874 hits from Web of Science, Scopus, PubMed, and EBSCOhost, along with two hits from a reference search and Google Scholar. After removing all duplicates and screening titles and abstracts, 60 articles remained. Upon full-text review, 14 pieces of literature were selected for this study (see Fig 1). Ten studies were evaluated as high quality, while the other four were considered moderate level (refer to Table 2).

### Participants and matches

The participants in the selected studies were predominantly male, with only one study focusing on female referees. The average age of the referees ranged from 29 to 42 years. They were elite field referees, not assistants, officiating at national and international levels in professional leagues. They were classified as Category 1 in three studies [16–18], FIFA in six studies [1, 2, 6, 8, 9, 19] and national level in five studies [6–8, 10, 20] or professional (n = 1) [5] referees. Specifically, category 1 refers to match officials who officiate the national top league. The analyzed matches are international (official not friendly) or top national competitions.

### Physical demands of soccer referees

The demands placed on a football referee during a game are elucidated through match physical analysis and physiological measurements taken during match play [19]. Physical analyses primarily derive from the Total Distance (TD) covered, Very High-Intensity (VHI), High-Intensity Running (HIR), and Heart Rate (HR) as observed in the selected studies of the current review (Table 3). The specific results are presented in the following sections. The standards of dividing match activity categories and the distance performed of each category in the selected studies are reported (refer to Table 4).

**Table 3:** Overview of publication details.

Publication	Participants		Match Level	Comparison of Match periods	Outcomes				
	Number	Gender			Very high-intensity	High-intensity	Total distance (m)	HRmean (Beats min^-1^)	HRmax%
Asami, Togari & Ohashi (1988)	13	male	FL+IM	15 min	NR	17.7%TD →	9990↑	NR	NR
				45 min					
Krustrup & Bangsbo (2001)	27	male	CM	5 min	1.7%TD ↓	4.1%TD ↓	10070 ↓	162	85%
				15 min					
				45 min					
Dottavio & Castagna (2001)	18	male	FL	15 min	5.3%TD →	13.7%TD ↑	11367 ↓	163±5	89%
Helsen & Bultynck (2004)	17	male	IM	15 min	R:33%TT	R:37%TT	NR	155±16	85%
Weston et al. (2007)	19	male	FL	45 min	NR	6.8%TD →	11620 ↓	NR	NR
Mascarenhas et al. (2009)	5	male	NM	45 min	2.0%TD	NR	10323 ↓	163±8↓	84%
Peter Krustrup et al. 2009	15	male	IM	15 min	R:2.1%TD →	R:18.7%TD →	R:10270 →	150±3↓	NR
Barbero-Álvarez et al. (2012)	7	male	IM	5 min	R:6.7%TD ↓	R:18.7% TD ↓	R:10197 ↓	NR	NR
				15 min					
				45 min					
Costa et al. (2013)	11	male	PM	45 min	56.1%TT ↓	37.8%TT ↑	10449 →	165	89%
Riiser et al. (2017)	16	male	FL+SL	5 min	R:0.1%TD	R:2.0%TD →	R:11218 →	NR	NR
				45 min					
Sánchez et al. (2022)	17	female	NR	45min	0.6%TD	3.1%	9945 →	NR	NR
Martínez-Torremocha et al. (2022)	19	Male	FL	45min	2.0%TD	NR	10417→	147±12→	NR
Yousefian et al., 2022	23	Male	CM	5 min	0.2%↓	26.7%↑	9353↓	155±6↓	85%↓
				45 min					
Fernandes da Silva et al., 2022	40	Male	FL	15 min	0.7%	10.2%↓	10818↑	NR	NR
				45 min					

**Noted.** *FL = First League, SL = Second League, IM = International Match, NM = National Match CM = Competitive Match PM = Professional Match *TD = Total Distance, TT = Total Time.NR = Not Reported *The arrows are used to indicate the trend of this variable towards the end of the game, “↑” = increase, “↓” = decrease, “→” = no significant change

**Table 4:** Velocity bandings of the selected articles.

Publications	Maximal effort (Sprint)	Very High-intensity	High-intensity	Moderate-intensity
Asami, Togari & Ohashi (1988)	NR	NR	NR	NR
Dottavio & Castagna (2001)	>24.1 km·h^-1^	NR	18.1–24 km·h^-1^	13.1–18 km·h^-1^
Krustrup & Bangsbo (2001)	25 km·h^-1^	NR	18 km·h^-1^	15 km·h^-1^
Helsen & Bultynck (2004)	>24.1 km·h^-1^	NR	18.1–24 km·h^-1^	13.1–18 km·h^-1^
Matthew et al. (2007)	NR	NR	>19.8 km·h^-1^	>12 km·h^-1^
Mascarenhas et al. (2009)	NR	NR	NR	>12 km·h^-1^
Peter Krustrup et al. (2009)	NR	NR	NR	NR
Barbero-Álvarez et al. (2012)	>18 km·h^-1^	NR	13.1–18 km·h^-1^	8.1–13 km·h^-1^
Riiser et al. (2017)	>25.2 km·h^-1^	NR	>19.8 km·h^-1^	14.4–19.8 km·h^-1^
Costa et al. (2013)	NR	NR	NR	NR
Sánchez et al. (2022)	>24 km·h^-1^	21–24 km·h^-1^	18–21 km·h^-1^	12–18 km·h^-1^
Martínez-Torremocha et al. (2022)	>24 km·h^-1^	21–24 km·h^-1^	18–21 km·h^-1^	NR
Yousefian et al. 2022	>25.2 km·h^-1^	>18 km·h^-1^	>13 km·h^-1^	NR
Fernandes da Silva et al. 2022	>23.1 km·h^-1^	NR	18.1–23 km·h^-1^	15.1–18 km·h^-1^

Noted. NR: Not Reported

#### Physical demands of soccer referees in games—Total Distance (TD)

Table 3 showcases the Total Distance (TD) covered by referees during both halves and the entirety of the matches across all 14 articles. Referees traversed between 9.4 to 11.6 km over the course of a full match [2, 5, 6, 8, 18]. Six studies observed that referees covered shorter distances in the latter stages of matches [2, 5, 8, 21]. Conversely, five studies reported no significant variance in the total distance covered by referees between the first and second halves [7, 8, 17, 20, 21].

Additionally, eight studies provided a detailed examination of distances covered within shorter intervals, such as 5 or 15-minute periods. Ottavio and Castagna [16] highlighted a significant decrease in distance covered by players in the last 15 minutes of each half. Similarly, Peter Krustrup and colleagues [19] observed a decline in the distance covered by referees during specific intervals. Notably, over 15-minute segments, referees were found to cover 9%, 11%, and 10% less distance during the periods of 30–45, 60–75, and 75–90 minutes, respectively. Assistant referees exhibited even larger declines, with reductions of 37% and 42% in the intervals of 60–75 and 75–90 minutes, respectively, compared to the first 15-minute period.

#### Physical demands of soccer referees in games—High-Intensity Running (HIR)

The study of physical exertion has positioned high-intensity running (HIR) as a focal point. Among the fourteen studies reviewed, HIR was quantified as a percentage of the total distance (TD) covered during matches in twelve studies, while the remaining two assessed HIR based on the total time spent. HIR constituted between 2.0% and 18.7% of TD and approximately 38% of total time on average.

To analyse distances covered at different speeds, speed zones were established in the studies. However, when summarizing distances covered by HIR, it was observed that the criteria for dividing speed zones varied among studies. For instance, Krustrup and Bangsbo [8] defined speeds greater than 18 km/h as high intensity, while Yousefian et al. [10] categorized 13–17.9 km/h as the high-intensity zone. For the purposes of this study, following the guidelines of Ottavio and Castagna [16], distances covered at speeds greater than 18 km/h and >19.8 km/h were aggregated. The results displayed a range of 520–2159 meters, with an average distance covered of 976 meters.

In their research, Krustrup et al. [19] discovered that FIFA referees consistently executed high intensity running (HIR) during both halves of international matches. However, there was a notable decrease of 27% and 28% observed in the 30–45 minute and 60–75-minute intervals, respectively, when compared to the initial 15-minute period. This pattern of diminished HIR as matches progressed was also reported by Barbero-Álvarez et al. [2] and Krustrup et al. [19], with the latter study indicating a reduction of 25% from the start to the end of the matches. Ottavio and Castagna [16] noted a decrease in HIR during the second half, particularly in the first and last 15-minute intervals, attributing this decline to reduced vigorous physical activity. The investigations by Weston et al. [5] and Barbero-Álvarez et al. [2] did not detect any statistically significant changes in HIR at the 15 and 45-minute marks. In a unique study that examined variations between consecutive matches, a significant decline in HIR was noted among referees in the English Premier League during their second match.

#### Physical match demands of soccer referees in games—Heart Rate (HR)

The measurement of heart rate is pivotal in assessing the physiological demands of physical activities, providing a reliable indicator of internal load [18]. This metric has been instrumental in evaluating the physical exertion faced by referees during football matches [1]. Heart rate data offer insights into referees’ ability to manage their exertion levels throughout a match, correlating with various intensities of physical activity [1, 10].

A study by Catterall et al. [22] on referees in the English top division found peak heart rates of 200 beats per minute, with an average heart rate of 165 beats per minute, equating to approximately 95% of their maximum heart rate (HRmax). Similarly, Ottavio and Castagna reported that referees in Italy’s Serie A and B leagues had an average heart rate of 163 ± 5 beats per minute, around 89.1% of their HRmax. Supporting these findings, Helsen and Bultynck [1] observed an average heart rate of 155 beats per minute.

Further research by Helsen and Bultynck [1] and Krustrup and Bangsbo [8] revealed that referees frequently reach 95% to 100% of their HRmax during matches. Studies [7, 8, 16, 20, 23] have shown heart rate patterns of international referees to be similar, albeit slightly lower, to those of national league referees, although the differences were not statistically significant. Costa et al. [17] found that Brazilian professional league referees spent about 56.1% of match time within the 90–100% HRmax zone, with an impressive 95% of total match time maintaining a heart rate level above 80% of their HRmax.

## Discussion

The present systematic review provided a comprehensive overview of referees’ physical demands (e.g., TD, HIR, and HR) to provide practitioners with valuable biological data for training and competition preparation.

### Physical demands of soccer referees in games—Total Distance

TD covered is a crucial metric for evaluating the physical demands on referees during matches. According to selected studies, TD ranged from 9.4 km to 11.6 km [5], aligning with distances covered in South American tournaments (9319.61m) and European competitions (11,187.02m) as reported by Preissler et al. [13]. Comparable findings were observed in Portugal, where male referees covered a TD of 9353 meters in competitive matches, and in Brazil’s Series A and B matches, where distances of 10818 meters and 10604 meters were reported for male referees, respectively [9]. The increase in TDs over the past three decades and their recent stability suggest an evolution in football team tactics. The only study on female referees documented an average TD of 9945 meters [19], surpassing the TD covered by Portuguese male referees in the same period [21], highlighting the development of women’s football and suggesting potential changes in men’s football strategies and tactics.

Fatigue can be identified by comparing distances travelled during different periods of the match [22]. This study not only focused on the TD during the entire match or its different phases but also on changes in TD covered towards the match’s end. Five articles indicated that referees travelled shorter distances as games progressed. Ottavio and Castagna [16] observed a significant reduction in TDs covered in the last 15 minutes of each half. Krustrup and Bangsbo [8] noted that referees covered 9%, 11%, and 10% less TD from 30 to 45 minutes, 60 to 75 minutes, and 75 to 90 minutes, respectively, compared to the first 15-minute periods. This decline towards the game’s end suggests fatigue among referees and/or players, leading to a slower game pace. Conversely, Barbero-Álvarez et al. [2] found no differences between 15-minute intervals or halves. The variations in findings could be attributed to the specific tempo of the game [6], referees’ fitness levels [2], experience, the competitive level of the matches [9], and the competition’s status [2]. A comprehensive understanding of referees’ physical requirements in competitions and relationship between their physical level and actual performance necessitates controlling or studying these factors simultaneously.

### Physical demands of soccer referees in games—High-Intensity Running (HIR)

HIR distances covered by referees during matches vary significantly, ranging from 2.1% [22] to 25.4% [2] of the Total Distance (TD). The thresholds for speed zones vary across studies, with some setting it at 13.1 km/h and others at 19.8 km/h [5, 18], indicating differences in criteria for HIR. Despite these variations, HIR is recognized as a vital metric reflecting the physical demands placed on referees [16].

Evidence points to a decrease in HIR distances as matches progress, indicative of fatigue. This decline, particularly noted in the later stages of each half, was observed by Krustrup et al. [19]. Additionally, the HIR patterns of referees closely match those of players, suggesting that match dynamics similarly impact both groups [5]. In elite competitions, referees are required to cover more significant HIR distances compared to those in lower-level matches, highlighting the challenges of maintaining high-intensity running ability, especially in the latter stages of the game. This necessitates targeted training to enhance referees’ capacity for high-intensity running.

The findings indicate HIR distances during matches range from 520 to 2,159 meters, with an average of 976 meters. This mean value aligns closely with the 800 meters reported by Preissler et al. [13]. These results, including the average and maximum requirements identified in this review, provide valuable benchmarks for coaches to consider when designing training regimens and setting appropriate training loads for referees.

### Physiological response of soccer referees in games—Heart Rate

Heart rate is a reliable indicator of cardiovascular stress and effectively reflects the physical burden experienced during physical activity. Studies consistently show that referees’ mean heart rates exceed 80% of their maximum heart rate (HRmax) during matches. Catterall et al. [22] observed maximal heart rates of 200 beats per minute in top-flight English matches, while Krustrup & Bangsbo [8] reported that referees often reached 95–100% of their HRmax. This, combined with the correlations between distance traversed and %HRmax, underscores the high physical demands placed on referees. Notably, heart rates increase towards the end of each half, indicating intense play, likely due to teams’ final efforts. This highlights the necessity for referees to maintain stamina for at least 45 minutes of continuous strenuous activity.

However, the methodology for determining HRmax can influence outcomes. Peak rates measured during games, laboratory-based incremental exercise tests, and the "220 minus age" formula are three common methods. Helsen and Bultynck [1] noted that in-game HRmax values often exceed those derived from laboratory tests. Moreover, the generic "220 minus age" method can significantly deviate, with variations ranging from -11 to +18 beats per minute. This discrepancy underscores the importance of exercising caution when comparing studies that employ different methodologies for determining HRmax.

## Conclusions

The physical demands on referees in high-level matches are substantial, with male and female referees covering an average of 10.5km and 9.9km respectively. Within this total distance, approximately 1000m is attributed to high-intensity running. From a physiological standpoint, peak heart rates reach 80–100% of HRmax. Notably, both the total distance and high-intensity runs covered by referees tend to decrease in the second half of the match or do not change significantly, a trend that may be due to fatigue or a general reduction in game speed resulting from player fatigue. Football associations and federations can utilize the findings of this study as a benchmark for organizing referee training programs or designing fitness tests. Furthermore, the lack of uniform criteria for dividing speed and heart rate zones among the reviewed studies restricts data collection, thus impacting the reporting of distances covered at varying exercise intensities.

## Supporting information

S1 Table Detailed search strategy. (PDF)

S1 File Studies identification. (PDF)

## References

[1] Helsen W, Bultynck JB. Physical and perceptual-cognitive demands of top-class refereeing in association football. Journal of Sports Sciences. 2004;22(2):179–89. doi: 10.1080/02640410310001641502 WOS:000187456600004. 14998096

[2] Barbero-Álvarez J, Boullosa DA, Nakamura FY, Andrín G, Castagna C. Physical and physiological demands of field and assistant soccer referees during America’s cup. J Strength Cond Res. 2012 May;26(5):1383–8. doi: 10.1519/JSC.0b013e31825183c5 . 22395268

[3] Castagna C, Abt G. Intermatch var iation of match activity in elite Italian soccer referees. Journal of Strength and Conditioning Research. 2003;17(2):388–92. doi: 10.1519/1533-4287(2003)017&lt;0388:ivomai&gt;2.0.co;2 WOS:000183280400029. 12741883

[4] Muscella A, Stefano E, Di Maglie A, Marsigliante S. Referees’ physical performance over a soccer season. Sport Sciences for Health. 2020;16(4):765–73. doi: 10.1007/s11332-020-00655-1 WOS:000675815800021.

[5] Weston M, Castagna C, Impellizzeri FM, Rampinini E, Abt G. Analysis of physical match performance in English Premier League soccer referees with particular reference to first half and player work rates. Journal of Science and Medicine in Sport. 2007;10(6):390–7. doi: 10.1016/j.jsams.2006.09.001 WOS:000251036700007. 17126077

[6] Mascarenhas D. R. D., Button C., O’Hare D., & Dicks M. (2009). Physical performance and decision making in association football referees: A naturalistic study. *The Open Sports Sciences Journal*, 2(1).

[7] Martinez-Torremocha G, Martin-Sanchez ML, Garcia-Unanue J, Felipe JL, Moreno-Perez V, Paredes-Hernandez V, et al. Physical demands on professional Spanish football referees during matches. Science and Medicine in Football. doi: 10.1080/24733938.2022.2064539 WOS:000781930100001. 35392769

[8] Krustrup P, Bangsbo J. Physiological demands of top-class soccer refereeing in relation to physical capacity: effect of intense intermittent exercise training. Journal of Sports Sciences. 2001;19(11):881–91. doi: 10.1080/026404101753113831 WOS:000171355200007. 11695510

[9] Fernandes da Silva J, Teixeira AS, de Carvalho J, Do Nascimento Salvador P, Castagna C, Ventura AP, et al. Match activity profile and heart rate responses of top-level soccer referees during Brazilian national first and second division and regional championships. Sci Med Footb. 2023;7(3):263–71. Epub 20220726. doi: 10.1080/24733938.2022.2098372 . 35787742

[10] Yousefian F, Zafar A, Peres P, Brito J, Travassos B, Figueiredo P. Intensity demands and peak performance of elite soccer referees during match play. Journal of Science and Medicine in Sport. 2023;26(1):58–62. doi: 10.1016/j.jsams.2022.10.006 36344363

[11] Martinho DV, Field A, Rebelo A, Gouveia ER, Sarmento H. A Systematic Review of the Physical, Physiological, Nutritional and Anthropometric Profiles of Soccer Referees. Sports Med Open. 2023;9(1):72. Epub 20230810. doi: 10.1186/s40798-023-00610-7 ; PubMed Central PMCID: PMC10415246. 37561241 PMC10415246

[12] Aguirre-Loaiza H, Holguín J, Arenas J, et al. Psychological characteristics of sports performance: Analysis of professional and semiprofessional football referees[J]. Journal of Physical Education and Sport, 2020, 20(4): 1861–1868.

[13] Preissler AA, Reichert T, Schons P, Costa RR, Delevatti RS, Denadai B, et al. External Loads of Elite Soccer Referees: A Systematic Review with meta-analysis External loads of elite soccer referees. Research in Sports Medicine. doi: 10.1080/15438627.2021.1988948 WOS:000706243700001. 34633255

[14] Page MJ, McKenzie JE, Bossuyt PM, Boutron I, Hoffmann TC, Mulrow CD, et al. The PRISMA 2020 statement: An updated guideline for reporting systematic reviews. PLoS Med. 2021;18(3):e1003583. Epub 20210329. doi: 10.1371/journal.pmed.1003583 ; PubMed Central PMCID: PMC8007028. 33780438 PMC8007028

[15] Gooding HC, Gidding SS, Moran AE, Redmond N, Allen NB, Bacha F, et al. Challenges and Opportunities for the Prevention and Treatment of Cardiovascular Disease Among Young Adults: Report From a National Heart, Lung, and Blood Institute Working Group. J Am Heart Assoc. 2020;9(19):e016115. Epub 20200930. doi: 10.1161/JAHA.120.016115 ; PubMed Central PMCID: PMC7792379. 32993438 PMC7792379

[16] D’Ottavio S, Castagna C. Physiological load imposed on elite soccer referees during actual match play. Journal of Sports Medicine and Physical Fitness. 2001;41(1):27–32. WOS:000168408700004. 11405189

[17] Costa EC, Vieira CMA, Moreira A, Ugrinowitsch C, Castagna C, Aoki MS. Monitoring External and Internal Loads of Brazilian Soccer Referees during Official Matches. Journal of Sports Science and Medicine. 2013;12(3):559–64. WOS:000323603800027. 24149165 PMC3772602

[18] Riiser A, Pettersen SA, Andersen V, Saeterbakken AH, Froyd C, Ylvisaker E, et al. Accelerations and high intensity running in field and assistant football referees during match play. Science and Medicine in Football. 2017;1(3):280–7. doi: 10.1080/24733938.2017.1341640 WOS:000563963800014.

[19] Krustrup P, Helsen W, Randers MB, Christensen JF, MacDonald C, Rebelo AN, et al. Activity profile and physical demands of football referees and assistant referees in international games. Journal of Sports Sciences. 2009;27(11):1167–76. doi: 10.1080/02640410903220310 WOS:000270424700009. 19705329

[20] Sánchez MLM, Oliva-Lozano JM, García-Unanue J, Krustrup P, Felipe JL, Moreno-Pérez V, et al. Association between Fitness Level and Physical Match Demands of Professional Female Football Referees. Int J Environ Res Public Health. 2022 Aug 28;19(17):10720. doi: 10.3390/ijerph191710720 ; PMCID: PMC9518354. 36078434 PMC9518354

[21] Ramos-Cano J, Martin-Garcia A, Rico-Gonzalez M. Training intensity management during microcycles, mesocycles, and macrocycles in soccer: A systematic review[J]. Proceedings of the Institution of Mechanical Engineers, Part P: Journal of Sports Engineering and Technology, 2022: 17543371221101227.

[22] Catterall C, Reilly T, Atkinson G, Coldwells A. Analysis of the work rates and heart rates of association football referees. Br J Sports Med. 1993 Sep;27(3):193–6. doi: 10.1136/bjsm.27.3.193 ; PMCID: PMC1332186. 8242278 PMC1332186

[23] Stølen T, Chamari K, Castagna C, Wisløff U. Physiology of soccer: an update. Sports Med. 2005;35(6):501–36. doi: 10.2165/00007256-200535060-00004 . 15974635

