# Supplementary material for: Physical demands and physiological response of soccer referees in high-level matches: A systematic review
Source: PLoS One. 2025 Jan 24;20(1):e0315403. doi: 10.1371/journal.pone.0315403 (PMC11759383; doi:10.1371/journal.pone.0315403)
Supplement: S1 Table — (PDF) [file pone.0315403.s001.pdf]

S1 Table. Detailed search strategy

Database	Complete Search Strategy	20 Hits Oct 2023
Web of Science	((AB = （"Football" OR "Soccer") AND AB = ("Referee" OR " Match Official?" OR "Arbitrator", OR "Umpire") AND AB = ("Physical load" OR "Physical performance" OR "Physical demand" OR "Fitness demand" OR "Physical Profile" OR “Match activity” OR "activity profile" OR "Physical activity" OR "kinematical activity" OR "Physical exertion" OR "activity pattern" OR "Movement pattern" OR "physiological characteristics" OR "physiological profile" OR "energy expenditure" OR "speed", OR "sprint", OR "endurance", OR "yo-yo intermittent", OR "aerobic", OR "high intensity"))	254
Scopus	TITLE- ABS- KEY （"Football" OR "Soccer" AND "Referee" OR " Match Official?" OR "Arbitrator", OR "Umpire") AND "Physical load" OR "Physical performance" OR "Physical demand" OR "Fitness demand" OR "Physical Profile" OR “Match activity” OR "activity profile" OR "Physical activity" OR "kinematical activity" OR "Physical exertion" OR "activity pattern" OR "Movement pattern" OR "physiological characteristics" OR "physiological profile" OR "energy expenditure" OR "speed", OR "sprint", OR "endurance", OR "yo-yo intermittent", OR "aerobic", OR "high intensity")	294
PubMed	(("Football" OR "Soccer" [Title/Abstract]) AND ("Referee" OR " Match Official?" OR “Arbitrator", OR "Umpire" [Title/Abstract]) AND ("Physical load" OR "Physical performance" OR "Physical demand" OR "Fitness demand" OR "Physical Profile" OR “Match activity” OR "activity profile" OR "Physical activity" OR "kinematical activity" OR "Physical exertion" OR "activity pattern" OR "Movement pattern" OR "physiological characteristics" OR "physiological profile" OR "energy expenditure" OR "speed", OR "sprint", OR "endurance", OR "yo-yo intermittent", OR "aerobic", OR "high intensity" [Title/Abstract])	281
EBSCOhost	AB ("Football" OR "Soccer") AND AB ("Referee" OR " Match Official?" OR “Arbitrator", OR "Umpire") AND AB ("Physical load" OR "Physical performance" OR "Physical demand" OR "Fitness demand" OR "Physical Profile" OR “Match activity” OR "activity profile" OR "Physical activity" OR "kinematical activity" OR "Physical exertion" OR "activity pattern" OR "Movement pattern" OR "physiological characteristics" OR "physiological profile" OR "energy expenditure" OR "speed", OR "sprint", OR "endurance", OR "yo-yo intermittent", OR "aerobic", OR "high intensity"))	1045
