# Supplementary material for: Physical demands and physiological response of soccer referees in high-level matches: A systematic review
Source: PLoS One. 2025 Jan 24;20(1):e0315403. doi: 10.1371/journal.pone.0315403 (PMC11759383; doi:10.1371/journal.pone.0315403)
Supplement: S1 File — (PDF) [file pone.0315403.s002.pdf]

S1 File. Studies Identification

ID	Exclusion or Inclusion	Publish Information
1	Selected	D'ottavio and Castagna.2001Physiological load imposed on elite soccer referees during actul match play
2	Not comparisons in the time phase	.A. Harley, K. Tozer, J. Doust.An analysis of movement patterns and physiological strain in relation to optimal positioning of association football referees
3	Not relevant	‘They kick you because they are not able to kick the ball’: normative conceptions of sex difference and the politics of exclusion in mixed-sex football. Vol. 8/9 1332-1348 (2016).
4	Not relevant	1996-2020: SUPER RUGBY IS TURNING 25: GIFT THAT KEEPS GIVING: 10 MOST INFLUENTIAL PLAYERS. NZ Rugby World, 60-71 (2020).
5	Not relevant	25 WORLD CUP CONTROVERSIES. NZ Rugby World, 84-95 (2019).
6	Not relevant	4th International Conference on Human Performance Development through Strength and Conditioning, NSCA 2014.
7	Not relevant	A, D.E.C., et al. Anterior cruciate ligament injury in elite football players: video analysis of 128 cases. "J Sports Med Phys Fitness,[2020#5Year]IF->1.432#1.451" 62, 222-228 (2022).
8	Not relevant	A, U., J. P, V., C, L. & B, W. Cardiovascular Stress in Football Referees. German Journal of Sports Medicine / Deutsche Zeitschrift fur Sportmedizin 73, 30-34 (2022).
9	Not relevant	Abdula, A., Pertsukhov, A., Wnorowski, K., Mozharovska, S. & Mozharovskyy, Y. Features of the development of strength training of highly qualified football referees in the competitive period.
10	Not relevant	Abián, P., et al. Muscle damage produced during a simulated badminton match in competitive male players. "Res Sports Med,[2020#5Year]IF->2.554#2.410" 24, 104-117 (2016).
11	Not relevant	Abian-Vicen, J., et al. Influence of Successive Badminton Matches on Muscle Strength, Power, and Body-Fluid Balance in Elite Players. International Journal of Sports Physiology & Performance 9, 689-694 (2014).

S1 File. Studies Identification

12	Not relevant	Abstracts from the 17th Annual Scientific Conference of Montenegrin Sports Academy "Sport, Physical Activity and Health: Contemporary Perspectives": Cavtat, Dubrovnik, Croatia. 2-5 April 2020. Montenegrin Journal of Sports Science & Medicine 9, 5-37 (2020).
13	Not relevant	ABSTRACTS FROM THE 3RD WORLD CONGRESS OF SPORTS PHYSICAL THERAPY. International Journal of Sports Physical Therapy 14, S1-S28 (2019).
14	Not relevant	Abstracts of the 2015 CSEP General Meeting / Résumés de la 2015 Congrès annuelle de la SCPE. Applied Physiology, Nutrition & Metabolism 40, S1-S69 (2015).
15	Not relevant	Abt, G. & Lovell, R. The use of individualized speed and intensity thresholds for determining the distance run at high-intensity in professional soccer. "J Sports Sci,[2020#5Year]IF->2.597#3.060" 27, 893-898 (2009).
16	Not relevant	Achenbach, L., et al. Collision with opponents—but not foul play—dominates injury mechanism in professional men's basketball. BMC Sports Science, Medicine & Rehabilitation 13, 1-11 (2021).
17	Not relevant	Adair, D. COMPETING OR COMPLEMENTARY FORCES? THE 'CIVILISING' PROCESS AND THE COMMITMENT TO WINNING IN NINETEENTH CENTURY ENGLISH RUGBY AND ASSOCIATION FOOTBALL. Canadian Journal of History of Sport 24, 47-67 (1993).
18	Not relevant	Adama, T., et al. Changes in performance on physical capacity tests after three months of cross-training among elite male football referees in Burkina Faso.
19	Not relevant	Adán, L., García-Angulo, A., Gómez-Ruano, M.A., Sainz de Baranda, P. & Ortega-Toro, E. ANÁLISIS BIBLIOMÉTRICO DE LA PRODUCCIÓN CIENTÍFICA EN FÚTBOL FEMENINO. Journal of Sport & Health Research 12, 302-316 (2020).
20	Not relevant	Adie, J.W., Duda, J.L. & Ntoumanis, N. FREE COMMUNICATIONS. in Journal of Sport & Exercise Psychology S23-S198.

S1 File. Studies Identification

21	Not relevant	Adnan, R., Muzayin, N. & Sulaiman, N. Analysis of Movement Pattern among Referee in 2012 Malaysian Cup. Journal of Human Sport & Exercise 8, S642-S650 (2013).
22	Not comparisons in the time phase	Adnan, R., Muzayin, N., & Sulaiman, N.Analysis of Movement Pattern among Referee in 2012 Malaysian Cup
23	Not relevant	Afyon, Y.A., CİNdemİR, V. & MÜLazimoĞLu, O. SÜRAT VE ÇEVİKLİK ANTRENMANLARININ FUTBOL HAKEMLERİNİN BAZI FİZİKSEL VE MOTORİK ÖZELLİKLERİNE ETKİSİ. Congress Papers of The Association of Sports Sciences, 369-374 (2018).
24	Not relevant	Ağduman, F. & Bedir, D. From Past to Present The Most Mysterious Olympic Sport: A Review of Curling Sport. Online Journal of Recreation & Sports 12, 314-325 (2023).
25	Not relevant	Aguirre-Loaiza, H., et al. Psychological characteristics of sports performance: analysis of professional and semiprofessional football referees. Journal of Physical Education & Sport 20, 1861-1868 (2020).
26	Not relevant	Ahmad, M.F., Hussain, R.N.J.R., Chik, W.F.W. & Halim, M.H.A. Analysis of goal scoring in Copa America 2021. Journal of Physical Education & Sport 22, 2393-2398 (2022).
27	Not relevant	Ahmed, H.S., Marcora, S.M., Dixon, D. & Davison, G. The Effect of a Competitive Futsal Match on Psychomotor Vigilance in Referees. International Journal of Sports Physiology & Performance 15, 1297-1302 (2020).
28	Not relevant	Ahmed, H; Davison, G; Dixon, D.Analysis of activity patterns, physiological demands and decision-making performance of elite Futsal referees during matches
29	Not physical or physiological variables	Ai, K., Oldrich, R., Tan, H. & Xu, P. Sustainable innovation in football referee training in Czech Republic.
30	Not relevant	Ai, K; Oldrich, R; Tan, H; Xu, P.Sustainable Innovation in Football Referee Training in Czech Republic

S1 File. Studies Identification

31	Not relevant	Aksum, K.M., et al. Scanning activity in elite youth football players. "J Sports Sci,[2020#5Year]IF->2.597#3.060" 39, 2401-2410 (2021).
32	Not relevant	AktaŞ, İ. & Sezen-BalÇIkanli, G. I Can, But I Don't! Coaches' Sportsmanship Dilemma. Mediterranean Journal of Sport Science (MJSS) 6, 981-994 (2023).
33	Not relevant	Al Attar, W.S.A., et al. Effectiveness of the FIFA 11+ Referees Injury Prevention Program in reducing injury rates in male amateur soccer referees. Scandinavian Journal of Medicine & Science in Sports 31, 1774-1781 (2021).
34	Not relevant	Albaladejo-García, C., Caballero, C., Asencio, P. & Moreno, F.J. Acute effects of a vigorous-intensity warm-up on response suppression and decision-making of football referees. "J Sports Sci,[2020#5Year]IF->2.597#3.060" 42, 1081-1089 (2024).
35	Not relevant	Albrecht, J., Nagel, S. & Klenk, C. "Just taking part or fully participate with others!?": Social integration of members with disabilities in mainstream sports clubs. Sport und Gesellschaft 18, 253-279 (2021).
36	Not relevant	AleksiĆ VeljkoviĆ, A., ĐUroviĆ, D., Biro, F., StojanoviĆ, K. & IliĆ, P. EATING ATTITUDES AND BODY IMAGE CONCERNS AMONG FEMALE ATHLETES FROM AESTHETIC SPORTS. Annales Kinesiologiae 11, 3-16 (2020).
37	Not relevant	Alexander, F., Tucker, R., Jones, B. & Hendricks, S. X as a proxy for tackle safety culture? Sentiment analysis of social media posts on red-carded and yellow-carded tackles during the 2019 Rugby World Cup. BMJ Open Sport Exerc Med 9, e001756 (2023).
38	Not relevant	Alexander, J.P., Spencer, B., Sweeting, A.J., Robertson, S. & Mara, J.K. The influence of match phase and field position on collective team behaviour in Australian Rules football. "J Sports Sci,[2020#5Year]IF->2.597#3.060" 37, 1699-1707 (2019).
39	Not relevant	Alimoradi, M., Hosseini, E., Iranmanesh, M., Monfaredian, O. & Kozinc, Ž. Effect of 24-Week FIFA 11+ Referees Program on Quality of Change of Direction Maneuver in Elite Soccer Referees.
40	Not relevant	AlKhalifa, H.K. & Farello, A. The soft power of Arab women's football: changing perceptions and building legitimacy through social media. International Journal of Sport Policy & Politics 13, 241-257 (2021).

S1 File. Studies Identification

41	Not relevant	Allami, N.A.H., Hussein, G.A. & Al-Lami, K.K.R. The effect of selective exercises on developing motor abilities, basic skills, and mental perception among football players. Journal of Human Sport & Exercise 16, S1425-S1433 (2021).
42	Not relevant	Allard, F. & Starkes, J.L. Perception in Sport: Volleyball. Journal of Sport Psychology 2, 22-33 (1980).
43	Not relevant	Allen, J.B. & Reid, C. Scaffolding Women Coaches' Development: A Program to Build Coaches' Competence and Confidence. Women in Sport & Physical Activity Journal 27, 101-109 (2019).
44	Not relevant	Alley, L.E. Two Paths to Excellence. in Quest (00336297), Vol. 34 99-108 (1982).
45	Not relevant	Allister, A., Byrne, P.J., Nulty, C.D. & Jordan, S. Game-related statistics which discriminate elite senior Gaelic football teams according to game outcome and final score difference. "International Journal Of Performance Analysis In Sport,[2020#5Year]IF->1.518#1.707" 18, 622-632 (2018).
46	Not relevant	Alsarve, D. & Johansson, E. A gang of ironworkers with the scent of blood: A participation observation of male dominance and its historical trajectories at Swedish semi-professional ice hockey events. "International Review For The Sociology Of Sport,[2020#5Year]IF->2.019#1.972" 57, 54-72 (2022).
47	Not relevant	Alseiari, S.R., Al Qatawneh, I.S., Kandeel, M.E. & Moussa, A.F. The player's penal responsibility for the actions of violence in sports: legal analysis in context of United Arab Emirates law. Physical Activity Review 10, 51-65 (2022).
48	Not relevant	Alsharji, K.E., Alkatan, M.F., Akbar, A.A. & Ahmad, H.A. THE EFFECT OF REFEREEING AND PLAYING EXPERIENCE ON THE LEVEL OF SELFEFFICACY FOR HANDBALL REFEREES: NATIONAL AND INTERNATIONAL REFEREES. Journal of Development & Research for Sport Science Activities (JDRASSA) 5, 210-226 (2019).
49	Not relevant	Alvira, DC; Tobalina, JC; Iragoyen, JY.Analysis of cardiac response of soccer referees in competition: a case study
50	book	Al-Wattar, N.S.Y., Hussein, F. & Hussein, A.A. Women’s narratives of sport and war in Iraq.

S1 File. Studies Identification

51	Not relevant	Åman, M., Forssblad, M. & Larsén, K. National injury prevention measures in team sports should focus on knee, head, and severe upper limb injuries. Knee Surgery, Sports Traumatology, Arthroscopy 27, 1000-1008 (2019).
52	Not relevant	Amatori, S., et al. High-Speed Efforts of Elite Association Football Referees in National and International Matches.
53	Not relevant	Amatria, M., Lapresa, D., Arana, J., Anguera, M.T. & Jonsson, G.K. Detection and Selection of Behavioral Patterns Using Theme: A Concrete Example in Grassroots Soccer. Sports (2075-4663) 5, 20 (2017).
54	Not relevant	Amini Farsani, M., Shahbazi, M. & Tahmasebi Boroujeni, S. Improvement in soccer goalkeepers' spatial anticipation during penalty kicks as a result of PETTLEP imagery intervention. Journal of Imagery Research in Sport & Physical Activity 18, 1-6 (2023).
55	Not relevant	ANAIS 10º SOCCER EXPERIENCE - 2022. Brazilian Journal of Soccer Science / Revista Brasileira de Futebol 15, 1-53 (2022).
56	Not relevant	Anderson, D., Breed, R., Spittle, M. & Larkin, P. Factors Affecting Set Shot Goal-kicking Performance in the Australian Football League. Perceptual & Motor Skills 125, 817-833 (2018).
57	Not relevant	Andersson, S.H., et al. Video analysis of acute injuries and referee decisions during the 24th Men's Handball World Championship 2015 in Qatar. Scandinavian Journal of Medicine & Science in Sports 28, 1837-1846 (2018).
58	Not relevant	Ando, K., et al. Japanese Female Professional Soccer Players' Views on Second Career Development. Women in Sport & Physical Activity Journal 30, 151-160 (2022).
59	Not relevant	Annual Conference of the British Association of Sport and Exercise Sciences. "J Sports Sci,[2020#5Year]IF->2.597#3.060" 23, 93-223 (2005).
60	Not relevant	Añorve Añorve, D. Gender social change, international influence and sensemaking in the launch and evolution of the Mexican professional women's football league. "Sport in Society,[2020#5Year]IF->0.939#Not Available" 24, 1122-1140 (2021).

S1 File. Studies Identification

61	Not relevant	Antonie, A. Career stages in romanian football refereeing - the path from referee course to the FIFA Badge. Journal of Physical Education & Sport 22, 1675-1684 (2022).
62	Not relevant	Antonie, A. Stressors in Romanian elite football refereeing-- a comparison between 2009 and 2020. Journal of Physical Education & Sport 23, 186-193 (2023).
63	Not relevant	Antonio, B. Injury rate in elite principal team sports after COVID-19 Lockdown: A literature review. SEMS-Journal 71, 35-42 (2023).
64	Not relevant	Araújo, R., Delgado, M., Azevedo, E. & Mesquita, I. STUDENTS' TACTICAL UNDERSTANDING DURING A HYBRID SPORT EDUCATION/STEPGAME APPROACH MODEL VOLLEYBALL TEACHING UNIT. Movimento (0104754X) 26, 1-16 (2020).
65	Not relevant	Arboix-Alió, J. & Aguilera-Castells, J. ANÁLISIS DE LA INFLUENCIADE JUGAR COMO LOCAL EN HOCKEY SOBRE PATINES. Journal of Sport & Health Research 11, 263-272 (2019).
66	Not relevant	Arboix-Alió, J., Buscà, B., Trabal, G., Aguilera-Castells, J. & Sánchez-López, M.J. Comparison of home advantage in men's and women's Portuguese roller hockey league. Cuadernos de Psicología del Deporte 20, 181-189 (2020).
67	Not relevant	Arboix-Alió, J., et al. Situational and Game Variables in Rink Hockey: A Systematic Review. Apunts: Educació Física i Esports, 22-35 (2023).
68	Not relevant	Arboix-Alió, J., et al. The influence of contextual variables on individual set-pieces in elite rink hockey. "International Journal Of Performance Analysis In Sport,[2020#5Year]IF->1.518#1.707" 21, 336-347 (2021).
69	Not relevant	Ardigò, L.P. Low-cost match analysis of Italian sixth and seventh division soccer refereeing.
70	Not relevant	Ardigò, L.P., Padulo, J., Zuliani, A. & Capelli, C. A low-cost method for estimating energy expenditure during soccer refereeing. "J Sports Sci,[2020#5Year]IF->2.597#3.060" 33, 1853-1858 (2015).
71	Not relevant	Arguedas-Soley, A., Townsend, I., Hengist, A. & Betts, J. Acute caffeine supplementation and live match-play performance in team-sports: A systematic review (2000–2021). "J Sports Sci,[2020#5Year]IF->2.597#3.060" 40, 717-732 (2022).

S1 File. Studies Identification

72	Not relevant	Arjona, C.M., Fernandes, B.R., Dos Santos, T.D., Onetti, W.O. & Rodriguez, A.C. Effect of the soccer referee's experience in sports competition.
73	Not relevant	Arundale, A.J.H., et al. Exercise-Based Knee and Anterior Cruciate Ligament Injury Prevention. Journal of Orthopaedic & Sports Physical Therapy 53, CPG1-CGP34 (2023).
74	Not relevant	Ashford, M., Burke, K., Barrell, D., Abraham, A. & Poolton, J. The impact of rule modifications on player behaviour in a talent identification and development environment: A case study of the Rugby Football Union's Wellington Academy Rugby Festival. "J Sports Sci,[2020#5Year]IF->2.597#3.060" 38, 2670-2676 (2020).
75	Not relevant	Aslan, M. & UĞRaŞ, S. TENDENCY OF VIOLENCE IN TEAM SPORTS ATHLETES: A SCALE DEVELOPMENT STUDY. SPORMETRE: The Journal of Physical Education & Sport Sciences / Beden Eğitimi ve Spor Bilimleri Dergisi 19, 156-167 (2021).
76	Not relevant	Asselstine, S. & Edwards, J.R. Managing the resource capabilities of provincial Rugby unions in Canada: an understanding of competitive advantage within a sport development system. Managing Sport & Leisure 24, 78-96 (2019).
77	Not relevant	Aston Seng Huey, N., et al. Fractures in professional footballers: 7-years data from 106 team seasons in the Middle East. "Biol Sport,[2020#5Year]IF->2.000#2.250" 40, 1117-1124 (2023).
78	Not relevant	Atifi, J. & Lotfi, S. The effect of audience presence on home advantage: An analysis of two seasons in the Moroccan professional soccer league. Journal of Physical Education & Sport 24, 1116-1122 (2024).
79	Not relevant	Atkinson, G., et al. PART II: INTERDISCIPLINARY. "J Sports Sci,[2020#5Year]IF->2.597#3.060" 19, 14-30 (2001).
80	Not relevant	Augusto, D., et al. Within-subject variation of technical performance in elite Brazilian professional soccer players: the environmental stress, match location, and opposition's ranking influences. "International Journal Of Performance Analysis In Sport,[2020#5Year]IF->1.518#1.707" 22, 583-593 (2022).

81	Not relevant	Augustovicova, D., Argajova, J., Rupcik, L. & Thomson, E. Development of a reliable and valid kata performance analysis template. Journal of Physical Education & Sport 20, 3553-3559 (2020).
82	Not relevant	Avakian, P., et al. Development and reliability of technical-tactical and time-motion real-time analysis in the World Taekwondo Grand Prix. Ido Movement for Culture. Journal of Martial Arts Anthropology 21, 20-27 (2021).
83	Not relevant	Avalos, B.L. "Friday Night is their Super Bowl": A Relational Investigation Regarding Occupational Stress Among American High School Football Officials. Communication & Sport 11, 156-174 (2023).
84	Not relevant	Avner, Z., Denison, J., Jones, L., Boocock, E. & Hall, E.T. Beat the Game: a Foucauldian exploration of coaching differently in an elite rugby academy. Sport, Education & Society 26, 676-691 (2021).
85	Not relevant	Avner, Z., Hall, E.T. & Potrac, P. Affect and emotions in sports work: a research agenda. "Sport in Society,[2020#5Year]IF->0.939#Not Available" 26, 1161-1177 (2023).
86	Not relevant	Avugos, S., Zach, S., Dvir Malca, A. & Bar-Eli, M. An intimate glance into "The Holiest of Holies": An exploratory study of the half-time talk. International Journal of Sport & Exercise Psychology 18, 129-142 (2020).
87	Review	Avugos, S; MacMahon, C; Bar-Eli, M; Raab, M.Inter-individual differences in sport refereeing: A review of theory and practice
88	Not relevant	AydemİR, B., Kul, M. & KirkbİR, F. Taekwondocuların Psikolojik Sağlamlık Düzeyleri İle Durumluk ve Sürekli Kaygı Düzeyleri Arasındaki İlişkinin İncelenmesi. Mediterranean Journal of Sport Science (MJSS) 6, 1249-1260 (2023).
89	Not relevant	Azar, O.H. & Bar-Eli, M. Penalty kicks as cross-fertilization: On the economic psychology of sports. Asian Journal of Sport & Exercise Psychology 3, 8-12 (2023).
90	Not relevant	Baena-GonzÁLez, R., MaríA Gallardo, A.N.A., ChavarríA-Ortiz, C. & GarcíA-TascÓN, M. Perception of safety in the game by teen handball players from a gender perspective using a novel anti-tip system. Journal of Physical Education & Sport 20, 3234-3244 (2020).

S1 File. Studies Identification

91	Not relevant	Bafirman, Zarya, F., Wahyuri, A.S., Ihsan, N. & Batubara, R. Improving the martial art skills and physical fitness quality of students grade VII through e-module development. Journal of Physical Education & Sport 23, 3271-3281 (2023).
92	Not relevant	Bahmani, M., Bahram, A., Diekfuss, J.A. & Arsham, S. An expert's mind in action: Assessing attentional focus, workload and performance in a dynamic, naturalistic environment. "J Sports Sci,[2020#5Year]IF->2.597#3.060" 37, 2318-2330 (2019).
93	Not relevant	Bailey, A. & Harris, K. An autoethnographic study of realist knowledge translation within sport development. Managing Sport & Leisure 26, 41-59 (2021).
94	Not relevant	Baker, D.G. & Newton, R.U. Discriminative Analyses of Various Upper Body Tests in Professional Rugby-League Players. International Journal of Sports Physiology & Performance 1, 347-360 (2006).
95	Not relevant	Baldwin, C. & Vallance, R. Community based rugby referees' experiences with recruitment and retention. International Sports Studies 38, 49-63 (2016).
96	Not relevant	Ball, S., Halaki, M. & Orr, R. Movement Demands and Injury Characteristics in Under-20-Years University Rugby Union Players. Journal of Athletic Training (Allen Press) 55, 376-383 (2020).
97	Not relevant	Bandyopadhyay, K. Introduction: COVID-19 and the soccer world. Soccer & Society 22, 1-7 (2021).
98	Not relevant	Bao, R. & Han, B. The influence of the video assistance referee (VAR) on the English Premier League. "International Journal Of Performance Analysis In Sport,[2020#5Year]IF->1.518#1.707" 24, 241-250 (2024).
99	Not relevant	Baptista, C., et al. Teaching personal and social responsibility after-school: A systematic review. Cuadernos de Psicología del Deporte 20, 1-25 (2020).
100	Selected	Barbero-Álvarez et al.2012Physical and physiological demands of field and assistant soccer referees during America's cup

S1 File. Studies Identification

101	Not relevant	Barberó-Álvarez, J.C., Boullosa, D., Nakamura, F.Y., Andrín, G. & Weston, M. Repeated acceleration ability (RAA): A new concept with reference to top-level field and assistant soccer referees.
102	Not relevant	Barbero-Álvarez, J.C., Boullosa, D.A., Nakamura, F.Y., Andrín, G. & Castagna, C. Physical and physiological demands of field and assistant soccer referees during America's cup.
103	Not relevant	Barbosa de Lira, C.A., et al. Heart rate response during a simulated Olympic boxing match is predominantly above ventilatory threshold 2: a cross sectional study. Open Access Journal of Sports Medicine 4, 175-182 (2013).
104	Not relevant	Barfield, J.P., Williams, S., Currie, M.R. & Guo, X. Evidence-Based Classification in Powerchair Football: Determining the Determinants. "Adapt Phys Act Quart,[2020#5Year]IF->1.462#2.336" 39, 197-213 (2022).
105	Not relevant	Barkell, J.F., O'Connor, D. & Cotton, W.G. Effective strategies at the ruck in men's and women's World Rugby Sevens Series. International Journal of Sports Science & Coaching 13, 225-235 (2018).
106	Not relevant	Baross, A.W., et al. PART IV: PHYSIOLOGY. "J Sports Sci,[2020#5Year]IF->2.597#3.060" 20, 45-60 (2002).
107	Not relevant	Barquero-Ruiz, C., Kirk, D. & Arias-Estero, J.L. Design and Validation of the Tactical Assessment Instrument in Football (TAIS). Research Quarterly for Exercise & Sport 93, 615-632 (2022).
108	Not relevant	Barranco-Ruiz, Y., Villa-González, E., Martínez-Amat, A. & Da Silva-Grigoletto, M.E. Prevalence of Injuries in Exercise Programs Based on Crossfit®, Cross Training and High-Intensity Functional Training Methodologies: A Systematic Review. "Journal Of Human Kinetics,[2020#5Year]IF->1.664#1.886" 73, 251-265 (2020).
109	Not relevant	Barreira, J. Vantagem de jogar em casa no futebol feminino: uma análise de três importantes campeonatos no Brasil. Revista Brasileira de Ciência e Movimento: RBCM 26, 83-87 (2018).
110	Not relevant	Barte, J.C.M. & Oudejans, R.R.D. The Effects of Additional Lines on a Football Field on Assistant Referees' Positioning and Offside Judgments. International Journal of Sports Science & Coaching 7, 481-492 (2012).

S1 File. Studies Identification

111	Not relevant	Barth, J., et al. Is technical performance related to the distance covered during U-19 futsal matches? Brazilian Journal of Kineanthropometry & Human Performance 21, 1-11 (2019).
112	Not relevant	Bartha, C., Petridis, L., Hamar, P., Puhl, S. & Castagna, C. Fitness test results of Hungarian and international-level soccer referees and assistants. "J Strength Cond Res,[2020#5Year]IF->2.973#3.058" 23, 121-126 (2009).
113	Not relevant	Bases 2013 Abstracts. "J Sports Sci,[2020#5Year]IF->2.597#3.060" 32, s4-s116 (2014).
114	Not relevant	BASES Conference 2019 – Programme and Abstracts. in "J Sports Sci,[2020#5Year]IF->2.597#3.060", Vol. 37 1-93.
115	Not relevant	BaŞKaya, G. & MetİN, S.N. Evaluation of The Effect of The VAR System on The Matches Played in the 2022 FIFA World Cup and UEFA Women's EURO 2022. Journal of Sports Sciences Research / Spor Bilimleri Araştırmaları Dergisi 8, 486-499 (2023).
116	Not relevant	Bassek, M., Raabe, D., Banning, A., Memmert, D. & Rein, R. Analysis of contextualized intensity in Men's elite handball using graph-based deep learning. "J Sports Sci,[2020#5Year]IF->2.597#3.060" 41, 1299-1308 (2023).
117	Not relevant	Battaglia, A. & Kerr, G. Exploring Youth Sport Coaches' Perspectives on the Use of Benching as a Behavioral Management Strategy. International Sport Coaching Journal 10, 266-275 (2023).
118	Not relevant	Bauer, T. & Lin, S. Ping-pong at the cinema: humor, love and more. "Sport in Society,[2020#5Year]IF->0.939#Not Available" 25, 1847-1862 (2022).
119	Not relevant	Baxter, H., Kappelides, P. & Hoye, R. Female volunteer community sport officials: a scoping review and research agenda. "European Sport Management Quarterly,[2020#5Year]IF->1.889#2.436" 23, 429-446 (2023).
120	Not relevant	Baydemir, B; Yurdakul, HÖ; Aksoy, S.The Effect of Different Training Strategies Applied to Football Referees On Maxvo2 and Running Performance

S1 File. Studies Identification

121	Not relevant	Bayón, P., Vaquera, A., García-Tormo, J.V. & Dehesa, R. Efectos del entrenamiento en la habilidad para repetir sprints (RSA) en arbitros de baloncesto. Cuadernos de Psicología del Deporte 15, 163-168 (2015).
122	Not relevant	Bean, J. OFF THE WALLS. Referee, 32-34 (2010).
123	Not relevant	Beare, H. & Stone, J.A. Analysis of attacking corner kick strategies in the FA women's super league 2017/2018. "International Journal Of Performance Analysis In Sport,[2020#5Year]IF->1.518#1.707" 19, 893-903 (2019).
124	Not relevant	Beauchamp, M.K., Harvey, R.H. & Beauchamp, P.H. An Integrated Biofeedback and Psychological Skills Training Program for Canada's Olympic Short-Track Speedskating Team. "Journal of Clinical Sport Psychology,[2020#5Year]IF->1.270#Not Available" 6, 67-84 (2012).
125	Not relevant	Beckman, E.M., James, L.P. & Kelly, V.G. REVIEW OF THE LITERATURE | HIGH PERFORMANCE TESTING FOR THE ELITE MIXED MARTIAL ARTIST. Journal of Australian Strength & Conditioning 22, 81-98 (2014).
126	Not relevant	Behrens, A. & Uhrich, S. You'll never want to watch alone: the effect of displaying in-stadium social atmospherics on media consumers' responses to new sport leagues across different types of media. "European Sport Management Quarterly,[2020#5Year]IF->1.889#2.436" 22, 120-138 (2022).
127	Not relevant	Belalcazar, C., Hernández Nariño, T. & Callary, B. An Individualized Coach Development Program for Older Adult Player-Coaches in a Masters Football League in Colombia. Journal of Aging & Physical Activity 31, 117-127 (2023).
128	Not relevant	Belcic, I; Ruzic, L; Marosevic, A.Influence of functional abilities on the quality of refereeing in handball
129	Not relevant	Belka, J., Hulka, K., Safar, M., Weisser, R. & Samcova, A. ANALYSES OF TIME-MOTION AND HEART RATE IN ELITE FEMALE PLAYERS (U19) DURING COMPETITIVE HANDBALL MATCHES. "Kinesiology,[2020#5Year]IF->1.225#1.316" 46, 33-43 (2014).

S1 File. Studies Identification

130	Not relevant	BĚLka, J.A.N., HŮLka, K., ŠAfÁŘ, M., Weisser, R. & Mikova, L. Analysis of the fitness level in elite handball players (U16 and U18) between 2003 and 2013. Journal of Physical Education & Sport 16, 1381-1390 (2016).
131	Not relevant	Bendala, F.J.T., Vázquez, M.A.C., Suarez-Arrones, L.J. & Sánchez, F.J.N. Comparison of external load in high speed actions between friendly matches and training sessions. Retos: Nuevas Perspectivas de Educación Física, Deporte y Recreación 33, 54-57 (2018).
132	Not relevant	Bennett, G., Keiper, P. & Dixon, M. Why Can't We All Just Get Along? The Conflict between School and Club Sports in the United States. Quest (00336297) 72, 85-101 (2020).
133	Not relevant	Benrabah, K., Bennadja, M. & Fayçal, K.M. The Level of Community Cohesiveness Under Psychological Pressure and Control Center for Emerging Football Players U17. Acta Facultatis Educationis Physicae Universitatis Comenianae 60, 44-54 (2020).
134	Not relevant	Berg, A. The compatibility of zero-sum logic and mutualism in sport. "J Phil Sport,[2020#5Year]IF->0.867#0.814" 45, 259-278 (2018).
135	Not relevant	Bergin, J. & Lagestad, P. Dropping Out or Continuing Playing—A Case Study of Adolescent's Motives for Participation in Football. Sports (2075-4663) 11, 128 (2023).
136	Not relevant	Bergmann, F., Braksiek, M. & Meier, C. The influence of different game formats on technical actions and playing time parameters – A study with under-7 and under-9 soccer players in a competitive context. International Journal of Sports Science & Coaching 17, 1089-1100 (2022).
137	Not relevant	Berhimpong, M.W., et al. Exploring the impact of drills training and grip strength on tennis serve performance: A factorial experimental design research. Journal of Physical Education & Sport 23, 3108-3118 (2023).
138	Not relevant	Berman, M. Sprints, Sports, and Suits. "J Phil Sport,[2020#5Year]IF->0.867#0.814" 40, 163-176 (2013).
139	Not relevant	Besoluk, S.Determination of relationship between respiratory parameters and aerobic capacity of referees

S1 File. Studies Identification

140	Not relevant	Bessa Pereira, C., Farias, C., Gracinda Ramos, A., Coutinho, P. & Mesquita, I. Examining the interplay between performance development, game-play participation, and context of practice in two sport education invasion games seasons. Revista Portuguesa de Ciências do Desporto, 11-24 (2022).
141	Not relevant	Bessa, C., Hastie, P., Araújo, R. & Mesquita, I. What Do We Know About the Development of Personal and Social Skills within the Sport Education Model: A Systematic Review. Journal of Sports Science & Medicine 18, 812-829 (2019).
142	Not relevant	Bessa, C., Hastie, P., Ramos, A. & Mesquita, I. What Actually Differs between Traditional Teaching and Sport Education in Students' Learning Outcomes? A Critical Systematic Review. Journal of Sports Science & Medicine 20, 110-125 (2021).
143	Not relevant	Bessa, C., Hastie, P., Rosado, A. & Mesquita, I. Differences between sport education and traditional teaching in developing students' engagement and responsibility. Journal of Physical Education & Sport 20, 3536-3545 (2020).
144	Not relevant	Bester, C., Coetzee, D., Schall, R. & Blair, M. Physical demands on elite lead rugby union referees. "International Journal Of Performance Analysis In Sport,[2020#5Year]IF->1.518#1.707" 19, 258-273 (2019).
145	Not relevant	Beyer, JA; Rowson, S; Duma, SM.Concussions Experienced by Major League Baseball Catchers and Umpires: Field Data and Experimental Baseball Impacts
146	Not relevant	Birinci, MC; Yilmaz, AK; Erkin, A; Sahbaz, S; Aydin, I.Determination of relationship between respiratory parameters and aerobic capacity of referees
147	Review	Birk Preissler, A.A., et al. External Loads of Elite Soccer Referees: A Systematic Review with meta-analysis: External loads of elite soccer referees. "Res Sports Med,[2020#5Year]IF->2.554#2.410" 31, 342-356 (2023).
148	book	Biswas, R. Continuous fuzzy evaluation methods: A novel tool for the analysis and decision making in football (or soccer) matches: A new innovative proposal to FIFA & UEFA.
149	Not relevant	Bizzini, M., Schmied, C., Junge, A. & Dvorak, J. Precompetition medical assessment of referees and assistant referees selected for the 2010 FIFA World Cup.

S1 File. Studies Identification

150	Not relevant	Blair, M.R., Elsworthy, N., Rehrer, N.J., Button, C. & Gill, N.D. Physical and Physiological Demands of Elite Rugby Union Officials. International Journal of Sports Physiology & Performance 13, 1199-1207 (2018).
151	Not relevant	Blair, M.R., Manuel-Hepi, N., Newman, L., Elsworthy, N. & Smith, T.B. PHYSICAL DEMANDS OF INTERNATIONAL LEAD RUGBY UNION REFEREES. Journal of Australian Strength & Conditioning 27, 24-31 (2019).
152	Not relevant	Blair, M.R., Scanlan, A.T., Lastella, M., Ramsey, C. & Elsworthy, N. The relationships between physical fitness attributes and match demands in rugby union referees officiating the 2019 Rugby World Cup. "International Journal Of Performance Analysis In Sport,[2020#5Year]IF->1.518#1.707" 22, 183-194 (2022).
153	Review	Blair, MR; Cronin, JB; Rehrer, NJ; Button, C; Gill, ND. Contextual Review of Physical Requirements of Refereeing Rugby Union at an Elite Level
154	Not relevant	Blasco, M.J., et al. INJURIES IN TRADITIONAL WRESTLING: THE LEONESE WRESTLING CASE STUDY (2005-2015). International Journal of Medicine & Science of Physical Activity & Sport / Revista Internacional de Medicina y Ciencias de la Actividad Física y del Deporte 18, 723-736 (2018).
155	Not relevant	Blazevich, A., Cronin, N. & Wells, S. Annual Conference of the British Association of Sport and Exercise Sciences. "J Sports Sci,[2020#5Year]IF->2.597#3.060" 23, 1149-1303 (2005).
156	Not relevant	Blomqvist Mickelsson, T. & Shaw, V. Performance increments without audience: paired data from mixed martial arts fighters during COVID-19. Revista de Artes Marciales Asiaticas 15, 50-58 (2020).
157	Not relevant	Bloß, N., Loffing, F., Schorer, J. & Büsch, D. Impact of psychological and physical load on the decision-making of top-class handball referees. "International Journal Of Performance Analysis In Sport,[2020#5Year]IF->1.518#1.707" 22, 352-369 (2022).
158	Review	Bloß, N., Schorer, J., Loffing, F. & Büsch, D. Physical load and referees' decision-making in sports games: A scoping review. Journal of Sports Science & Medicine 19, 149-157 (2020).
159	Not relevant	Bloss, N; Schorer, J; Loffing, F; Büsch, D.Decisions and reasonings of top-class handball referees under physical load

S1 File. Studies Identification

160	Not relevant	Boardley, I.D. & Kavussanu, M. Development and validation of the moral disengagement in sport scale. Journal of Sport & Exercise Psychology 29, 608-628 (2007).
161	Not relevant	Bolotin, A. & Bakayev, V. Success criteria of the pedagogical pattern of physical training on self-guide basis with individual assignments among futsal referees. Journal of Human Sport & Exercise 12, 607-615 (2017).
162	Not relevant	Bonney, N., Ball, K., Berry, J. & Larkin, P. Effects of manipulating player numbers on technical and physical performances participating in an Australian football small-sided game. "J Sports Sci,[2020#5Year]IF->2.597#3.060" 38, 2430-2436 (2020).
163	Not relevant	Bonney, N., Berry, J., Ball, K. & Larkin, P. Can match play kicking and physical performance outcomes be replicated in an Australian football small-sided game? Science & Medicine in Football 4, 314-321 (2020).
164	Not relevant	Bonney, N., Berry, J., Ball, K. & Larkin, P. Validity and reliability of an Australian football small-sided game to assess kicking proficiency. "J Sports Sci,[2020#5Year]IF->2.597#3.060" 38, 79-85 (2020).
165	Not relevant	Bordner, S.S. Call ‘Em as they are: What’s Wrong with Blown Calls and What to do about them. "J Phil Sport,[2020#5Year]IF->0.867#0.814" 42, 101-120 (2015).
166	Not relevant	Boullosa, D.A., Abreu, L., Tuimil, J.L. & Leicht, A.S. Impact of a soccer match on the cardiac autonomic control of referees.
167	Not relevant	Bourdas, D.I., Mitrousis, I., Zacharakis, E.D. & Travlos, A.K. Home-audience advantage in basketball: evidence from a natural experiment in EuroLeague games during the 2019-2021 Covid-19 era. Journal of Physical Education & Sport 22, 1553-1563 (2022).
168	Review	Bouzas-Rico, S., De Dios-Álvarez, V., Suárez-Iglesias, D. & Ayán-Pérez, C. Field-based tests for assessing fitness in referees: A systematic review.
169	Not relevant	Bowker, A., et al. Naturalistic Observations of Spectator Behavior at Youth ockey Games. "Sport Psychol,[2020#5Year]IF->1.515#2.080" 23, 301-316 (2009).

S1 File. Studies Identification

170	Not relevant	Bowley, C., Cropley, B., Neil, R., Hanton, S. & Mitchell, I. A life skills development programme for youth football coaches: Programme development and preliminary evaluation. Sport & Exercise Psychology Review 14, 3-22 (2018).
171	Not relevant	Boyko, R., Boyko, A. & Boyko, M. Referee bias contributes to home advantage in English Premiership football. "J Sports Sci,[2020#5Year]IF->2.597#3.060" 25, 1185-1194 (2007).
172	Not physical or physiological variables	Bozdogan et al.The effect of morphological characteristics on the physical and physiological performance of Turkish soccer referees and assistant referees
173	Not relevant	Bozdogan, TK; Kizilet, A; Biçer, B.The effect of morphological characteristics on the physical and physiological performance of Turkish soccer referees and assistant referees
174	Not relevant	Brackenridge, C.H., et al. Measuring the impact of child protection through Activation States. Sport, Education & Society 10, 239-256 (2005).
175	Not relevant	Bradbury, J.C. Monitoring and Employee Shirking: Evidence From MLB Umpires. "Journal Of Sports Economics,[2020#5Year]IF->1.615#1.527" 20, 850-872 (2019).
176	Not relevant	Bradley, P.S. 'Setting the Benchmark' Part 1: The Contextualised Physical Demands of Positional Roles in the FIFA World Cup Qatar 2022. "Biol Sport,[2020#5Year]IF->2.000#2.250" 41, 261-270 (2024).
177	Not relevant	Brady, A.J., Moyna, N.M., Scriney, M. & McCarren, A. Activity profile of elite Gaelic football referees during competitive match play. Science & Medicine in Football 7, 57-63 (2023).
178	Not relevant	Brady, A.J., Moyna, N.M., Scriney, M. & McCarren, A. Competitive level differences in the activity profile of elite Gaelic football referees.
179	Not relevant	Brady, A.J., Scriney, M., Moyna, N.M. & McCarren, A. Identification of movement categories and associated velocity thresholds for elite Gaelic football and hurling referees.

S1 File. Studies Identification

180	Not relevant	Brady, M.S. Suffering in sport: why people willingly embrace negative emotional experiences. "J Phil Sport,[2020#5Year]IF->0.867#0.814" 46, 115-128 (2019).
181	Not relevant	Brandão Kashiwagura, D., Courel-Ibáñez, J., Brandão Kashiwagura, F., Agostinho, M.F. & Franchini, E. Judo technical-tactical dynamics: analysis of attack system effectiveness in high-level athletes. "International Journal Of Performance Analysis In Sport,[2020#5Year]IF->1.518#1.707" 21, 922-933 (2021).
182	Not relevant	Bratuša, Z. THE DECADE OF SERBIAN WATER POLO. Physical Culture / Fizicka Kultura 75, 21-31 (2021).
183	Not relevant	Brightmore, A., et al. Movement and Physiological Demands of Australasian National Rugby League Referees. International Journal of Sports Physiology & Performance 11, 1080-1087 (2016).
184	Not relevant	Brill, C.H. & Brill, H.W. LAVVYERS IN THE BASEBALL HALL OF FAME. Marquette Sports Law Review 34, 137-178 (2023).
185	Not relevant	Brocherie, F., De Larochelambert, Q. & Millet, G.P. Multi-hosting UEFA European Football Championship: fair enough between participating teams? Science & Medicine in Football 7, 171-176 (2023).
186	Not relevant	Brown, D.W. THE RISE AND FALL OF RUGBY FOOTBALL LEAGUE IN NOVA SCOTIA, 1946-1956. Canadian Journal of History of Sport & Physical Education 10, 52-75 (1979).
187	Not relevant	Brown, J. & Sheridan, B.J. The Impact of National Anthem Protests on National Football League Television Ratings. "Journal Of Sports Economics,[2020#5Year]IF->1.615#1.527" 21, 829-847 (2020).
188	Not relevant	Brown, J.C., et al. Non-sanctioning of illegal tackles in South African youth community rugby. "J Sci Med Sport,[2020#5Year]IF->3.607#4.332" 21, 631-634 (2018).
189	Not relevant	Brown, W.M. Paternalism, Drugs, and the Nature of Sports. "J Phil Sport,[2020#5Year]IF->0.867#0.814" 11, 14-22 (1984).

S1 File. Studies Identification

190	Not relevant	Bueno Souza, A.L., et al. Analysis of Lactate, Heart Rate and Rating of Perceived Exertion Responses in Randori, Competition and Specific Evaluation of Judo. Ido Movement for Culture. Journal of Martial Arts Anthropology 22, 39-47 (2022).
191	Not relevant	Burd, H., Deutsch, J. & Waldera, R. Giving Another Purpose to Sport: Teaching Social and Emotional Learning Through the Sociocultural Curriculum Model. JOPERD: The Journal of Physical Education, Recreation & Dance 94, 13-23 (2023).
192	Not relevant	Burillo, A., León-Quismondo, J., Fernández-Luna, Á. & Burillo, P. Why are there no female coaches in elite women's soccer? A qualitative study of Spanish female coaches. "Sport in Society,[2020#5Year]IF->0.939#Not Available" 27, 1-13 (2024).
193	Not relevant	Burke, A.M. Raising the Bar: Increasing Protection for Athletes in the Olympic Movement from Sexual Harassment and Abuse. Journal of Legal Aspects of Sport 31, 60-93 (2021).
194	Not relevant	Burke, L., Loucks, A. & Broad, N. Energy and carbohydrate for training and recovery. "J Sports Sci,[2020#5Year]IF->2.597#3.060" 24, 675-685 (2006).
195	Not relevant	Burke, L.M. Nutrition for the Beautiful Game. International Journal of Sport Nutrition & Exercise Metabolism 16, 332-336 (2006).
196	Not relevant	Burnett, C. KEY FINDINGS OF A NATIONAL STUDY ON SCHOOL SPORT AND PHYSICAL EDUCATION IN SOUTH AFRICAN PUBLIC SCHOOLS. South African Journal for Research in Sport, Physical Education & Recreation 42, 43-60 (2020).
197	Not relevant	Busquets-Ferrer, M., González-Fernández, F.T., Clemente, F.M. & Castillo-Rodriguez, A. Effects of Warm-Up Training on Psychomotor Vigilance and Repeated-Sprint Ability of Professional Soccer Referees: A Pilot Study.
198	Not comparisons in the time phase	Button C, Petersen C. Quantifying the physiological demands of football refereeing with GPS tracking technology

S1 File. Studies Identification

199	Not relevant	Buyrukoğlu, E., Tanir, H., Özdemir, M. & Karagöz, S. Futbol, Voleybol ve Basketbol Branşlarında Aktif Olarak Yer Alan Amatör Sporcuların Algılanan Başarı Motivasyonu ile Ebeveyn Tutumlarının İncelenmesi. Online Journal of Recreation & Sports 13, 172-183 (2024).
200	Not relevant	C, F., et al. Bocha en los Juegos Paralímpicos: La evolución de 1984 a 2016 y perspectivas futuras. Cuadernos de Psicología del Deporte 22, 205-214 (2022).
201	Not comparisons in the time phase	C.Catterall., et al.1993Analysis of the work rates and heart rates of association football referees.
202	Not relevant	Caballero, J.A.R., et al. Physiological profile of national-level Spanish soccer referees.
203	Not relevant	Cabralis, S., Foo, C.E. & Bramble, M.L. Attracting female soccer referees – The Trinidad and Tobago experience. Soccer & Society 24, 799-812 (2023).
204	Not relevant	Calahorro Cañada, F., Torres-Luque, G. & Lara-Sánchez, A.J. La percepción subjetiva de esfuerzo como herramienta válida para la monitorización de la intensidad del esfuerzo en competición de jóvenes futbolistas. Cuadernos de Psicología del Deporte 14, 75-81 (2014).
205	Not English	Calderon Soto, C. & Soler Gutierrez, T. Analysis of locomotor system of Spanish soccer referee.
206	Not relevant	Calmet, M., Sallantin, J., Monino, J.-L. & Lyons, K. Evaluation or Analysis of a Live or a Recorded Video Sequence: An Example from an Analysis of Sports Videos. Ido Movement for Culture. Journal of Martial Arts Anthropology 19, 36-44 (2019).
207	Not relevant	Campbell, P.G., Peake, J.M. & Minett, G.M. The Specificity of Rugby Union Training Sessions in Preparation for Match Demands. International Journal of Sports Physiology & Performance 13, 496-503 (2018).
208	Not relevant	Campo, M. & Louvet, B. Do high emotional intelligent soccer referees better cope with competitive stressors? Movement & Sport Sciences / Science & Motricité, 17-26 (2019).

S1 File. Studies Identification

209	Not relevant	Campos, D. On the Value and Meaning of Football: Recent Philosophical Perspectives in Latin America. "J Phil Sport,[2020#5Year]IF->0.867#0.814" 37, 69-87 (2010).
210	Not relevant	Can, F.G. & Avci, S. Correlation of core stabilization, respiratory functions and injury risk in football referees living in Bolu province, Turkey. Sports Medicine Journal / Medicina Sportiva 17, 3350-3361 (2021).
211	Not relevant	Carboch, J., Praveckova, P. & Kolbinger, O. Do line call errors differ between men's and women's events in tennis? A case study from the 2021 Wimbledon Tournament. "International Journal Of Performance Analysis In Sport,[2020#5Year]IF->1.518#1.707" 23, 155-166 (2023).
212	Not relevant	Carboch, J.A.N. & DušEk, P. Gender differences in squash performance and strategy at the elite level: a notational analysis. Journal of Physical Education & Sport 23, 1010-1015 (2023).
213	Not relevant	Carey, N., Simonton, K.L. & Wallhead, T. Here's an IDEA to Improve Sport Education: Use a Flipped Classroom to Increase Student Role Efficacy. JOPERD: The Journal of Physical Education, Recreation & Dance 94, 37-44 (2023).
214	Not relevant	Carling, C. & Dupont, G. Are declines in physical performance associated with a reduction in skill-related performance during professional soccer match-play? "J Sports Sci,[2020#5Year]IF->2.597#3.060" 29, 63-71 (2011).
215	Not relevant	Carling, C., Wright, C., Nelson, L.J. & Bradley, P.S. Comment on 'Performance analysis in football: A critical review and implications for future research'. "J Sports Sci,[2020#5Year]IF->2.597#3.060" 32, 2-7 (2014).
216	Not high level matches	Carlo Castagna.applicability of a change of direction ability field test in soccer assistant referees
217	Not physical or physiological variables	Carlos David Gómez-Carmona.Kinematic and physiological analysis of the performance of the referee football and its relationship with decision making

S1 File. Studies Identification

218	Not relevant	Carlsson, T., Berglez, J., Koivisto Persson, S. & Carlsson, M. The impact of video review in karate kumite during a Premier League competition. "International Journal Of Performance Analysis In Sport,[2020#5Year]IF->1.518#1.707" 20, 846-856 (2020).
219	Not relevant	Carr, J. & Power, M.J. More than a club, more than a game: the case of Diverse City. Managing Sport & Leisure 25, 99-113 (2020).
220	Not relevant	Carrington, SC; North, JS; Brady, A.Utilising experiential knowledge of elite match officials: Recommendations to improve practice design for football referees
221	Not relevant	Carter-Francique, A.R., Choi, Y., Brooks, D.D., Jamieson, K.M. & Liao, J. Engaging Conversation in Women's Sport and Physical Activity: Traversing Generations. Women in Sport & Physical Activity Journal 30, 109-112 (2022).
222	Not relevant	Casajus, J.A. & Castagna, C. Aerobic fitness and field test performance in elite Spanish soccer referees of different ages.
223	Not relevant	Casajus, J.A., et al. Agreement of body composition methods in elite male football referees. Revista Andaluza de Medicina del Deporte 12, 230-234 (2019).
224	Not relevant	Casal, C.A., Stone, J.A., Iván-Baragaño, I. & Losada, J.L. Effect of goalkeepers' offensive participation on team performance in the women Spanish La Liga: a multinomial logistic regression analysis. "Biol Sport,[2020#5Year]IF->2.000#2.250" 41, 29-39 (2024).
225	Not relevant	Casey, M., Fowlie, J., Charity, M., Harvey, J. & Eime, R. The implications of female sport policy developments for the community-level sport sector: a perspective from Victoria, Australia. International Journal of Sport Policy & Politics 11, 657-678 (2019).
226	Not physical or physiological variables	Castagna, C. & Abt, G. Intermatch variation of match activity in elite Italian soccer referees. "J Strength Cond Res,[2020#5Year]IF->2.973#3.058" 17, 388-392 (2003).

S1 File. Studies Identification

227	Not physical or physiological variables	Castagna, C., & Abt, G.Intermatch variation of match activity in elite Italian soccer referees
228	Not relevant	Castagna, C., Abt, G. & D'Ottavio, S. ACTIVITY PROFILE OF INTERNATIONAL-LEVEL SOCCER REFEREES DURING COMPETITIVE MATCHES. Journal of Strength & Conditioning Research 18, 486-490 (2004).
229	Not relevant	Castagna, C., Abt, G. & D'Ottavio, S. Competitive-level differences in Yo-Yo intermittent recovery and twelve minute run test performance in soccer referees.
230	Review	Castagna, C., Abt, G. & D'Ottavio, S. Physiological aspects of soccer refereeing performance and training.
231	Not relevant	Castagna, C., Abt, G. & D'Ottavio, S. Relation between fitness tests and match performance in elite Italian soccer referees. Journal of Strength & Conditioning Research (Allen Press Publishing Services Inc.) 16, 231-235 (2002).
232	Not relevant	Castagna, C., Abt, G. & D'Ottavio, S. The relationship between selected blood lactate thresholds and match performance in elite soccer referees. "J Strength Cond Res,[2020#5Year]IF->2.973#3.058" 16, 623-627 (2002).
233	Not relevant	Castagna, C., Abt, G., D'Ottavio, S. & Weston, M. Age-related effects on fitness performance in elite-level soccer referees.
234	Not relevant	Castagna, C., Bendiksen, M., Impellizzeri, F.M. & Krustrup, P. Reliability, sensitivity and validity of the assistant referee intermittent endurance test (ARIET) - a modified Yo-Yo IE2 test for elite soccer assistant referees. "J Sports Sci,[2020#5Year]IF->2.597#3.060" 30, 767-775 (2012).
235	Not relevant	Castagna, C., Bizzini, M., Póvoas, S.C.A. & D'Ottavio, S. Timing effect on training-session rating of perceived exertion in top-class soccer referees.
236	Not relevant	Castagna, C., et al. Aerobic Fitness in Top-Class Soccer Referees.

S1 File. Studies Identification

237	Not relevant	Castagna, C., et al. Considerations and best practices for elite football officials return to play after COVID-19 confinement. Managing Sport & Leisure 27, 175-182 (2022).
238	Not relevant	Castagna, C., Impellizzeri, F.M., Bizzini, M., Weston, M. & Manzi, V. Applicability of a change of direction ability field test in soccer assistant referees.
239	Not relevant	Castagna, C., Leguizamon, A.P. & Araujopovoas, S.C. Fitness assessment in talented football referees: an academy based longitudinal field-study.
240	Review	Castagna, C; Abt, G; D'Ottavio, S.Physiological aspects of soccer refereeing performance and training
241	Not relevant	Castagna, C; Abt, G; D'Ottavio, S.Relation between fitness tests and match performance in elite Italian soccer referees
242	Not relevant	Castagna, C; Abt, G; D'Ottavio, S.The relationship between selected blood lactate thresholds and match performance in elite soccer referees
243	Not relevant	Castagna, C; Bizzini, M; Leguizamon, AP; Pizzi, A; Torquati, R; Póvoas, S
244	News	Castagna, C; Bizzini, M; Leguizamon, AP; Pizzi, A; Torquati, R; Póvoas, S.AEROBIC FITNESS IN TOP-CLASS SOCCER REFEREES
245	Not relevant	Castaldi, C.R. Sports mouthguard: its use and misuse in ice hockey.
246	Not relevant	Castaneda, B. & Gray, R. Effects of focus of attention on baseball batting performance in players of differing skill levels. Journal of Sport & Exercise Psychology 29, 60-77 (2007).
247	Not comparisons in the time phase	Castillo D, Weston M, McLaren SJ, Cámara J, Yanci J.Relationships between Internal and External Match-Load Indicators in Soccer Match Officials

S1 File. Studies Identification

248	Not high-level matches	Castillo D.Analysis of the physical and physiological responses of field and assistant soccer referees during Spanish Third Division official matches
249	Not relevant	Castillo, A.V. Review of ITF-Approved Player Analysis Technology (PAT). Coaching & Sport Science Review, 27-35 (2023).
250	book	Castillo, D., Cámara, J. & Yanci, J. Acceleration capacity measured by global positioning system devices of national and provincial soccer referees.
251	Not relevant	Castillo, D., Cámara, J. & Yanci, J. Analysis of the physical and physiological responses of field and assistant soccer referees during Spanish Third Division official matches.
252	book	Castillo, D., Cámara, J. & Yanci, J. Validity of 10 Hz global positioning system devices to measure performance in an incremental cardiovascular field test.
253	Not relevant	Castillo, D., Cámara, J., Castagna, C. & Yanci, J. Effects of the off-Season Period on Field and Assistant Soccer Referees `Physical Performance. "Journal Of Human Kinetics,[2020#5Year]IF->1.664#1.886" 56, 159-166 (2017).
254	Not relevant	Castillo, D., Cámara, J., Castellano, J. & Yanci, J. FOOTBALL MATCH OFFICIALS DO NOT ATTAIN MAXIMAL SPRINTING SPEED DURING MATCHES. "Kinesiology,[2020#5Year]IF->1.225#1.316" 48, 207-212 (2016).
255	Not relevant	Castillo, D., Cámara, J., Lozano, D., Berzosa, C. & Yanci, J. The association between physical performance and match-play activities of field and assistants soccer referees. "Res Sports Med,[2020#5Year]IF->2.554#2.410" 27, 283-297 (2019).
256	Not relevant	Castillo, D., Cámara, J., Sedano, S. & Yanci, J. Impact of official matches on soccer referees' horizontal-jump performance.
257	Not relevant	Castillo, D., Castagna, C., Cámara, J., Iturricastillo, A. & Yanci, J. Influence of team's rank on soccer referees' external and internal match loads during official matches.

S1 File. Studies Identification

258	Not relevant	Castillo, D., et al. Analysis of the success in soccer match officials' decisions during an international tournament according to contextual factors. RICYDE. Revista Internacional de Ciencias del Deporte 15, 225-234 (2019).
259	Not relevant	Castillo, D., et al. Effect of maximum incremental test performance on the vertical jump performance in soccer referees.
260	Not relevant	Castillo, D., Raya-González, J., Scanlan, A.T., Domínguez-Díez, M. & Madueno, M.C. Influence of opponent ranking on the physical demands encountered during Ultimate Frisbee match-play. "Sport Biomech,[2020#5Year]IF->2.023#1.949" 22, 822-833 (2023).
261	Not relevant	Castillo, D., Weston, M., McLaren, S.J., Cámara, J. & Yanci, J. Relationships Between Internal and External Match-Load Indicators in Soccer Match Officials. International Journal of Sports Physiology & Performance 12, 922-927 (2017).
262	Not relevant	Castillo, D., Yanci, J., Cámara, J. & Weston, M. The influence of soccer match play on physiological and physical performance measures in soccer referees and assistant referees. "J Sports Sci,[2020#5Year]IF->2.597#3.060" 34, 557-563 (2016).
263	Not relevant	Castillo, D; Yanci, J; Cámara, J; Weston, M
264	Not relevant	Castillo-Algarra, J., García-Tardón, B. & Pardo, R. Sport in Spanish prisons: Towards the Third Degree or the Third Half? Cultura, Ciencia y Deporte 14, 5-13 (2019).
265	Not relevant	Castillo-Rodríguez, A., Alejo-Moya, E.J., Figueiredo, A., Onetti-Onetti, W. & González-Fernández, F.T. Influence of physical fitness on decision-making of soccer referees throughout the match. Heliyon 9, e19702 (2023).
266	Not relevant	Castillo-Rodríguez, A., Caparrós, J.L.R., Figueiredo, A., González-Fernández, F.T. & Onetti-Onetti, W. Cause-Effect: The Relationship between Role and Experience with Psychological and Physical Responses in the Competition Context in Soccer Referees. J Hum Kinet 89, 289-300 (2023).

S1 File. Studies Identification

267	Not relevant	Castillo-Rodríguez, A., Muñoz-Arjona, C. & Onetti-Onetti, W. National vs. Non-National Soccer Referee: Physiological, Physical, and Psychological Characteristics. Research Quarterly for Exercise & Sport 93, 804-812 (2022).
268	Not relevant	Castillo-Rodríguez, A., Onetti-Onetti, W., Manuel Clemente, F. & Tomás González-Fernández, F. Principal components approach of physiological and physical metrics collected through GPS technology in soccer referees.
269	Not relevant	Castillo-Rodríguez, A., Rodríguez Caparrós, J.L., Figueiredo, A., González-Fernández, F.T. & Onetti-Onetti, W. Cause-Effect: The Relationship between Role and Experience with Psychological and Physical Responses in the Competition Context in Soccer Referees. "Journal Of Human Kinetics,[2020#5Year]IF->1.664#1.886" 89, 1-22 (2023).
270	Not relevant	Catteeuw, P., Gilis, B., Jaspers, A., Wagemans, J. & Helsen, W. Training of Perceptual-Cognitive Skills in Offside Decision Making. Journal of Sport & Exercise Psychology 32, 845-861 (2010).
271	Not relevant	Catteeuw, P., Gilis, B., Wagemans, J. & Helsen, W. Offside decision making of assistant referees in the English Premier League: Impact of physical and perceptual-cognitive factors on match performance. "J Sports Sci,[2020#5Year]IF->2.597#3.060" 28, 471-481 (2010).
272	Not relevant	Catteeuw, P., Helsen, W., Gilis, B. & Wagemans, J. Decision-making skills, role specificity, and deliberate practice in association football refereeing. "J Sports Sci,[2020#5Year]IF->2.597#3.060" 27, 1125-1136 (2009).
273	Not relevant	Catteeuw, P., Helsen, W., Gilis, B., Van Roie, E. & Wagemans, J. Visual Scan Patterns and Decision-Making Skills of Expert Assistant Referees in Offside Situations. Journal of Sport & Exercise Psychology 31, 786-797 (2009).
274	Not relevant	Catteeuw, P; Gilis, B; García-Aranda, JM; Tresaco, F; Wagemans, J; Helsen, W
275	Not relevant	Cazzola, D., Preatoni, E., Stokes, K.A., England, M.E. & Trewartha, G. A modified prebind engagement process reduces biomechanical loading on front row players during scrummaging: a cross-sectional study of 11 elite teams. "Br J Sports Med,[2020#5Year]IF->12.022#10.481" 49, 541-546 (2015).

S1 File. Studies Identification

276	Not relevant	Çelik, A; Gayretli, Z.The influence of soccer match play on physiological and physical performance measures in soccer referees and assistant referees
277	Review	Cerqueira, MS; da Silva, AI; Marins, JCB.Analysis of the FIFA's Model of Physical Evaluation Applied to the Soccer Referees
278	Not relevant	Chagué, F., et al. Smoking and vaping in amateur rugby players, coaches and referees: Findings from a regional survey might help to define prevention targets.
279	Not relevant	Champion, W.T. THE COMMISSIONER GOES TOO FAR: THE BEST INTERESTS OF BASEBALL CLAUSE AND THE ASTROS' "HIGH TECH" SIGN-STEALING SCANDAL. Marquette Sports Law Review 31, 215-242 (2021).
280	Not relevant	Changjing, Z., Gómez, M.-Á. & Lorenzo, A. The evolution of physical and technical performance parameters in the Chinese Soccer Super League. "Biol Sport,[2020#5Year]IF->2.000#2.250" 37, 139-145 (2020).
281	Not relevant	Chen, H.C. & Horne, J. The Covid-19 pandemic and Asian Football Confederation match officials. "Sport in Society,[2020#5Year]IF->0.939#Not Available" 24, 2246-2261 (2021).
282	Conference Paper	Chis, V; Albulescu, I. CONTINUOUS EDUCATION AND TRAINING OF ADULT SPORTSMEN - STUDY CASE: THE FOOTBALL REFEREES
283	Not relevant	Choi, S.M., et al. Operationalizing physical literacy through sport education in a university physical education program. Physical Education & Sport Pedagogy 27, 591-607 (2022).
284	Not relevant	Chopilko, TG; Berezka, SM.HEART BEATS RATE INDICATORS AND STRUCTURE OF ELITE FOOTBALL REFEREES' AND THEIR ASSISTANTS' MOTOR FUNCTIONING IN COMPETITION PERIOD
285	Conference Paper	Chopra, H., Mundody, S. & Reddy Guddeti, R.M. A Key-frame Extraction for Object Detection and Human Action Recognition in Soccer Game Videos.
286	Not relevant	Christiansen, R.C. EDDIE KOTAL: The Experiences that Shaped the First Full-Time NFL Scout. Coffin Corner 43, 13-18 (2021).

S1 File. Studies Identification

287	Not relevant	Chun, S. & Park, S.S. Home Advantage in Skeleton: Familiarity versus Crowd Support. "Journal Of Sports Economics, [2020#5Year]IF->1.615#1.527" 22, 3-26 (2021).
288	Review	Cipriano, P., et al. Investigation on Soccer Referees: A Narrative Review.
289	Not relevant	Cipriano, P., et al. Investigation on Soccer Referees: A Narrative Review. Polish Journal of Sport & Tourism 26, 20-28 (2019).
290	Not relevant	Clark, J.D., Mallett, C.J., Moyle, G.M. & Coulter, T.J. Competitive Situations Requiring Mental Toughness in Women's Australian Rules Football. "J Sports Sci,[2020#5Year]IF->2.597#3.060" 40, 2412-2423 (2022).
291	Not relevant	Clarkson, B.G., Cox, E. & Thelwell, R.C. Negotiating Gender in the English Football Workplace: Composite Vignettes of Women Head Coaches' Experiences. Women in Sport & Physical Activity Journal 27, 73-84 (2019).
292	Not relevant	Coalter, F., Theeboom, M. & Truyens, J. Developing a programme theory for sport and employability programmes for NEETs. International Journal of Sport Policy & Politics 12, 679-697 (2020).
293	Not relevant	Collins, C.L., Fields, S.K. & Comstock, R.D. When the rules of the game are broken: what proportion of high school sports-related injuries are related to illegal activity? Injury Prevention (1353-8047) 14, 34-38 (2008).
294	Not relevant	Collins, H. Applying philosophy to refereeing and umpiring technology.
295	Not relevant	Collins, H. The Philosophy of Umpiring and the Introduction of Decision-Aid Technology. "J Phil Sport,[2020#5Year]IF->0.867#0.814" 37, 135-146 (2010).
296	Not relevant	Colomer, C.M.E., Pyne, D.B., Mooney, M., McKune, A. & Serpell, B.G. A qualitative study exploring tactical performance determinants from the perspective of three Rugby World Cup coaches. International Journal of Sports Science & Coaching 17, 734-741 (2022).

S1 File. Studies Identification

297	Not relevant	Comella, A., Hassett, L., Hunter, K., Cole, J. & Sherrington, C. Sporting opportunities for people with physical disabilities: Mixed methods study of web-based searches and sport provider interviews. "Health Promot J Austr,[2020#5Year]IF->1.476#1.670" 30, 180-188 (2019).
298	Not relevant	Conrad, M. The COVID-19 Pandemic, the Empowering Olympic, Paralympic and Amateur Athletes Act, and the Dawn of a New Age of U.S. Olympic Reform. Journal of Legal Aspects of Sport 31, 1-59 (2021).
299	Not relevant	Constitutions, Institutions, and Games. "J Phil Sport,[2020#5Year]IF->0.867#0.814" 12, 41-51 (1985).
300	Not relevant	Conte, D., et al. Investigating External and Internal Loads in Male Older Adult Basketball Players during Official Games. Journal of Functional Morphology & Kinesiology 7, 111 (2022).
301	Not relevant	Conti, D.J. & McClintock, S.L. Heart Rate Responses of a Head Referee During a Football Game. "Phys Sportsmed,[2020#5Year]IF->1.662#1.971" 11, 108-114 (1983).
302	Not relevant	Cooper, K.H. The History of Aerobics (50 Years and Still Counting). Research Quarterly for Exercise & Sport 89, 129-134 (2018).
303	Not relevant	Correia-Oliveira, C.R. & Andrade-Souza, V.A. Home advantage in soccer after the break due to COVID-19 pandemic: does crowd support matter? International Journal of Sport & Exercise Psychology 20, 1245-1256 (2022).
304	Not relevant	Corrigan, SL; Dwyer, DB; Harvey, B; Gastin, PB.The influence of match characteristics and experience on decision-making performance in AFL umpires
305	Not relevant	Corrion, K., Long, T., Smith, A.L. & d'Arripe-Longueville, F. "It's Not My Fault; It's Not Serious": Athlete Accounts of Moral Disengagement in Competitive Sport. "Sport Psychol,[2020#5Year]IF->1.515#2.080" 23, 388-404 (2009).
306	Not relevant	Corvino, M., Tessitore, A., Minganti, C. & Sibila, M. Effect of Court Dimensions on Players' External and Internal Load during Small-Sided Handball Games. Journal of Sports Science & Medicine 13, 297-303 (2014).

S1 File. Studies Identification

307	Not relevant	Corvino, M., Vuleta, D. & Šibila, M. ANALYSIS OF LOAD AND PLAYERS' EFFORT IN 4vs4 SMALL-SIDED HANDBALL GAMES IN RELATION TO COURT DIMENSIONS. "Kinesiology,[2020#5Year]IF->1.225#1.316" 48, 213-222 (2016).
308	Selected	Costa et al. 2013Monitoring External and Internal Loads of Brazilian Soccer Referees During Official Matches
309	Not relevant	Costa Oliveira, M.A., Dambroz, F., Santos, R. & Moniz, F. VAR implementation and soccer team performance: a comparison between the 2014 and 2018 World Cups. Journal of Physical Education & Sport 21, 3208-3213 (2021).
310	Not relevant	Costa, E.C., et al. Monitoring External and Internal Loads of Brazilian Soccer Referees during Official Matches. Journal of Sports Science & Medicine 12, 559-564 (2013).
311	Not relevant	Costa, EC; Vieira, CMA; Moreira, A; Ugrinowitsch, C; Castagna, C; Aoki, MS
312	Not relevant	Courneya, K.S. & Carron, A.V. Batting First Versus Last: Implications for the Home Advantage. Journal of Sport & Exercise Psychology 12, 312-316 (1990).
313	Not relevant	Coutts, A.J. & Reaburn, P.R. Time and motion analysis of the AFL field umpire. Australian Football League. "J Sci Med Sport,[2020#5Year]IF->3.607#4.332" 3, 132-139 (2000).
314	Not relevant	Cunningham, I., Roche, L. & Mascarenhas, D. Using Mobile 360° Video as a Tool for Enhancing Sport Referee Performance: A Case Study. Case Studies in Sport & Exercise Psychology 7, 43-54 (2023).
315	Not relevant	Cunningham, I., Simmons, P., Mascarenhas, D. & Redhead, S. Skilled Interaction: Concepts of Communication and Player Management in the Development of Sport Officials. International Journal of Sport Communication 7, 166-187 (2014).
316	Not relevant	Curtner-Smith, M.D., Kinchin, G.D., Hastie, P.A., Brunsdon, J.J. & Sinelnikov, O.A. "It's a Lot Less Hassle and a Lot More Fun": Factors That Sustain Teachers' Enthusiasm for and Ability to Deliver Sport Education. "J Teach Phys Educ,[2020#5Year]IF->1.845#2.490" 40, 312-321 (2021).

S1 File. Studies Identification

317	Selected	D. R. D. Mascarenhas et al.2009Physical Performance and Decision Making in Association Football Referees: A Naturalistic Study
318	Not relevant	da Silva, A.I., de los Santos, H. & Cabrera, C. Comparative analysis of body composition of football (Soccer) referees from Brazil and Uruguay.
319	Not relevant	da Silva, A.I., Fernandes, L.C. & Fernandez, R. Energy expenditure and intensity of physical activity in soccer referees during match-play. Journal of Sports Science & Medicine 7, 327-334 (2008).
320	Not relevant	da Silva, A.I., Fernandes, L.C. & Fernandez, R. Time motion analysis of football (soccer) referees during official matches in relation to the type of fluid consumed.
321	Not relevant	Da Silva, A.I., Fernandez, R., Paes, M.R., Fernandes, L.C. & Rech, C.R. Somatotype and body composition of Brazilian football (soccer) referees.
322	Not relevant	da Silva, A.I., Ferreira Junior, A., Spinardi, J., Ortiz da Silva, L. & Soares Rotunno, L. ANÁLISE COMPARATIVA DO ESTADO NUTRICIONAL DE ÁRBITROS E ÁRBITROS ASSISTENTES DE FUTEBOL. Revista Mackenzie de Educacao Fisica e Esporte 14, 78-91 (2015).
323	Not relevant	da Silva, A.I., Paes, M.R. & de Oliveira, M.C. Injuries in Soccer (Football) Referees of Santa Catarina State. Journal of Exercise Physiology Online, 21-30 (2014).
324	Not relevant	da Silva, A.I., Perez, R.F. & Fernandes, L.C. Determining physical capacity and anthropometric profi le of soccer woman referee. Fitness & Performance Journal (Online Edition) 6, 45-52 (2007).
325	Not relevant	da Silva, AI.The influence of match characteristics and experience on decision-making performance in AFL umpires
326	Not relevant	da Silva, AI; Fernandes, LC; Fernandez, R.Energy expenditure and intensity of physical activity in soccer referees during match-play
327	Not relevant	Da Silva, AI; Fernandes, LC; Fernandez, R.Time motion analysis of football (soccer) referees during official matches in relation to the type of fluid consumed

S1 File. Studies Identification

328	Not relevant	da Silva, M.L., et al. Video Assistant Referee in soccer: A Scoping Review. Retos: Nuevas Perspectivas de Educación Física, Deporte y Recreación 50, 1163-1171 (2023).
329	Not relevant	Dağ, A., Özçelik, M.A., Çelebi, F., Aras, D. & Toktaş, N. THE RELATIONSHIP BETWEEN THE MATCH ANALYSIS RESULTS AND RANKING OF SUCCESS IN TURKISH SPOR TOTO FOOTBALL SUPER LEAGUE. Kinesiologia Slovenica 28, 156-168 (2022).
330	Not relevant	Damian, P., Cristian, P. & DragoŞ Florin, T. CONSIDERATIONS REGARDING THE SELECTION IN THE FOOTBALL GAME. Ovidius University Annals, Series Physical Education & Sport/Science, Movement & Health 21, 422-426 (2021).
331	Not high-level matches	Daniel Castillo & Jesús Cámara & Javier Yanci .Analysis of the physical and physiological responses of field and assistant soccer referees during Spanish Third Division official matches
332	Not comparisons in the time phase	Daniel Castillo &Yanci Javier & José A Casajus.Physical Fitness and Physiological Characteristics of Soccer Referees
333	Not relevant	Datcu, R.F., BrÎNdescu, S. & Petracovschi, S. Anxiety and athlete performance: a systematic narrative review of the mutual influence of these concepts. Timisoara Physical Education & Rehabilitation Journal 14, 62-75 (2021).
334	Not relevant	Davey, M.S., et al. Criteria for Return to Play After Hip Arthroscopy in the Treatment of Femoroacetabular Impingement: A Systematic Review. "Am J Sports Med,[2020#5Year]IF->5.810#6.804" 50, 3417-3424 (2022).
335	Not relevant	Davis, P. Game Strengths. "J Phil Sport,[2020#5Year]IF->0.867#0.814" 33, 50-66 (2006).
336	Not relevant	Dayus, J., Callaway, A., Ellis, S. & Butterworth, A. Analysis of playing style across different developmental stages in football. "International Journal Of Performance Analysis In Sport,[2020#5Year]IF->1.518#1.707" 21, 934-952 (2021).
337	Not relevant	de Caldas Honorato, R., et al. Differences in Handgrip Strength-Endurance and Muscle Activation Between Young Male Judo Athletes and Untrained Individuals. Research Quarterly for Exercise & Sport 92, 1-10 (2021).

S1 File. Studies Identification

338	Not relevant	Decarli, A., et al. Anterior cruciate ligament injury in elite football players: Video analysis of 128 cases.
339	Not relevant	Deck, S., Hall, C. & Wilson, P.M. The Impact of Partner Performance on Emotions in Doubles Racquet Sports. Research Quarterly for Exercise & Sport 92, 279-288 (2021).
340	Not relevant	Del Coso, J., et al. Caffeinated Energy Drinks Improve High-Speed Running in Elite Field Hockey Players. International Journal of Sport Nutrition & Exercise Metabolism 26, 26-32 (2016).
341	Not relevant	Del Coso, J., et al. Caffeine-containing energy drink improves physical performance of elite rugby players during a simulated match. Applied Physiology, Nutrition & Metabolism 38, 368-374 (2013).
342	Not relevant	Del Coso, J., et al. Enhancing Physical Performance in Male Volleyball Players With a Caffeine-Containing Energy Drink. International Journal of Sports Physiology & Performance 9, 1013-1018 (2014).
343	Not relevant	Del Lenger, R. DRAKE MAYE'S DAY. Sports Illustrated 134, 96-104 (2023).
344	Not relevant	Delaney, J.A., et al. Acceleration-Based Running Intensities of Professional Rugby League Match Play. International Journal of Sports Physiology & Performance 11, 802-809 (2016).
345	Not relevant	Dellagrana, R.A., Nunes, R.F.H. & Silva, R.L.P. The Importance of Crowd Support and Team Quality to Home Advantage in Brazilian Soccer League First Division. Perceptual & Motor Skills 130, 1255-1268 (2023).
346	Not relevant	Devonport, T.J., et al. 'Nobody needs a label': responses on Facebook to a Team GB equity, diversity and inclusion initiative. "Sport in Society,[2020#5Year]IF->0.939#Not Available" 26, 1113-1132 (2023).
347	Not relevant	Di Domenico, F. & D'Isanto, T. Role of speed and agility in the effectiveness of motor performance. Journal of Physical Education & Sport 19, 1836-1842 (2019).
348	Not relevant	Di Felice, A. & Powell, D. Self-Efficacy of Female Youth Athletes in An Intensive Training Camp. Journal of Sport Behavior 44, 31-50 (2021).

S1 File. Studies Identification

349	Not relevant	Di Giandomenico, A. SOME CONSIDERATION ABOUT SPORT AS INTRINSICALLY ETHICAL ACTIVITY. International Sports Law Review Pandektis 14, 7-19 (2022).
350	Not relevant	Di Salvo, V., Carmont, M.R. & Maffulli, N. Football officials activities during matches: a comparison of activity of referees and linesmen in European, Premiership and Championship matches. Muscles Ligaments Tendons J 1, 106-111 (2011).
351	Not relevant	Díaz Muñoz, R. Estudio Longitudinal de la Condición Física de Árbitros de Élite Españoles. Revista Kronos 15, 1-13 (2016).
352	Not relevant	Díaz-García, J., et al. Coach Encouragement During Soccer Practices Can Influence Players' Mental and Physical Loads. "Journal Of Human Kinetics,[2020#5Year]IF->1.664#1.886" 79, 277-288 (2021).
353	Not relevant	Dicks, M., et al. Coordinated gaze behaviour of handball referees: a practical exploration with focus on the methodical implementation. Movement & Sport Sciences / Science & Motricité, 71-79 (2018).
354	Not relevant	Dilger, A. & Vischer, L. Effects of the Rule Change From Three to Five Substitutions in the Bundesliga. "International Journal Of Sport Finance,[2020#5Year]IF->0.550#1.050" 19, 198-207 (2024).
355	Not relevant	Dix, A. Indications of Referee Bias in Division I Women's College Volleyball: Testing Expectancy Violations and Examining Nonverbal Communication. International Journal of Sport Communication 16, 414-422 (2023).
356	Not relevant	Dixon, N. A Critique of Violent Retaliation in Sport. "J Phil Sport,[2020#5Year]IF->0.867#0.814" 37, 1-10 (2010).
357	Not relevant	Dixon, N. Sport, meritocracy, and praise. "J Phil Sport,[2020#5Year]IF->0.867#0.814" 48, 275-292 (2021).
358	Not relevant	Doari, Y. & Mittleman, J. Basketball as a Complementary Treatment for Physical and Psychosocial Rehabilitation. Palaestra 35, 27-35 (2021).
359	Not relevant	Doewes, R.I. Match fixing in football - the Indonesian experience and response. International Sports Studies 42, 62-68 (2020).

S1 File. Studies Identification

360	Not relevant	Doğusan, S.N. & Koçak, F. Standing on the ice: experiences of women national ice hockey players in Turkey. Physical Culture & Sport. Studies & Research 89, 45-54 (2021).
361	Not high-level matches	Dolański et al.2017Physical activity profile of the referee and the assistant referee during official football matches
362	Not relevant	Dolański, B., Szwarc, A., Heinig, B. & Sitek, M. Physical activity profile of the referee and the assistant referee during official football matches. Baltic Journal of Health & Physical Activity 9, 97-105 (2017).
363	Not relevant	Dolanski, B; Szwarc, A; Heinig, B; Sitek, M.Physical activity profile of the referee and the assistant referee during official football matches
364	Not relevant	Dönmez, G., Babayeva, N., Torğutalp, Ş.Ş. & Özçakar, L. Plantar Fascia Rupture in a Professional Football Referee. Spor Hekimligi Dergisi/Turkish Journal of Sports Medicine 53, 83-88 (2018).
365	Not relevant	Donnan, K., Williams, E.L. & Stanger, N. The Effects of Heat Exposure During Intermittent Exercise on Physical and Cognitive Performance Among Team Sport Athletes. Perceptual & Motor Skills 128, 439-466 (2021).
366	Not relevant	Dorathioto, CA.The perfect penalty
367	Not relevant	Dosseville, F. & Laborde, S. Introduction to the special issue: Officials in sports. Movement & Sport Sciences / Science & Motricité, 3-10 (2015).
368	Not relevant	D'Ottavio, S. & Castagna, C. Physiological load imposed on elite soccer referees during actual match play.
369	Not relevant	D'Ottavio, S; Castagna, C.Analysis of match activities in elite soccer referees during actual match play
370	Not relevant	Dunn, J.G.H. Toward the Combined Use of Nomothetic and Idiographic Methodologies in Sport Psychology: An Empirical Example. "Sport Psychol,[2020#5Year]IF->1.515#2.080" 8, 376-392 (1994).

S1 File. Studies Identification

371	Not relevant	Durdubas, D., Martin, L.J. & Koruc, Z. An examination of nonverbal behaviours in successful and unsuccessful professional volleyball teams. International Journal of Sport & Exercise Psychology 19, 120-133 (2021).
372	Not relevant	Duvinage, C. & Jost, P.-J. The Role of Referees in Professional Sports Contests. "Journal Of Sports Economics,[2020#5Year]IF->1.615#1.527" 20, 1014-1050 (2019).
373	Not relevant	Dymock, A. & Navidi, J. Guide to pre-season. Rugby World, 46-53 (2018).
374	Not relevant	Dymock, A. RUGBY'S NEXT FRONTIERS…. in Rugby World 40-45.
375	Not relevant	Dymock, A., English, T. & Mockford, S. THE SIX NATIONS 2021. Rugby World, 45-57 (2021).
376	Not relevant	Dymock, A., et al. THE 50 MOST INFLUENTIAL PEOPLE IN RUGBY. Rugby World, 33-67 (2020).
377	Not relevant	e Silva, L.D.L., et al. Hemodynamic and motion demands of soccer referees: a comparison between series A and B of the State Championship of Rio de Janeiro, Brazil.
378	Not relevant	Earp, T. The Game Through the Eyes of the Referee. Soccer Journal 57, 20-21 (2012).
379	Not relevant	Ehrlich, S.C. Swimming Against the Current: Mayall v. USA Water Polo and Its Potential Impact on Overseeing Athletic Organizations. Virginia Sports & Entertainment Law Journal 19, 1-28 (2019).
380	Not relevant	Eime, R., Charity, M., Foley, B.C., Fowlie, J. & Reece, L.J. Gender inclusive sporting environments: the proportion of women in non-player roles over recent years. BMC Sports Science, Medicine & Rehabilitation 13, 1-8 (2021).
381	Not relevant	Eliasson, I. Child-rearing in public spaces: the challenging dual-role relationships of parent–coaches and child–athletes of coaches in Swedish team sports. Sport, Education & Society 24, 1006-1018 (2019).

382	Not relevant	English Premier League Soccer at Philadelphia’s Lincoln Financial Field. in SportsField Management (EPG Media & Specialty Information), Vol. 39 18-20 (2023).
383	Not relevant	Engstrom, D. Celebrating Excellence: The Work of Joe Gallo. Teaching Elementary Physical Education 16, 31-34 (2005).
384	Not relevant	Epstein, A. KENTUCKY AND SPORTS LAW. Marquette Sports Law Review 30, 117-168 (2019).
385	Not relevant	Epstein, A. SARAH AND SAM MAKE CONTACT: THOUGHTS ON THE CONTACT SPORTS EXCEPTION AND TITLE IX AT 50. Marquette Sports Law Review 33, 173-193 (2022).
386	Not relevant	EraİL, S. & Uzun, R.N. Comparison of Self-Efficacy of Individual and Team Athletes. Mediterranean Journal of Sport Science (MJSS) 6, 584-592 (2023).
387	Not relevant	Ermidis, G., Randers, M.B., Krustrup, P. & Mohr, M. Technical demands across playing positions of the Asian Cup in male football. "International Journal Of Performance Analysis In Sport,[2020#5Year]IF->1.518#1.707" 19, 530-542 (2019).
388	Not relevant	Errekagorri, I., Castellano, J., Echeazarra, I. & Lago-Peñas, C. The effects of the Video Assistant Referee system (VAR) on the playing time, technical-tactical and physical performance in elite soccer. "International Journal Of Performance Analysis In Sport,[2020#5Year]IF->1.518#1.707" 20, 808-817 (2020).
389	Not relevant	Errekagorri, I., Castellano, J., Echeazarra, I., López-Del Campo, R. & Resta, R. A longitudinal analysis of technical-tactical and physical performance of the teams in the Spanish LaLiga Santander: An eight-season study. "Biol Sport,[2020#5Year]IF->2.000#2.250" 39, 389-396 (2022).
390	Not relevant	Errekagorri, I; Castellano, J; Echeazarra, I; Lago-Peñas, C.The effects of the Video Assistant Referee system (VAR) on the playing time, technical-tactical and physical performance in elite soccer

391	Not relevant	Esfahani, D.N. & Rahbari, S. Identifying the Factors Affecting the Establishment of Knowledge-Based Sports Companies Based On Electronic Knowledge: A Qualitative Analysis. Journal of New Studies in Sport Management 3, 530-543 (2022).
392	Not relevant	Evans, S.D., et al. The physical demands of Super League rugby: Experiences of a newly promoted franchise. "Eur J Sport Sci,[2020#5Year]IF->2.781#3.228" 15, 505-513 (2015).
393	Not physical or physiological variables	Fabrice Dosseville. 2011.Refereeing decisions in soccer and physical performances in ecological situation
394	Not relevant	Fabrice, D., Sylvain, L., Alan, T. & Edoh, K.P. Refereeing decisions in soccer and physical performances in ecological situation.
395	Not relevant	Fader, N., Legg, E. & Ross, A. Finding a Sense of Community in Youth Soccer: A Composite Vignette of the Refugee Experience. Journal of Park & Recreation Administration 38, 97-115 (2020).
396	Not relevant	Fairley, S. & Tyler, B.D. Bringing Baseball to the Big Screen: Building Sense of Community Outside of the Ballpark. "J Sport Manage,[2020#5Year]IF->2.359#2.877" 26, 258-270 (2012).
397	Not relevant	Fairness, Epistemology, and Rules: A Prolegomenon to a Philosophy of Officiating? "J Phil Sport,[2020#5Year]IF->0.867#0.814" 38, 229-253 (2011).
398	Not relevant	Faltings, R., Krumer, A. & Lechner, M. Rot-Jaune-Verde: On linguistic bias of referees in Swiss soccer*.
399	Not relevant	Feddermann-Demont, N., et al. Recommendations for initial examination, differential diagnosis, and management of concussion and other head injuries in high-level football. Scandinavian Journal of Medicine & Science in Sports 30, 1846-1858 (2020).
400	Not relevant	Fenemor, S.P., et al. Practical application of a mixed active and passive heat acclimation protocol in elite male Olympic team sport athletes. Applied Physiology, Nutrition & Metabolism 47, 981-991 (2022).

S1 File. Studies Identification

401	Not relevant	Fenner, J.S.J., Iga, J. & Unnithan, V. The evaluation of small-sided games as a talent identification tool in highly trained prepubertal soccer players. "J Sports Sci,[2020#5Year]IF->2.597#3.060" 34, 1983-1990 (2016).
402	Not relevant	Ferioli, D., et al. Combined Effect of Number of Players and Dribbling on Game-Based-Drill Demands in Basketball. International Journal of Sports Physiology & Performance 15, 825-832 (2020).
403	Not relevant	Ferland, P.-M., Laurier, A. & Comtois, A.S. Relationships Between Anthropometry and Maximal Strength in Male Classic Powerlifters. International Journal of Exercise Science 13, 1512-1531 (2020).
404	Selected	Fernandes da Silva et al.2022Match activity profile and heart rate responses of top-level soccer referees during Brazilian national first and second division and regional championships
405	Not relevant	Fernandes da Silva, J., et al. Match activity profile and heart rate responses of top-level soccer referees during Brazilian national first and second division and regional championships. Science & Medicine in Football 7, 263-271 (2023).
406	Not comparisons in the time phase	Fernandez and Building, 2008.Energy expenditure and intensity of physical activity in soccer referees during match-play
407	Not relevant	Fernández Vargas, G.E., Inácio da Silva, A. & Arruda, M. Anthropometric profile and physical fitness of the professional referees Chilean soccer.
408	Not high-level matches	Fernández-Elías, Valentín & Gómez López, Maite & De la Vega, Ricardo & Clemente-Suárez, Vicente.Physical demands, heart rate response and performance of talent soccer referees
409	Not relevant	Ferrari, W.R., Sarmento, H. & Vaz, V. Match Analysis in Handball: A Systematic Review. Montenegrin Journal of Sports Science & Medicine 8, 63-76 (2019).
410	Not relevant	Ferreira Meneguite, Y.N., Bernardes Leite, L., Correia Da Silva, D., Gomes De Moura, A. & Neiva Lavorato, V. Influence of the video assistant referee (VAR) on the Brazilian Men's Soccer Championship. Journal of Physical Education & Sport 22, 858-862 (2022).

S1 File. Studies Identification

411	Not relevant	Fetisova, Y., Zois, J., Spittle, M. & Dawson, A. Coaches' internal model of the tennis serve technique: Knowing or understanding? International Journal of Sports Science & Coaching 16, 568-584 (2021).
412	Not relevant	Filip-Stachnik, A., et al. Acute Effects of Caffeinated Chewing Gum on Volleyball Performance in High-Performance Female Players. "Journal Of Human Kinetics,[2020#5Year]IF->1.664#1.886" 84, 92-102 (2022).
413	Not relevant	Fillon, A., et al. 2022 French Report Card on Physical Activity and Sedentary Behaviors in Children and Youth: From Continuous Alarming Conclusions to Encouraging Initiatives. Journal of Physical Activity & Health 20, 664-673 (2023).
414	Not relevant	Foster, C., Cortis, C. & Fusco, A. Editorial: Exercise Evaluation and Prescription—Second Edition. Journal of Functional Morphology & Kinesiology 8, 1-6 (2023).
415	Not relevant	Fostiak, M., Frołowicz, T., Pogorzelska, M. & Klonowska, J. Athletics training programme for children studying in sports schools in Poland. Baltic Journal of Health & Physical Activity 11, 134-148 (2019).
416	Not relevant	Fowler, B., Smith, J., Nordstrom, H. & Ferguson, T. Ice hockey officiating retention: a qualitative understanding of junior ice hockey officials' motivations in Canada. Managing Sport & Leisure 24, 18-31 (2019).
417	Not relevant	Frank, S. Laudrup or Ibrahimović: who is the best Scandinavian soccer player of all time? Soccer & Society 19, 704-715 (2018).
418	Not relevant	Fritsch, J., Redlich, D., Latinjak, A. & Hatzigeorgiadis, A. The behavioural component of emotions: exploring outward emotional reactions in table tennis. International Journal of Sport & Exercise Psychology 20, 397-415 (2022).
419	Not relevant	Fry, J.P. 34 Sports and "The Fragility of Goodness"[1]. "J Phil Sport,[2020#5Year]IF->0.867#0.814" 31, 34-46 (2004).
420	Not relevant	Full Issue PDF, Volume 85, Supplement 1. Research Quarterly for Exercise & Sport 85, A-i-A-168 (2014).

421	Not relevant	Fullagar, H.H.K., Delaney, J., Duffield, R. & Murray, A. Factors influencing home advantage in American collegiate football. Science & Medicine in Football 3, 163-168 (2019).
422	Not relevant	Fullagar, S., Rich, E., Pavlidis, A. & van Ingen, C. Feminist Knowledges as Interventions in Physical Cultures. "Leisure Sci,[2020#5Year]IF->1.952#2.232" 41, 1-16 (2019).
423	Not relevant	Fuller, A. Soccer and the city: the game and its fans in Solo and Yogyakarta. "Sport in Society,[2020#5Year]IF->0.939#Not Available" 20, 675-688 (2017).
424	Not relevant	Fuller, C.W., et al. Injury risks associated with tackling in rugby union.
425	Not relevant	Furley, P. & Schweizer, G. Nonverbal Communication of Confidence in Soccer Referees: An Experimental Test of Darwin's Leakage Hypothesis. Journal of Sport & Exercise Psychology 38, 590-597 (2016).
426	Not relevant	Furtado Mesa, M., Stout, J.R., Redd, M.J. & Fukuda, D.H. Accumulated Workload Differences in Collegiate Women's Soccer: Starters versus Substitutes. Journal of Functional Morphology & Kinesiology 8, 78 (2023).
427	Not relevant	Gabbett, T. Influence of playing standard on the physical demands of professional rugby league. "J Sports Sci,[2020#5Year]IF->2.597#3.060" 31, 1125-1138 (2013).
428	Not relevant	Gabbett, T.J. & Hulin, B.T. Activity and recovery cycles and skill involvements of successful and unsuccessful elite rugby league teams: A longitudinal analysis of evolutionary changes in National Rugby League match-play. "J Sports Sci,[2020#5Year]IF->2.597#3.060" 36, 180-190 (2018).
429	Not relevant	Gabbett, T.J. Activity cycles of national rugby league and national youth competition matches.
430	Not relevant	Gabbett, TJ.Activity and Recovery Cycles of National Rugby League Matches Involving Higher and Lower Ranked Teams
431	Not relevant	Galanti, G; Pizzi, A; Lucarelli, M; Stefani, L; Gianassi, M; Di Tante, V; Toncelli, L; Moretti, A; Del Furia, F

S1 File. Studies Identification

432	Not relevant	Gallivan, P. Four Longhorns Help Upstart Jets Win Super Bowl III. Coffin Corner 45, 12-17 (2023).
433	Not relevant	Gallo-Salazar, C., et al. Enhancing Physical Performance in Elite Junior Tennis Players With a Caffeinated Energy Drink. International Journal of Sports Physiology & Performance 10, 305-310 (2015).
434	Not relevant	Galy, O., et al. RELATIONSHIPS BETWEEN HEART RATE AND PHYSIOLOGICAL PARAMETERS OF PERFORMANCE IN TOP-LEVEL WATER POLO PLAYERS. "Biol Sport,[2020#5Year]IF->2.000#2.250" 31, 33-38 (2014).
435	Not relevant	Gamache, R. Contextualizing Replay: Remediation, Affective Economies, Ontological Authority, and the Facade of Certitude. "Sociol Sport J,[2020#5Year]IF->2.635#2.272" 37, 236-245 (2020).
436	Not relevant	Gamble, D., Bradley, J., McCarren, A. & Moyna, N.M. Team performance indicators which differentiate between winning and losing in elite Gaelic football. "International Journal Of Performance Analysis In Sport,[2020#5Year]IF->1.518#1.707" 19, 478-490 (2019).
437	Not relevant	Gamonales, J.M., Muñoz-Jiménez, J., León, K. & Ibáñez, S.J. EFFECTIVENESS OF THE LAUNCH AT FA5 FOR BLIND PERSONS IN 2016 PARALYMPIC GAMES. International Journal of Medicine & Science of Physical Activity & Sport / Revista Internacional de Medicina y Ciencias de la Actividad Física y del Deporte 19, 745-764 (2019).
438	Not relevant	Gaoua, N., de Oliveira, R.F. & Hunter, S. Perception, Action, and Cognition of Football Referees in Extreme Temperatures: Impact on Decision Performance. "Front Psychol,[2020#5Year]IF->2.067#2.723" 8, 1479 (2017).
439	Review	Gaoua, N; de Oliveira, RF; Hunter, S.Perception, Action, and Cognition of Football Referees in Extreme Temperatures: Impact on Decision Performance
440	Not relevant	Garcia, C.A. & Anta, R.C. New proposal for the physical fitness evaluation in football referees.
441	Not relevant	García-Aliaga, A., et al. A Longitudinal Study on the Evolution of the Four Main Football Leagues Using Artificial Intelligence: Analysis of the Differences in English Premier League Teams. Research Quarterly for Exercise & Sport 94, 529-537 (2023).

S1 File. Studies Identification

442	Not relevant	García-Aliaga, A., et al. Effect of Increasing the Number of Substitutions on Physical Performance during Periods of Congested Fixtures in Football. Sports (2075-4663) 11, 25 (2023).
443	Not relevant	García-Santos, D., Gómez-Ruano, M.A., Vaquera, A. & Ibáñez, S.J. Systematic review of basketball referees' performances. "International Journal Of Performance Analysis In Sport,[2020#5Year]IF->1.518#1.707" 20, 495-533 (2020).
444	Review	García-Santos, D; Gómez-Ruano, MA; Vaquera, A; Ibáñez, SJ.Systematic review of basketball referees' performances
445	Not relevant	García-Santos, D; Pino-Ortega, J; García-Rubio, J; Vaquera, A; Ibáñez, SJ
446	Not relevant	Gardner, R. On Performance-Enhancing Substances and the Unfair Advantage Argument. "J Phil Sport,[2020#5Year]IF->0.867#0.814" 16, 59-73 (1989).
447	Not relevant	Gazzo, F., Giráldez, J., Villaseca-Vicuña, R., González-Jurado, J.A. & Zabaloy, S. Acute Effects on Physical Performance Measures after 45 Min of Official Competition in Youth Soccer Players. Journal of Functional Morphology & Kinesiology 6, 1-10 (2021).
448	Not relevant	Gee, B.L. & Leberman, S.I. Sports Media Decision Making in France: How They Choose What We Get to See and Read. International Journal of Sport Communication 4, 321-343 (2011).
449	Not relevant	Gelade, G.A. The Influence of Team Composition on Attacking and Defending in Football. "Journal Of Sports Economics,[2020#5Year]IF->1.615#1.527" 19, 1174-1190 (2018).
450	Not relevant	George-Sebastian, I. & Alexandru, C. The Role of Proprioceptive Training as a Method of Functional Rehabilitation of Ankle Sprains in Futsal Players. Sport & Society / Sport si Societate 19, 20-26 (2019).
451	Not relevant	Ghahfarokhi, E.A., Soroush, S. & Hasanbeigi, H. INVESTIGATING THE HOME ADVANTAGE IN THE WORLD'S PRESTIGIOUS FOOTBALL LEAGUES BEFORE AND AFTER THE OUTBREAK OF COVID-19. Revista Brasileira de Futsal e Futebol 14, 119-129 (2022).

S1 File. Studies Identification

452	Conference Paper	Ghasemi, A., Momeni, M., Rezaee, M. & Gholami, A. The difference in visual skills between expert versus novice soccer referees.
453	Not relevant	Gianturco, L., et al. Foot and soccer referees': A pilot study searching "Performance" throughout prevention.
454	Not relevant	Gianturco, L., et al. Left ventricular longitudinal strain in soccer referees.
455	Not relevant	Giel, T. & Breuer, C. The determinants of the intention to continue voluntary football refereeing. "Sport Management Review,[2020#5Year]IF->3.337#3.761" 23, 242-255 (2020).
456	Not relevant	Giel, T., Dallmeyer, S., Memmert, D. & Breuer, C. Corruption and Self-Sabotage in Sporting Competitions – An Experimental Approach to Match-Fixing Behavior and the Influence of Deterrence Factors. "Journal Of Sports Economics,[2020#5Year]IF->1.615#1.527" 24, 497-525 (2023).
457	Not relevant	Gierczuk, D., Bujak, Z. & Cieśliński, I. Effects of Led Lighting Training on Response Time in Greco-Roman Wrestlers. Polish Journal of Sport & Tourism 30, 11-16 (2023).
458	Not relevant	Gierczuk, D., Lyakh, V., Sadowski, J. & Bujak, Z. Speed of Reaction and Fighting Effectiveness in Elite Greco-Roman Wrestlers. Perceptual & Motor Skills 124, 201-213 (2017).
459	Not relevant	Gleaves, J. & Christiansen, A.V. Athletes' perspectives on WADA and the code: a review and analysis. International Journal of Sport Policy & Politics 11, 341-353 (2019).
460	Not relevant	Gmeiner, M.W. History-Dependent Mixed Strategies: Evidence From Major League Baseball. "Journal Of Sports Economics,[2020#5Year]IF->1.615#1.527" 20, 371-398 (2019).
461	Not relevant	Gökçe, U., Dalboy, A., An, N., Turp, M.T. & Kurnaz, M.L. POTENTIAL RISKS OF OUTDOOR SPORTS IN A CHANGING CLIMATE. SPORMETRE: The Journal of Physical Education & Sport Sciences / Beden Eğitimi ve Spor Bilimleri Dergisi 20, 145-163 (2022).

S1 File. Studies Identification

462	Not relevant	Golden, N. The Immaculate Reception That Nearly Wasn't. Coffin Corner 45, 5-11 (2023).
463	Not relevant	Goldman, D.E., Turnnidge, J., Kelly, A.L., deVos, J. & Côté, J. Athlete perceptions of playing-up in youth soccer. "J Appl Sport Psychol,[2020#5Year]IF->2.150#2.420" 34, 862-885 (2022).
464	Not relevant	Gollan, S., Bellenger, C. & Norton, K. Contextual Factors Impact Styles of Play in the English Premier League. Journal of Sports Science & Medicine 19, 78-83 (2020).
465	Not relevant	Gomes, R; Mendes, R; Ferreira, A; Mendes, R; Dias, G; Martins, F.Physical and Physiological Demands of Amateur Portuguese Field and Assistant Football Referees
466	Not relevant	Gomez-Carmona, C. & Pino-Ortega, J. Kinematic and physiological analysis of the performance of the referee football and its relationship with decision making. Journal of Human Sport & Exercise 11, 397-414 (2016).
467	Not relevant	Gómez-Carmona, C.D., Mancha-Triguero, D., Pino-Ortega, J. & Ibáñez, S.J. Characterization and sex-related differences in the multi-location external workload profile of semiprofessional basketball players. A cross-sectional study. "Eur J Sport Sci,[2020#5Year]IF->2.781#3.228" 22, 1816-1826 (2022).
468	Not relevant	Gómez-Carmona, C.D., Mancha-Triguero, D., Pino-Ortega, J. & Ibáñez, S.J. Exploring Physical Fitness Profile of Male and Female Semiprofessional Basketball Players through Principal Component Analysis-A Case Study. Journal of Functional Morphology & Kinesiology 6, 1-12 (2021).
469	Not relevant	Gonçalves, B., et al. Can tracking data help in assessing interpersonal contact exposure in team sports during the COVID-19 pandemic?
470	Not relevant	Gong, B., et al. Impact of technical and physical key performance indicators on ball possession in the Chinese Super League. "International Journal Of Performance Analysis In Sport,[2020#5Year]IF->1.518#1.707" 21, 909-921 (2021).
471	Not relevant	González Ortega, J., López Hernández, R. & Sánchez Puche, E. Anthropometric profile and physical fitness of soccer referees from La Guajira, Colombia. Cultura, Ciencia y Deporte 18, 77-92 (2023).

S1 File. Studies Identification

472	Not relevant	González Rodenas, J., et al. Past, present and future of goal scoring analysis in professional soccer. Retos: Nuevas Perspectivas de Educación Física, Deporte y Recreación 37, 774-785 (2020).
473	Not relevant	Gonzalez-Artetxe, A., Folgado, H., Pino-Ortega, J., Rico-González, M. & Arcos, A.L. Effects of free play or artificial rules on young soccer players' individual tactical behaviour: a one-by-one analysis. "Biol Sport,[2020#5Year]IF->2.000#2.250" 40, 1069-1078 (2023).
474	Not relevant	Gonzalez-Artetxe, A., Pino-Ortega, J., Rico-González, M. & Los Arcos, A. Training effects of artificial rules on youth soccer team tactical behavior. Physical Education & Sport Pedagogy 27, 467-482 (2022).
475	Not relevant	González-Ortega, J., López-Hernández, R. & Sánchez-Puche, E. Anthropometric profile and physical fitness of soccer referees from La Guajira, Colombia.
476	Not relevant	Gottlieb, R., Aviv, E., Shalom, A., Gonzalez, J.C. & Meckel, Y. The flag effect on the physical performance of soccer assistant referees.
477	Not relevant	Gould, D., Pierce, S. & Wright, E. An autoethnographic account and interpretation of sport related life skills and personal development. International Journal of Sport & Exercise Psychology 21, 175-195 (2023).
478	Not relevant	Graczyk, J.P., Dierdorff, E.C., Rubin, R.S. & Lemmon, G. Exploring Individual Antecedents of Performance Error: False Starts in Collegiate Football. "Hum Perform,[2020#5Year]IF->1.172#2.122" 34, 217-238 (2021).
479	Not relevant	Graham, J. BODY TALK. Rugby World, 66-73 (2023).
480	Review	Gray, A.J. & Jenkins, D.G. Match analysis and the physiological demands of australian football.
481	Not relevant	Green, B., et al. Calf muscle strain injuries in elite Australian Football players: A descriptive epidemiological evaluation. Scandinavian Journal of Medicine & Science in Sports 30, 174-184 (2020).

S1 File. Studies Identification

482	Not relevant	Greenlees, I., Buscombe, R., Thelwell, R., Holder, T. & Rimnier, M. Impact of Opponents' Clothing and Body Language on Impression Formation and Outcome Expectations. Journal of Sport & Exercise Psychology 27, 39 (2005).
483	Not relevant	Grehaigne, J.-F. & Godbout, P. Tactical Knowledge in Team Sports From a Constructivist and Cognitivist Perspective. Quest (00336297) 47, 490-505 (1995).
484	Conference Paper	Grigore, V; Courteix, D.Differentiation of Perceptual Processes in Elite and Assistant Soccer Referees
485	Not relevant	Grijalbo, C., Martínez-Gallego, R. & Guzmán, J.F. Verbal coping of coaches in competition: Differences depending on emotional intelligence and self-determined motivation. International Journal of Sports Science & Coaching 17, 264-273 (2022).
486	Not relevant	Guedes Da Maia, G.S., et al. Relationship between the occurrence of cards, playing position and situational variables in starter and non-starter players in the Brazilian soccer first-division championship. Human Movement 24, 62-71 (2023).
487	Not relevant	Guérandel, C. Being Involved in Sports or Giving Up: The Effects of Context on Teenage Girls' Practice in French Disadvantaged Urban Neighborhoods. "Sociol Sport J,[2020#5Year]IF->2.635#2.272" 39, 362-372 (2022).
488	Not relevant	Guérandel, C. Being Involved in Sports or Giving Up: The Effects of Context on Teenage Girls' Practice in French Disadvantaged Urban Neighborhoods. "Sociol Sport J,[2020#5Year]IF->2.635#2.272" 39, 362-372 (2022).
489	Not relevant	Guermazi, M., Ghroubi, S., Elleuch, M.H. & Rodineau, J. Endofibrose de l'artere iliaque externe chez un arbitre de football. Journal de Traumatologie du Sport 20, 174-178 (2003).
490	Not relevant	Guijarro, E., MacPhail, A., Arias-Palencia, N.M. & González-Víllora, S. Exploring Game Performance and Game Involvement: Effects of a Sport Education Season and a Combined Sport Education—Teaching Games for Understanding Unit. "J Teach Phys Educ,[2020#5Year]IF->1.845#2.490" 41, 411-424 (2022).

S1 File. Studies Identification

491	Not relevant	Guimarães, K.L., Barreira, J. & Galatti, L.R. "BEING A WOMAN IN A FOOTBALL COURSE IS ALREADY A DISADVANTAGE": EXPERIENCES OF FEMALE COACHES IN CBF ACADEMY COURSES. Movimento (0104754X) 29, 01-16 (2023).
492	Not relevant	Gündüz, N. & Yanar, N. Self assessment in physical education: Investigation of teacher and student opinions. International Journal of Physical Education 58, 21-39 (2021).
493	Not relevant	Guo, M. & Fu, S. Running With a Mask? The Effect of Air Pollution on Marathon Runners' Performance. "Journal Of Sports Economics,[2020#5Year]IF->1.615#1.527" 20, 903-928 (2019).
494	Not relevant	Hafeez, A., Hafeez, U., Amin, A. & Hasan, S. VAR technology in English football; Implications of intervening in a fast-moving game. International Sports Studies, 80-95 (2022).
495	Not physical or physiological variables	Hai Tan.Research on the physicalability training and nutritional supplement of the highest level Chinese national grade soccer referees
496	Not relevant	Hairui, L., Wei, S. & Hastie, P.A. Responses of Chinese university students and their teacher to a season of Sport Education. International Sports Studies 42, 5-20 (2020).
497	Not relevant	Hardman, A. & Fox, L. On Sportsmanship and "Running Up the Score": Issues of Incompetence and Humiliation. "J Phil Sport,[2020#5Year]IF->0.867#0.814" 23, 58-69 (1996).
498	Not relevant	Harper, L.D., Field, A., Corr, L.D. & Naughton, R.J. The Physiological, Physical, and Biomechanical Demands of Walking Football: Implications for Exercise Prescription and Future Research in Older Adults. Journal of Aging & Physical Activity 28, 478-488 (2020).
499	Not relevant	Harvey, B.N. ENERGY SYSTEM DEVELOPMENT IN ELITE MIXED MARTIAL ARTS. Journal of Australian Strength & Conditioning 26, 75-89 (2018).

S1 File. Studies Identification

500	Not relevant	Haryanto, J., Becerra-PatiÑO, B. & Padli. Exploring the impact of eye-hand coordination on backhand drive stroke mastery in table tennis regarding gender, height, and weight of athletes. Journal of Physical Education & Sport 23, 2710-2717 (2023).
501	No full-text	Hassan, A.M., Ali, A.H. & Alshawi, H.N.H. The relationship of refereeing performance of the leading personality and thyroxin hormone level for football referees (First division).
502	Not relevant	Hastie, P.A. & Trost, S.G. Student Physical Activity Levels During a Season of Sport Education. "Pediatr Exerc Sci,[2020#5Year]IF->1.489#1.991" 14, 64 (2002).
503	Not relevant	Haugen, T., Hopkins, W., Breitschädel, F., Paulsen, G. & Solberg, P. Fitness Tests and Match Performance in a Male Ice Hockey National League. International Journal of Sports Physiology & Performance 16, 1303-1310 (2021).
504	Not relevant	Haynes, J. The Protection of Sports Broadcast Rights in the Commonwealth Caribbean after TVJ v CVM. Entertainment & Sports Law Journal 19, 1-19 (2021).
505	Selected	Helsen & Bultynck. 2004Physical and perceptual-cognitive demands of top-class refereeing in association football
506	Not relevant	Helsen, W. & Bultynck, J.B. Physical and perceptual-cognitive demands of top-class refereeing in association football. "J Sports Sci, [2020#5Year]IF->2.597#3.060" 22, 179-189 (2004).
507	Not relevant	Helsen, W.F., Spitz, J. & Ziv, G. The acquisition of perceptual-cognitive expertise in officiating in association football -- state of the art. Asian Journal of Sport & Exercise Psychology 3, 39-46 (2023).
508	Not relevant	Helsen, W; Bultynck, JB.Physical and perceptual-cognitive demands of top-class refereeing in association football
509	Not relevant	Hemelryck, W., et al. ULTRASONOGRAPHIC ASSESSMENT OF NECK MUSCULAR SIZE AND RANGE OF MOTION IN RUGBY PLAYERS. International Journal of Sports Physical Therapy 13, 28-38 (2018).

S1 File. Studies Identification

510	Not relevant	Hemphill, D. Deeper Inside the Beautiful Game. "J Phil Sport,[2020#5Year]IF->0.867#0.814" 32, 105 (2005).
511	Not relevant	Henderson, C.W. Two balls is too many: stadium performance and queerness among Portland’s Rose City Riveters supporters club. "Sport in Society, [2020#5Year]IF->0.939#Not Available" 21, 1031-1046 (2018).
512	Not relevant	Henderson, M.J., Harries, S.K., Poulos, N., Fransen, J. & Coutts, A.J. RUGBY SEVENS MATCH DEMANDS AND MEASUREMENT OF PERFORMANCE: A REVIEW. "Kinesiology, [2020#5Year]IF->1.225#1.316" 50, 49-59 (2018).
513	Not relevant	Henley-Martin, S.R., Hiscock, D.J., Ducker, K.J., Jacques, A. & Brade, C.J. Performance, perceptual and physiological comparison of traditional and small-sided games in youth hockey. International Journal of Sports Science & Coaching 16, 749-755 (2021).
514	Not relevant	Henríquez, M., et al. Assessing the Match Physical Responses of International Referees for Footballers with Cerebral Palsy: A Tournaments and Halves Comparative Analysis.
515	Not relevant	Henshon, M. Baseball's Short Summer and Coming Strike. Entertainment & Sports Lawyer 36, 18-24 (2020).
516	Not relevant	Herman, D.C., et al. Previous High School Participation in Varsity Sport and Jump-Landing Biomechanics in Adult Recreational Athletes. Journal of Athletic Training (Allen Press) 54, 1089-1094 (2019).
517	Not relevant	Heyn, J. & Fleckenstein, J. Incidence of injury and pain in referees in German national handball leagues: a cohort study. BMC Sports Science, Medicine & Rehabilitation 13, 1-7 (2021).
518	Not relevant	Hianik, J., ŠImonek, J. & HoriČKa, P. Impact of a specialized conditioning program on the development of postural stability of soccer referees. Journal of Physical Education & Sport 17, 135-141 (2017).
519	Not relevant	Higham, A.J., Newman, J.A., Stone, J.A. & Rumbold, J.L. Coaches' Experiences of Morality in English Professional Football Environments: Recommendations for Creating a Moral Atmosphere. International Sport Coaching Journal 9, 211-221 (2022).

S1 File. Studies Identification

520	Not relevant	Higham, J.E.S. & Hinch, T.D. Sport, Space, and Time: Effects of the Otago Highlanders Franchise on Tourism. "J Sport Manage, [2020#5Year]IF->2.359#2.877" 17, 235 (2003).
521	Not relevant	Hill, K., Bosch, Z.M., Tortorici, C.A. & Trevino, A.P. 2015 & 2016 ANNUAL SURVEYS: RECENT DEVELOPMENTS IN SPORTS LAW. Marquette Sports Law Review 27, 543-613 (2017).
522	Not relevant	Hills, S., Walker, M. & Dixon, M. The Importance of Theorizing Social Change in Sport for Development: A Case Study of Magic Bus in London. "J Sport Manage, [2020#5Year]IF->2.359#2.877" 33, 415-425 (2019).
523	Not relevant	Hilmer, C. & Hilmer, M.J. Does confirmation bias exist in judged events at the Olympic Games? Journal of Quantitative Analysis in Sports 17, 1-10 (2021).
524	Not relevant	Hoare, D.G. & Warr, C.R. Talent identification and women's soccer: An Australian experience. "J Sports Sci, [2020#5Year]IF->2.597#3.060" 18, 751-758 (2000).
525	Not relevant	Hodges, J.A. THE IMPORTANCE OF QUANTIFYING BALL IN PLAY TIME IN TEAM SPORTS. Journal of Australian Strength & Conditioning 29, 70-77 (2021).
526	Not relevant	Hoffman, J.R. The Applied Physiology of American Football. International Journal of Sports Physiology & Performance 3, 387-392 (2008).
527	Not relevant	Hogarth, L.W., Burkett, B.J. & McKean, M.R. Activity profiles and physiological responses of tag football referees: A case study.
528	Not relevant	Holopainen, S., Szeróvay, M., Konttinen, N. & Kokkonen, M. Leadership in Finnish elite football: associations between players' self-assessed leadership roles and player-assessed head coach leadership behavior by player gender and age. Science & Medicine in Football 8, 301-307 (2023).
529	Not relevant	Holt, N.L., Tamminen, K.A., Black, D.E., Mandigo, J.L. & Fox, K.R. Youth Sport Parenting Styles and Practices. Journal of Sport & Exercise Psychology 31, 37-59 (2009).
530	Not relevant	Hong, E., Jeong, Y. & Downward, P. Perceived organizational support, internal motivation, and work–family conflict among soccer referees. Managing Sport & Leisure 24, 141-154 (2019).

531	Not relevant	Hongyou, L., Gómez, M.A., Lago-Peñas, C., Arias-Estero, J. & Stefani, R. Match Performance Profiles of Goalkeepers of Elite Football Teams. International Journal of Sports Science & Coaching 10, 669-682 (2015).
532	Not relevant	Honnor, S. Five decades of service to bowls. Bowls International, 38-40 (2023).
533	Not relevant	Hopkins, W.G. The Ninth World Congress on Science and Football. Sportscience, 1-15 (2019).
534	Not relevant	Hopkinson, M., et al. Rugby league ball carrier injuries: The relative importance of tackle characteristics during the European Super League. "Eur J Sport Sci,[2020#5Year]IF->2.781#3.228" 22, 269-278 (2022).
535	Not relevant	Horan, D., et al. Injuries in elite-level women's football—a two-year prospective study in the Irish Women's National League. Scandinavian Journal of Medicine & Science in Sports 32, 177-190 (2022).
536	Not relevant	Hordvik, M., MacPhail, A. & Ronglan, L.T. Negotiating the complexity of teaching: a rhizomatic consideration of pre-service teachers' school placement experiences. Physical Education & Sport Pedagogy 24, 447-462 (2019).
537	Not relevant	Horn, C.M., Gilbert, J.N., Gilbert, W. & Lewis, D.K. Psychological Skills Training With Community College Athletes: The UNIFORM Approach. "Sport Psychol,[2020#5Year]IF->1.515#2.080" 25, 321-340 (2011).
538	Not relevant	Horn, M., de Waal, S. & Kraak, W. In-match penalty kick analysis of the 2009/10 to 2018/19 English Premier League competition. "International Journal Of Performance Analysis In Sport,[2020#5Year]IF->1.518#1.707" 21, 139-155 (2021).
539	Not relevant	Hossner, E.-J., Schnyder, U., Schmid, J. & Kredel, R. The role of viewing distance and viewing angle on referees' decision-making performance during the FIFA World Cup 2014. "J Sports Sci,[2020#5Year]IF->2.597#3.060" 37, 1481-1489 (2019).
540	Not relevant	Hossner, EJ; Schnyder, U; Schmid, J; Kredel, R.The role of viewing distance and viewing angle on referees' decision-making performance during the FIFA World Cup 2014

S1 File. Studies Identification

541	Not relevant	Houssein, M., et al. Hydration: The New FIFA World Cup's Challenge for Referee Decision Making? Journal of Athletic Training (Allen Press) 51, 264-266 (2016).
542	Not relevant	Houssein, M; Lopes, P; Fagnoni, B; Ahmaidi, S; Yonis, SM; Leprêtre, PM
543	Not relevant	Hristov, M. Obremenitve nogometnih sodnikov med tekmo. Revija Šport 67, 130-134 (2019).
544	Not relevant	Hrusa, P; Orel, P.COMPARISON OF PHYSICAL PREPAREDNESS OF FOOTBALL REFEREES OF DIFFERENT COMPETITION LEVELS
545	Not relevant	Huang, J. & Hsu, H.-J. Approximating strike zone size and shape for baseball umpires under different conditions. "International Journal Of Performance Analysis In Sport,[2020#5Year]IF->1.518#1.707" 20, 133-149 (2020).
546	Not relevant	Huberty, J.L., Beets, M.W., Beighle, A. & McKenzie, T.L. Association of Staff Behaviors and Afterschool Program Features to Physical Activity: Findings From Movin' After School. Journal of Physical Activity & Health 10, 423-429 (2013).
547	Not relevant	Hughes, M. & Smyth, G. Profiling half-back play in rugby union and the impact of substitutions. Journal of Human Sport & Exercise 13, 940-962 (2018).
548	Not relevant	Hunt, T. Ireland's footballers at the 1924 and 1948 Olympic Games: compromised by the politics of sport. Soccer & Society 22, 887-900 (2021).
549	Not relevant	Hunzinger, K.J., et al. Effects of contact/collision sport history on gait in early- to mid-adulthood. J Sport Health Sci 12, 398-405 (2023).
550	Not relevant	Hurka, T. On Judged Sports. "J Phil Sport,[2020#5Year]IF->0.867#0.814" 42, 317-325 (2015).
551	Not relevant	Hussain, F.M., Shuhaib, M.H. & Ali Hassan, M.F. Psychological Toughness and its Relationship to Some Coordination, Physical Abilities and Accuracy of Some Basic Skills Performance Among The Iraqi Junior National Handball Team Players. International Journal of Disabilities Sports & Health Sciences 7, 330-336 (2023).

S1 File. Studies Identification

552	Not relevant	Hutzler, Y. & Bar-Eli, M. How to Cope with Bias While Adapting for Inclusion in Physical Education and Sports: A Judgment and Decision-Making Perspective. Quest (00336297) 65, 57-71 (2013)
553	Not relevant	Hyatt, C.G. & Foster, W.M. Using Identity Work Theory to Understand the De-Escalation of Fandom: A Study of Former Fans of National Hockey League Teams. "J Sport Manage,[2020#5Year]IF->2.359#2.877" 29, 443-460 (2015).
554	Not relevant	Iannaccone, A., Fusco, A., Conte, D. & Cortis, C. NOTATIONAL ANALYSIS OF BEACH HANDBALL. Human Movement 23, 69-79 (2022).
555	Not relevant	Ibañez, S.J., Garcia-Rubio, J., Gómez, M.-Á. & Gonzalez-Espinosa, S. The Impact of Rule Modifications on Elite Basketball Teams' Performance. "Journal Of Human Kinetics,[2020#5Year]IF->1.664#1.886" 64, 181-193 (2018).
556	Not relevant	Ibáñez, S.J., Pérez-Goye, J.A., Courel-Ibáñez, J. & García-Rubio, J. The impact of scoring first on match outcome in women's professional football. "International Journal Of Performance Analysis In Sport,[2020#5Year]IF->1.518#1.707" 18, 318-326 (2018).
557	Not relevant	Ibrahim, H.G. & Hussein, M.G. Effect of speed, agility and quickness (SAQ) exercises on the development of physical abilities of soccer referees in Iraq.
558	Not relevant	Ignatov, G. & Naydenova, K. Characteristics and state of the physical loads in elite football referees in Bulgaria
559	Not relevant	III International Conference on Technologies applied to Physical Activity and Sport. TAPAS 2022. 16th-17th November 2022. Revista Andaluza de Medicina del Deporte 16, S1-S12 (2023).
560	Not relevant	Ikumi, A., Sasaki, E., Sakuyama, N. & Mikami, Y. Incidence of Elbow Injury Patterns in Japanese
561	Not relevant	Ilham, et al. The Effect of Combination of Cone Drill (Zigzag) with Core Stability, Combination of Ladder Drill (Snake Jump) with Core Stability, and Speed on Agility of Futsal Players: A Factorial Experimental Design. Retos: Nuevas Perspectivas de Educación Física, Deporte y Recreación 58, 1-11 (2024).

S1 File. Studies Identification

562	Not physical or physiological variables	Ilhan A, Muniroglu S, Rakıcıoğlu N.Effect of body composition on the athletic performance of soccer referees
563	Not relevant	Ilhan, A., Muniroglu, S. & Rakıcıoğlu, N. Effect of body composition on the athletic performance of soccer referees. J Nutr Sci 12, e66 (2023).
564	Not relevant	İnal, R. The Big Story of a "Small" Football Club: Gümüşlükspor as an Alternative Model Experience for Turkey. "Sociol Sport J,[2020#5Year]IF->2.635#2.272" 40, 330-337 (2023).
565	Not relevant	Inan, T. & Cavas, L. DID FOOTBALL PLAYERS AVOID PHYSICAL INTERACTION IN THE GAMES AFTER COVID-19? Revista Brasileira de Futsal e Futebol 14, 189-200 (2022).
566	Not relevant	Index to Abstract Descriptors. "Psychophysiology,[2020#5Year]IF->3.692#3.793" 59, S193-S197 (2022).
567	Not relevant	Ingham, A.G. & Loy Jr, J.W. The Social System of Sport: a Humanistic Perspective. Quest (00336297) 19, 3-23 (1973).
568	Not relevant	Inglés-Bolumar, P., Pino-Ortega, J., Bastida-Castillo, A. & Gómez-Carmona, C.D. Análisis Cinemático de las Exigencias en Futbolistas de Categoría Benjamín Mediante un Dispositivo Inercial (WIMU PROTM). Revista Kronos 17, 1-11 (2018).
569	Not relevant	Inoue, Y., Sato, M. & Filo, K. Transformative Sport Service Research: Linking Sport Services With Well-Being. "J Sport Manage,[2020#5Year]IF->2.359#2.877" 34, 285-290 (2020).
570	Not relevant	Insook, K., Yun Soo, L., Ward, P. & Weidong, L. A Critical Examination of Movement Content Knowledge Courses in Physical Education Teacher Education Programs. "J Teach Phys Educ,[2020#5Year]IF->1.845#2.490" 34, 59-75 (2015).
571	Not relevant	International Council for Coaching Excellence (ICCE) 13th Global Coach Conference. International Sport Coaching Journal 8, S1-S80 (2021).

S1 File. Studies Identification

572	Not relevant	International Journal of Sport and Exercise Psychology (IJSEP). International Journal of Sport & Exercise Psychology 19, S1-S539 (2021).
573	Not relevant	Invernizzi, P.L., et al. INTERPRETATION AND PERCEPTION OF TWO DIFFERENT KUMITE FIGHTING INTENSITIES THROUGH AN INTEGRATED APPROACH TRAINING IN INTERNATIONAL LEVEL KARATEKAS: AN EXPLORATORY STUDY. Perceptual & Motor Skills 121, 333-349 (2015).
574	Not relevant	Işın, A. & Gómez Ruano, M. How the 12th Man Influences Football Matches: The Role of Fans and Referees in the Home Advantage Phenomenon. "Percept Mot Skills,[2020#5Year]IF->1.245#1.276" 130, 2177-2188 (2023).
575	Not relevant	Işın, A. & Gómez Ruano, M.Á. How the 12th Man Influences Football Matches: The Role of Fans and Referees in the Home Advantage Phenomenon. Perceptual & Motor Skills 130, 2177-2188 (2023).
576	Not relevant	IŞIn, A. Video Yardımcı Hakem Uygulamasının Hakem Kararları Üzerine Etkileri: Tanımlayıcı Araştırma. Turkiye Klinikleri Journal of Sports Sciences 15, 237-242 (2023).
577	Not relevant	Islam, M.S. & De, A. Ancient Boxing: A Narrative Discussion from Archaeological and Historical Evidences. Montenegrin Journal of Sports Science & Medicine 11, 71-78 (2022).
578	Conference Paper	Itoh, H., Takiguchi, T. & Ariki, Y. Event detection and recognition using HMM with whistle sounds.
579	Not relevant	Itoh, H; Takiguchi, T; Ariki, Y.Event Detection and Recognition Using HMM with Whistle Sounds
580	Not relevant	Iwuagwu, T.E., Umeifekwem, J.E., IgweA, S.N., Oforka, O.K. & Udeh, O.P. Profile of sport competition anxiety trait and psychological coping skills among secondary school athletes in Enugu State, South East Nigeria. Baltic Journal of Health & Physical Activity 13, 75-87 (2021).
581	Not relevant	Izzicupo, P., Petri, C., Serafini, S., Galanti, G. & Mascherini, G. Morphological Characteristics of Elite International Soccer Referees: Somatotype and Bioelectrical Impedance Vector Analysis. Journal of Functional Morphology & Kinesiology 8, 100 (2023).

S1 File. Studies Identification

582	Not relevant	Jacinto, A.L.B., Praça, G.M., Pinheiro, G.d.S. & da Costa, V.T. Identification of indicators that predict victory in the five main CONMEBOL and UEFA leagues. International Journal of Sports Science & Coaching 19, 2078-2089 (2024).
583	Not relevant	Jacobs, K., Riveros, D., Vincent, H.K. & Herman, D.C. The effect of landing surface on landing error scoring system grades. "Sport Biomech,[2020#5Year]IF->2.023#1.949" 20, 190-197 (2021).
584	Not relevant	James, G. The origins of school football associations: Manchester as a case study. "Sport in Society,[2020#5Year]IF->0.939#Not Available" 23, 1370-1387 (2020).
585	Not relevant	James, K. & Nadan, Y. Race, ethnicity, and class issues in Fiji soccer 1980-2015. Soccer & Society 21, 741-761 (2020).
586	Not relevant	Jamil, M. A case study assessing possession regain patterns in English Premier League Football. "International Journal Of Performance Analysis In Sport,[2020#5Year]IF->1.518#1.707" 19, 1011-1025 (2019).
587	Not relevant	Jamil, M. Where do the best technical football players in the world come from? Analysing the association between technical proficiency and geographical origin in elite football. Journal of Human Sport & Exercise 17, 244-260 (2022).
588	Not relevant	Jazvin, A., PaliĆ, A.D.I., AdemoviĆ, A. & Skender, N. CORRELATION BETWEEN SPRINT, AGILITY AND VERTICAL JUMP OF ELITE SOCCER PLAYERS. Ovidius University Annals, Series Physical Education & Sport/Science, Movement & Health 21, 229-233 (2021).
589	Not relevant	Jeffress, M.S. & Brown, W.J. Opportunities and Benefits for Powerchair Users Through Power Soccer. "Adapt Phys Act Quart,[2020#5Year]IF->1.462#2.336" 34, 235-255 (2017).
590	Not relevant	Jelmer De Jong, J.-P., et al. THE INFLUENCE OF THE FUTSAL OUTFIELD GOALKEEPER ON PLAYERS' RUNNING PERFORMANCE. Human Movement 23, 49-55 (2022).
591	Not relevant	Jenkins, J.M. & Alderman, B.L. Influence of Sport Education on Group Cohesion in University Physical Education. "J Teach Phys Educ,[2020#5Year]IF->1.845#2.490" 30, 214-230 (2011).

592	Not relevant	Jensen, P., Roman, J., Shaft, B. & Wrisberg, C. In the Cage: MMA Fighters' Experience of Competition. "Sport Psychol,[2020#5Year]IF->1.515#2.080" 27, 1-12 (2013).
593	Not relevant	Jermyn, S., Neill, C.O., Lacey, S. & Coughlan, E.K. THE ACUTE EFFECTS OF A WEIGHTED FOOTBALL TRAINING INTERVENTION IN GAELIC FOOTBALL TO MAXIMISE PLACE-KICK BALL VELOCITY. Journal of Australian Strength & Conditioning 30, 18-28 (2022).
594	Not relevant	Jermyn, S., O'Neill, C., Lacey, S. & Coughlan, E.K. The Effects of a Weighted Football Intervention on Ball Velocity of a Standard Football Place-Kick among Elite Gaelic Football Goalkeepers: A Single-Subject Designed Study. Sports (2075-4663) 10, 166 (2022).
595	Not relevant	Jerome, B.W.C., et al. The influence of ball in/out of play and possession in elite soccer: Towards a more valid measure of physical intensity during competitive match-play. "Eur J Sport Sci,[2020#5Year]IF->2.781#3.228" 23, 1892-1902 (2023).
596	Not relevant	Jessiman, S.W., Harvey, B., Corrigan, S.L. & Gastin, P.B. Training and Competition Activity Profiles of Australian Football Field Umpires.
597	Not relevant	Jewiss, M., Runswick, O.R. & Greenlees, I. An Examination of the Challenge/Threat State and Sport-Performance Relationship While Controlling for Past Performance. Journal of Sport & Exercise Psychology 45, 195-207 (2023).
598	Not physical or physiological variables	Jiang, JY; Ge, HM; Du, LD; Gomez, MA; Gong, BN; Cui, YX.Impact of Match Type and Match Halves on Referees' Physical Performance and Decision-Making Distance in Chinese Football Super League
599	Not relevant	Jiménez-Salas, J., Morillo-Baro, J.P., Reigal, R.E., Morales-Sánchez, V. & Hernández-Mendo, A. Polar Coordinate Analysis to Study Counterattacks in Senior and Under-16 Men's Handball. Cuadernos de Psicología del Deporte 20, 48-61 (2020).
600	Not relevant	Jinying, J., Huanmin, G. & Yixiong, C. Effect of tournament format change on team performance of Chinese Football Super League during COVID-19 pandemic. RICYDE. Revista Internacional de Ciencias del Deporte 18, 72-85 (2022).

S1 File. Studies Identification

601	Not relevant	Johansson, M., Ervasti, P.-E. & Blomqvist, S. An Analysis of Acceleration, Deceleration and High-Intensity Skating during Elite Bandy Match-Play: A Case Study. Sports (2075-4663) 9, 152 (2021).
602	Not relevant	Johnson, C. & Taylor, J. Spilled milk and burned toast: extrinsic pressure and sporting excellence. "J Phil Sport,[2020#5Year]IF->0.867#0.814" 48, 202-218 (2021).
603	Not relevant	Johnston, L. & McNaughton, L. The physiological requirements of soccer refereeing.
604	Not relevant	Johnston, R. On referee bias, crowd size, and home advantage in the English soccer Premiership.
605	Not relevant	Jones, C. Judging athletes' moral actions: some critical reflections. "J Phil Sport,[2020#5Year]IF->0.867#0.814" 46, 1-13 (2019).
606	Not relevant	Jones, K.W. Female Fandom: Identity, Sexism, and Men's Professional Football in England. "Sociol Sport J,[2020#5Year]IF->2.635#2.272" 25, 516-537 (2008).
607	Not relevant	Jones, M.V. Controlling Emotions in Sport. "Sport Psychol,[2020#5Year]IF->1.515#2.080" 17, 471-486 (2003).
608	Not relevant	Joo, C.H. & Jee, H. Activity Profiles of Top-Class Players and Referees and Accuracy in Foul Decision-Making During Korean National League Soccer Games. "J Strength Cond Res,[2020#5Year]IF->2.973#3.058" 33, 2530-2540 (2019).
609	Not relevant	Joo, CH; Jee, H.ACTIVITY PROFILES OF TOP-CLASS PLAYERS AND REFEREES AND ACCURACY IN FOUL DECISION-MAKING DURING KOREAN NATIONAL LEAGUE SOCCER GAMES
610	Not relevant	Jordan, P., Upright, P. & Forsythe, S. Rural Kentucky Sport Officials' Perspectives on Recruitment, Training and Retention. Kentucky Newsletter for Health, Physical Education, Recreation & Dance 56, 59-72 (2019).
611	Not relevant	Jordet, G. & Hartman, E. Avoidance Motivation and Choking Under Pressure in Soccer Penalty Shootouts. Journal of Sport & Exercise Psychology 30, 450-457 (2008).

S1 File. Studies Identification

612	Not relevant	Julio, U.F. & Franchini, E. CHAPTER 1: Developing aerobic power and capacity for combat sports athletes. Revista de Artes Marciales Asiaticas 16, 10-59 (2021).
613	Not relevant	Junghagen, S. & Aurvandil, M. Structural Susceptibility to Corruption in FIFA: a Social Network Analysis. International Journal of Sport Policy & Politics 12, 655-677 (2020).
614	Not relevant	Kahraman, C., et al. Does the TMPRSS6 C > T Polymorphism Modify the Endurance Training Effects on Hematological Parameters?
615	Not relevant	Kalin, J. Finals Falls 1959-1986. Amateur Wrestling News 69, 14-21 (2023).
616	Not relevant	Kalkavan, A. & GÜNer, O. Investigation of Aggression Levels of Trabzonspor Fans. Sportif Bakış: Spor ve Eğitim Bilimleri Dergisi 10, 188-202 (2023).
617	Conference Paper	Kamble, P.R., Keskar, A.G. & Bhurchandi, K.M. A convolutional neural network based 3D ball tracking by detection in soccer videos.
618	Not relevant	Kanaan, E.M., Ahmed, M.D. & López-Sánchez, G.F. PARTICIPACIÓN DEPORTIVA, HÁBITOS DE VIDA Y DETERMINANTES DE LAS ESTUDIANTES DE JORDANIA: UN ANÁLISIS CUALITATIVO. Journal of Sport & Health Research 15, 345-356 (2023).
619	Not relevant	Kapsalis, M., et al. Exploring the impact of possession-based performance indicators on goal scoring in elite football leagues. Journal of Physical Education & Sport 23, 2004-2015 (2023).
620	Not relevant	Karabalcik, H., Saygin, Ö. & Ceylan, H.İ. FUTBOL HAKEMLERİNİN FİZİKSEL KAPASİTELERİ, MAÇ SIRASINDAKİ AKTİVİTE PROFİLLERİ VE KARAR VERME BECERİLERİNİN DENEYİMLERİNE GÖRE KARŞILAŞTIRILMASI. Congress Papers of The Association of Sports Sciences, 324-329 (2019).
621	Not relevant	Karaca, N., Turgay, F., Nalçakan, G.R., Nalçakan, M. & Şişman, A.R. Effects of a Volleyball Match on Serum Nitric Oxide Level and Oxidant/Antioxidant Status. Spor Hekimligi Dergisi/Turkish Journal of Sports Medicine 53, 27-36 (2018).

S1 File. Studies Identification

622	Not relevant	KaraÇAm, A., Sabuncu, A.A., AkÇA, E., Akduman, O.B. & GÜÇLÜ, H. Hakemlik Mesleğini Sürdürme Ölçeği'nin (HMSÖ) Türkçeye Uyarlama Çalışması. CBÜ Beden Eğitimi & Spor Bilimleri Dergisi 16, 9-21 (2021).
623	Not relevant	Karadag, T., Parim, C. & Cene, E. Selecting the best team from the players in the 2018 FIFA World Cup using performance analysis. International Sports Studies 42, 15-32 (2020).
624	Not relevant	Karafil, A.Y. & Akgül, M.H. INVESTIGATION ON SELF-EFFICACY ON JOB SATISFACTION OF SOCCER REFEREES. Kinesiologia Slovenica 27, 70-82 (2021).
625	Not relevant	KarafİL, A.Y. Examination of Football Referees' Attitudes Towards Video Assistant Referee System (VAR) by Q Method. Journal of Sports Sciences Research / Spor Bilimleri Araştırmaları Dergisi 8, 99-112 (2023).
626	Not relevant	Karagün, E., Selvi, S. & Kahveci, M.S. Analysis of healthy life styles and nutritional attitudes of football referees during the pandemic.
627	Not relevant	Kartal, A., et al. Analyses of the energy sources used by Turkish referees during ninety minutes soccer match.
628	Not relevant	Kasum, G. WRESTLING - SERBIAN OLYMPIC VALUE. Physical Culture / Fizicka Kultura 75, 44-55 (2021).
629	Not relevant	Kavussanu, M. & Spray, C.M. Contextual Influences on Moral Functioning of Male Youth Footballers. "Sport Psychol,[2020#5Year]IF->1.515#2.080" 20, 1-23 (2006).
630	Not relevant	Kay, B. & Gill, N.D. Physical demands of elite Rugby League referees, part two: heart rate responses and implications for training and fitness testing. "J Sci Med Sport,[2020#5Year]IF->3.607#4.332" 7, 165-173 (2004).
631	Not relevant	Kegelaers, J., Wylleman, P., Bunigh, A. & Oudejans, R.R.D. A Mixed Methods Evaluation of a Pressure Training Intervention to Develop Resilience in Female Basketball Players. "J Appl Sport Psychol,[2020#5Year]IF->2.150#2.420" 33, 151-172 (2021).
632	Not relevant	Kelly, G., et al. Benchmarking successful performances in elite Ladies Gaelic football. "International Journal Of Performance Analysis In Sport,[2020#5Year]IF->1.518#1.707" 22, 51-65 (2022).

S1 File. Studies Identification

633	Not relevant	Kerr, J.H. The enjoyment of sanctioned aggression in rugby: The experience of a pioneering female Canadian team captain. International Journal of Sport & Exercise Psychology 17, 578-590 (2019).
634	Not relevant	Kian, E.M., Vincent, J. & Mondello, M. Masculine Hegemonic Hoops: An Analysis of Media Coverage of March Madness. "Sociol Sport J,[2020#5Year]IF->2.635#2.272" 25, 223-242 (2008).
635	Not relevant	Kim, J., Ko, Y.J. & Connaughton, D.P. Performance Expectancy of Officiating Technology in Spectator-Based Sport Events: Scale Development and Validation. Communication & Sport 11, 528-550 (2023).
636	Not relevant	King, C. & Coughlan, E. Blowing the Whistle on Concussion Knowledge and Education in Youth Sport Referees. Open Access Journal of Sports Medicine 12, 109-117 (2021).
637	Not relevant	King, D.B. & Raymond, B.L. History of Sport Psychology in Cultural Magazines of the Victorian Era. "Sport Psychol,[2020#5Year]IF->1.515#2.080" 9, 376-390 (1995).
638	Not relevant	Kirk, C., Clark, D.R., Langan-Evans, C. & Morton, J.P. The physical demands of mixed martial arts: A narrative review using the ARMSS model to provide a hierarchy of evidence. "J Sports Sci,[2020#5Year]IF->2.597#3.060" 38, 2819-2841 (2020).
639	Not relevant	Kirk, D. Sport Education, Critical Pedagogy, and Learning Theory: Toward an Intrinsic Justification for Physical Education and Youth Sport. Quest (00336297) 58, 255-264 (2006).
640	Not relevant	KISA SÖZEL BİLDİRİ ÖZETLERİ. Congress Papers of The Association of Sports Sciences, 570-1205 (2017).
641	Not relevant	Kiss, B., Balogh, L., MÜNnich, Á. & Csillacsukonyi. A sport-psychological diagnostic examination of young EHF handball referees with a focus on mental skills. Journal of Physical Education & Sport 20, 1984-1995 (2020).
642	Not relevant	Kittel, A., Elsworthy, N. & Spittle, M. Incorporating perceptual decision-making training into high-intensity interval training for Australian football umpires.
643	Not relevant	Kittel, A., Elsworthy, N. & Spittle, M. The Effectiveness of Above Real Time Training for Developing Decision-Making Accuracy in Australian Football Umpires. Research Quarterly for Exercise & Sport 94, 64-72 (2023).

644	Not relevant	Kittel, A., Larkin, P., Elsworthy, N. & Spittle, M. Identification of key performance characteristics of elite Australian football umpires. International Journal of Sports Science & Coaching 14, 490-497 (2019).
645	Not relevant	Kittel, A., Larkin, P., Elsworthy, N. & Spittle, M. Transfer of 360° virtual reality and match broadcast video-based tests to on-field decision-making. Science & Medicine in Football 5, 79-86 (2021).
646	Not relevant	Kittel, A., Larkin, P., Elsworthy, N., Lindsay, R. & Spittle, M. Effectiveness of 360° virtual reality and match broadcast video to improve decision-making skill. Science & Medicine in Football 4, 255-262 (2020).
647	Not relevant	Kittel, A., Spittle, M. & Elsworthy, N. Incorporating perceptual decision-making training into high-intensity interval training for Australian football umpires. "J Sports Sci,[2020#5Year]IF->2.597#3.060" 37, 29-35 (2019).
648	Not relevant	Kizilet, A. Using distance physical education in elite class soccer referee training: A case study.
649	Not relevant	Klatt, S; Noël, B; Nicklas, A; Schul, K; Seifriz, F; Schwarting, A; Fasold, F
650	Not relevant	Kocakarin, E. Life Gets Even More Beautiful, During Extended 90 + Minutes. Turkish Journal of Sport & Exercise / Türk Spor ve Egzersiz Dergisi 25, 482-491 (2023).
651	Conference Paper	Koeipakvaen, T. Physical Fitness for Futsal Referee of Football Association in Thailand.
652	Conference Paper	Koeipakvaen, Thaweesub.Physical Fitness For Futsal Referee Of Football Association In Thailand
653	Not relevant	Kolbinger, O. The phenomenon of trivial offenses and why we should not just leave it to the referees. "J Phil Sport,[2020#5Year]IF->0.867#0.814" 48, 82-96 (2021).
654	Not relevant	Kons, R., Krabben, K., Mann, D.L. & Detanico, D. Effect of vision impairment on match-related performance and technical variation in attacking moves in Paralympic judo. "J Sports Sci,[2020#5Year]IF->2.597#3.060" 39, 125-131 (2021).

655	Not relevant	Kooistra, P. & Kooistra, R. The ins and outs of US youth soccer: learning about loyalty and success. Soccer & Society 19, 944-965 (2018).
656	Not relevant	Kordi, R., Chitsaz, A., Rostami, M., Mostafavi, R. & Ghadimi, M. Incidence, Nature, and Pattern of Injuries to Referees in a Premier Football (Soccer) League: A Prospective Study. Sports Health: A Multidisciplinary Approach 5, 438-441 (2013).
657	Not relevant	Korobeynikov, G., et al. Psychophysiological state and decision making in wrestlers. Ido Movement for Culture. Journal of Martial Arts Anthropology 22, 1-9 (2022).
658	Not relevant	Kossakowski, R. From Communist Fan Clubs to Professional Hooligans: A History of Polish Fandom as a Social Process. "Sociol Sport J,[2020#5Year]IF->2.635#2.272" 34, 281-292 (2017).
659	Not relevant	Kostiukevych, V., et al. Highly qualified grass hockey sportswomen's adaptation to training intensity in the macrocycle preparatory period. Journal of Physical Education & Sport 20, 385-394 (2020).
660	Not relevant	Kostrna, J. & Tenenbaum, G. Developing and testing the Expanded Sport Official's Decision-Making Model. International Journal of Sport & Exercise Psychology 20, 586-611 (2022).
661	Not relevant	Koutrou, N. & Kohe, G.Z. Conceptualising landscapes of learning in the United Kingdom's volunteer football sector. Sport, Education & Society 26, 982-997 (2021).
662	Not relevant	Kozina, Z., et al. Psycho-physiological characteristics of female basketball players with hearing problems as the basis for the technical tactic training methodic in world level. Journal of Physical Education & Sport 16, 1348-1359 (2016).
663	Not relevant	Kraak, W.J., Malan, D.D.J. & Van Den Berg, P.H. Time-motion analysis and heart rate recordings of South African rugby union referees. African Journal for Physical, Health Education, Recreation & Dance, 841-851 (2011).
664	Not relevant	Krawczyk, P., Dyjas, A., SzelĄG, A. & Cedro, M. The moment of the initiation of the save as a factor differentiating the actions of handball goalkeepers during throws. Journal of Physical Education & Sport 22, 518-527 (2022).

S1 File. Studies Identification

665	Not relevant	Krenn, B.Does uniform color affect offside in association football?
666	Selected	Krustrup and Bangsbo.2001physiological demands of top-class soccer refereeing in relation to physical capacity:effect of intense intermittent exercise training
667	Not relevant	Krustrup, P. & Bangsbo, J. Physiological demands of top-class soccer refereeing in relation to physical capacity: effect of intense intermittent exercise training. "J Sports Sci,[2020#5Year]IF->2.597#3.060" 19, 881-891 (2001).
668	Not relevant	Krustrup, P., et al. Activity profile and physical demands of football referees and assistant referees in international games. "J Sports Sci,[2020#5Year]IF->2.597#3.060" 27, 1167-1176 (2009).
669	Not relevant	Krustrup, P., Mohr, M. & Bangsbo, J. Activity profile and physiological demands of top-class soccer assistant refereeing in relation to training status. "J Sports Sci,[2020#5Year]IF->2.597#3.060" 20, 861 (2002).
670	Not relevant	Krustrup, P., Randers, M., Horton, J., Brito, J. & Rebelo, A. Ecological validity of the Yo-Yo SFIE2 test.
671	Not relevant	Krustrup, P; Helsen, W; Randers, MB; Christensen, JF; MacDonald, C; Rebelo, AN; Bangsbo, J
672	Not relevant	Kubayi, A. & Toriola, A. Match Performance Indicators that Discriminated Between Winning, Drawing and Losing Teams in the 2017 AFCON Soccer Championship. "Journal Of Human Kinetics,[2020#5Year]IF->1.664#1.886" 72, 215-221 (2020).
673	Not relevant	Kubayi, A. & Toriola, A. The Influence of Situational Variables on Ball Possession in the South African Premier Soccer League. "Journal Of Human Kinetics,[2020#5Year]IF->1.664#1.886" 66, 175-181 (2019).
674	Not relevant	Kubayi, A. Technical demands of the various playing positions in the qualifying matches for the European football championship. "International Journal Of Performance Analysis In Sport,[2020#5Year]IF->1.518#1.707" 21, 374-382 (2021).

S1 File. Studies Identification

675	Not relevant	KÜÇÜK, H. Futbol Hakemlerinin Çeviklik, Sürat ve Aerobik Kapasitelerinin Karşılaştırılması. Mediterranean Journal of Sport Science (MJSS) 5, 713-721 (2022).
676	Not relevant	Kucuk, H., Soyler, M., Ceylan, T., Ceylan, L. & Sahin, F.N. Effects of acute and chronic high-intensity interval training on serum irisin, BDNF and apelin levels in male soccer referees.
677	Not relevant	Kural, S. & Aydin, F. Examining self-efficacy levels of football referees. Baltic Journal of Health & Physical Activity 13, 123-130 (2021).
678	Not relevant	Kural, S. & Aydin, F. Futbol Hakemlerinin Serbest Zaman Doyum Düzeyleri ile Psikolojik Sağlamlık İlişkisinin İncelenmesi. Sportif Bakış: Spor ve Eğitim Bilimleri Dergisi 10, 14-25 (2023).
679	Not relevant	Kural, S; Aydin, F.Examining self-efficacy levels of football referees
680	Conference Paper	Kürkçü, C. & Uluşar, U.D. Classification of movement patterns of soccer referees using K-means.
681	Not relevant	Kürkçü, C; Ulusar, ÜD.Position and Motion Analysis of Referees During Soccer Games
682	Conference Paper	Kurkcu, Cengiz; Ulusar, Umit Deniz.Position and Motion Analysis of Referees During Soccer Games
683	Conference Paper	Kusumah, W., Rizka, M. & Nurcahya, Y. The implementation of training methods and the ability of basic speed 100M on the physical improvement of football referees.
684	Not relevant	Lacasa, E., Canton, A., Brufau, I., March-Llanes, J. & Torrents, C. Rink hockey "Ok-XS". Motor behavior effects of scaling games in U8 players. International Journal of Sports Science & Coaching 18, 812-821 (2023).
685	Not relevant	Lacerda, T. Education for the Aesthetics of Sport in Higher Education in the Sports Sciences – The Particular Case of the Portuguese-Speaking Countries. "J Phil Sport,[2020#5Year]IF->0.867#0.814" 39, 235-250 (2012).

686	Not relevant	Lago-Peñas, C. & Gómez-López, M. The Influence of Referee Bias on Extra Time in Elite Soccer Matches. "Percept Mot Skills,[2020#5Year]IF->1.245#1.276" 122, 666-677 (2016).
687	Not relevant	Lago-Peñas, C. & Gómez-López, M. The Influence of Referee Bias on Extra Time in Elite Soccer Matches. Perceptual & Motor Skills 122, 666-677 (2016).
688	Not relevant	Lago-Peñas, C. The Role of Situational Variables in Analysing Physical Performance in Soccer. "Journal Of Human Kinetics,[2020#5Year]IF->1.664#1.886" 35, 89-95 (2012).
689	Not relevant	Lago-Peñas, C., Gómez, M.A. & Pollard, R. The effect of the Video Assistant Referee on referee's decisions in the Spanish LaLiga. International Journal of Sports Science & Coaching 16, 824-829 (2021).
690	Not relevant	Lago-Peñas, C; Gómez, MA; Pollard, R.The effect of the Video Assistant Referee on referee's decisions in the Spanish LaLiga
691	Not relevant	Lake, R.J. "Oft Incandescent on the Green Sward": The Incipient Development of Lawn Tennis in Ontario, 1870–1900. Journal of Sport History 50, 315-343 (2023).
692	Not relevant	Larkin, P., et al. Assessment of decision-making performance and in-game physical exertion of Australian football umpires. "J Sports Sci,[2020#5Year]IF->2.597#3.060" 32, 1446-1453 (2014).
693	Not relevant	Larkin, P., Mesagno, C., Berry, J. & Spittle, M. Exploration of the perceptual-cognitive processes that contribute to in-game decision-making of Australian football umpires. International Journal of Sport & Exercise Psychology 16, 112-124 (2018).
694	Not relevant	Larkin, P., Mesagno, C., Berry, J., Spittle, M. & Harvey, J. Video-based training to improve perceptual-cognitive decision-making performance of Australian football umpires. "J Sports Sci,[2020#5Year]IF->2.597#3.060" 36, 239-246 (2018).
695	Not relevant	Larkin, P; O'Brien, B; Mesagno, C; Berry, J; Harvey, J; Spittle, M.Assessment of decision-making performance and in-game physical exertion of Australian football umpires

S1 File. Studies Identification

696	Not relevant	Larner, S. LAWS OF ATTRACTION. Rugby World, 48-53 (2020).
697	Not relevant	Lastella, M., et al. The influence of training and competition on sleep behaviour of soccer referees. Science & Medicine in Football 6, 98-104 (2022).
698	Not relevant	Lategan, L. Physiological profiles of South African soccer referees and assistant referees. African Journal for Physical, Health Education, Recreation & Dance 17, 675-693 (2011).
699	Not relevant	Latinjak, A.T. Goal-Directed, Spontaneous, and Stimulus-Independent Thoughts and Mindwandering in a Competitive Context. "Sport Psychol,[2020#5Year]IF->1.515#2.080" 32, 51-59 (2018).
700	Not relevant	Leguizamo, F., Núñez, A., Gervilla, E., Llabrés, J. & Garcia-Mas, A. Effectiveness of a Program to Improve the Verbal Response of Football Coaches: A Delayed-Intervention Experimental Design. Retos: Nuevas Perspectivas de Educación Física, Deporte y Recreación 51, 657-665 (2024).
701	Not relevant	Leicht, A.S., Connor, J., Conduit, N., Vaquera, A. & Gómez, M.A. Impact of Match Type on Exercise Volume and Intensity of Semi-Professional Basketball Referees During a Competitive Season. Research Quarterly for Exercise & Sport 92, 843-850 (2021).
702	Not relevant	Leite, W. & Figueredo, R. IS THERE A NEED TO INCREASE THE NUMBER OF SUBSTITUTIONS IN MODERN PROFESSIONAL FOOTBALL? Physical Culture / Fizicka Kultura 74, 5-18 (2020).
703	Not relevant	Leite, W.S.S. & Almeida, C.H. Competitive-level and mid-term effects on the magnitude of home advantage in Portuguese futsal. "International Journal Of Performance Analysis In Sport,[2020#5Year]IF->1.518#1.707" 18, 184-194 (2018).
704	Not relevant	Leota, J. & Turp, M.-J. Gamesmanship as strategic excellence. "J Phil Sport,[2020#5Year]IF->0.867#0.814" 47, 232-247 (2020).
705	Not relevant	Leota, J., et al. Home is where the hustle is: the influence of crowds on effort and home advantage in the National Basketball Association. "J Sports Sci,[2020#5Year]IF->2.597#3.060" 40, 2343-2352 (2022).

S1 File. Studies Identification

706	Not relevant	Let's police offside line better. Rugby World, 16-16 (2023).
707	Not relevant	Lewis, M., McNicholas, E., McCarthy, A.-M. & Sherwin, I. Analysis of referee in-game interactions with players and other officials in professional Rugby Union. "International Journal Of Performance Analysis In Sport,[2020#5Year]IF->1.518#1.707" 23, 232-248 (2023).
708	Conference Paper	Li, S; Cheng, Y; Dai, Y; Ma, J。Kinect Based Virtual Referee For Table Tennis Game: TTV (Table Tennis Var System)
709	Not relevant	Ličen, S., Frandsen, K., Horky, T., Onwumechili, C. & Wei, W. Rediscovering Mediatization of Sport. Communication & Sport 10, 795-810 (2022).
710	Not relevant	Lima e Silva, L., et al. The haemodynamic demand and the attributes related to the displacement of the soccer referees in the moments of decision / intervention during the matches. "International Journal Of Performance Analysis In Sport,[2020#5Year]IF->1.518#1.707" 20, 219-230 (2020).
711	Not relevant	Lima, Y., Devran, S., Öz, N.D., Webb, T. & Bayraktar, B. Examining the mental health status of referees in the turkish professional football league. Science & Medicine in Football 7, 272-278 (2023).
712	Not relevant	Link, D. & Anzer, G. How the COVID-19 Pandemic has Changed the Game of Soccer. "Int J Sports Med,[2020#5Year]IF->2.556#2.616" 43, 83-93 (2022).
713	Not relevant	Link, D; Anzer, G.How the COVID-19 Pandemic has Changed the Game of Soccer
714	Not relevant	Linke, D., Link, D., Weber, H. & Lames, M. Decline in Match Running Performance in Football is affected by an Increase in Game Interruptions. Journal of Sports Science & Medicine 17, 662-667 (2018).
715	Not relevant	Lirgg, C.D., Feltz, D.L. & Merrie, M.D. Self-Efficacy of Sports Officials: A Critical Review of the Literature. Journal of Sport Behavior 39, 39-50 (2016).

716	Not relevant	Liu, H., Gómez, M.-A., Gonçalves, B. & Sampaio, J. Technical performance and match-to-match variation in elite football teams. "J Sports Sci,[2020#5Year]IF->2.597#3.060" 34, 509-518 (2016).
717	Not relevant	Liu, H., Wang, W., Zhang, C. & Hastie, P.A. College Students' Development of Badminton Skills and Tactical Competencies Following Play Practice. "J Teach Phys Educ,[2020#5Year]IF->1.845#2.490" 40, 284-292 (2021).
718	Not relevant	Liu, T., García-De-Alcaraz, A., Zhang, L. & Zhang, Y. Exploring home advantage and quality of opposition interactions in the Chinese Football Super League. "International Journal Of Performance Analysis In Sport,[2020#5Year]IF->1.518#1.707" 19, 289-301 (2019).
719	Conference Paper	Liu, Y. & Liu, D. Movement status based vision filter for RoboCup small-size league.
720	Not relevant	Livings, B. & Wlodarczak, K. Procedural Fairness in the International Tennis Federation's Disciplinary Regime. Entertainment & Sports Law Journal 18, 1-12 (2020).
721	Not relevant	Loadman, A. 'He's Running, Ref!' An ethnographic study of walking football. Soccer & Society 20, 675-692 (2019).
722	Not relevant	Löbig, A., Schlesinger, T., Breuer, M. & Ehnold, P. Analysis of factors influencing German youth football players' club membership with a particular focus on dropout. Soccer & Society 24, 190-207 (2023).
723	Not relevant	Lobo-Triviño, D., Ponce-Bordón, J.C., Llanos-Muñoz, R., López del Campo, R. & López-Gajardo, M.A. Does the final ranking influence the physical performance of professional soccer teams? Cultura, Ciencia y Deporte 18, 153-171 (2023).
724	Not relevant	Lobo-Triviño, D., Ponce-Bordón, J.C., Llanos-Muñoz, R., López del Campo, R. & López-Gajardo, M.A. Does the final ranking influence the physical performance of professional soccer teams? Cultura, Ciencia y Deporte 18, 153-171 (2023).
725	Not relevant	Loghmani, M., Cuskelly, G. & Webb, T. Examining the career dynamics of elite football referees: a unique identification profile. "Sport Management Review,[2020#5Year]IF->3.337#3.761" 24, 517-542 (2021).

726	Not relevant	Lohani, P; Wiley, T.Hybrid Methods for Real-Time Video Sequence Identification of Human Soccer Referee Signals
727	Not relevant	Loland, S. Sport: a scientific experiment? "Sport in Society,[2020#5Year]IF->0.939#Not Available" 22, 1501-1511 (2019).
728	Not relevant	Lombard, G. & Cloes, M. Analysis of the relevance of the information content given to the players during volleyball timeouts with a 3D device. "International Journal Of Performance Analysis In Sport,[2020#5Year]IF->1.518#1.707" 21, 965-980 (2021).
729	Not relevant	Long, T., Pantaléon, N., Bruant, G. & d'Arripe-Longueville, F. A Qualitative Study of Moral Reasoning of Young Elite Athletes. "Sport Psychol,[2020#5Year]IF->1.515#2.080" 20, 330-347 (2006).
730	Not relevant	Longas Luque, C. & van Sterkenburg, J. Exploring Discourses About Race/Ethnicity in a Spanish TV Football Program. Communication & Sport 10, 1113-1133 (2022).
731	Not relevant	Longo, U.G., et al. The influence of athletic performance on the highest positions of the final ranking during 2017/2018 Serie A season. BMC Sports Science, Medicine & Rehabilitation 13, 1-8 (2021).
732	Not relevant	López-Aguilar, J., Alonso-Arbiol, I., Onetti-Onetti, W. & Castillo-Rodríguez, A. Efecto de la competición sobre la impulsividad del árbitro de fútbol amateur. Cultura, Ciencia y Deporte 16, 519-528 (2021).
733	Not relevant	López-Gajardo, M.A., González-Ponce, I., Pulido, J.J., García-Calvo, T. & Leo, F.M. ANALYSIS OF THE FOOTBALL GOALKEEPER'S TECHNICAL-TACTICAL ACTIONS IN COMPETITION. International Journal of Medicine & Science of Physical Activity & Sport / Revista Internacional de Medicina y Ciencias de la Actividad Física y del Deporte 20, 577-594 (2020).
734	Not relevant	López-García, R., et al. Morphological characteristics in professional soccer referees in mexico; anthropometry and dxa.

S1 File. Studies Identification

735	Not relevant	López-García, R., Lagunes-Carrasco, J.O., Carranza-García, L.E., Ródenas-Cuenca, L.T. & Morales-Corral, P.G. CARACTERÍSTICAS MORFOLÓGICAS EN ÁRBITROS DE FÚTBOL PROFESIONAL EN MÉXICO; ANTROPOMETRÍA Y DEXA. International Journal of Medicine & Science of Physical Activity & Sport / Revista Internacional de Medicina y Ciencias de la Actividad Física y del Deporte 21, 117-129 (2021).
736	Not relevant	López-García, R; Lagunes-Carrasco, JO; Carranza-García, LE; Ródenas-Cuenca, LT; Morales-Corral, PG
737	Not relevant	López-Samanes, Á., Moreno-Pérez, V., Travassos, B. & Del Coso, J. Effects of acute caffeine ingestion on futsal performance in sub-elite players. "Eur J Nutr,[2020#5Year]IF->4.664#4.348" 60, 4531-4540 (2021).
738	Not relevant	Loudcher, J.-F. & Fabian, T. The first elite sport training camp in France: Manitot (1912–1924). "Eur J Sport Sci,[2020#5Year]IF->2.781#3.228" 20, 1387-1394 (2020).
739	Not relevant	Loureiro da Silva, V., et al. PHYSIOLOGICAL RESPONSES ASSOCIATED WITH CARDIOPULMONARY EXERCISE TESTING IN ELITE SOCCER REFEREES, DISTANCE RUNNERS AND HEALTH CONTROLS. Revista Brasileira de Prescrição e Fisiologia do Exercício 14, 48-55 (2020).
740	Not relevant	Lovelass, E. CAN SHE PLAY? THE JOURNEY OF A FEMALE ATHLETE IN THE INDUSTRY OF BASEBALL. Mississippi Sports Law Review 9, 54-67 (2020).
741	Not relevant	Lucas, J. "FRANCE VERSUS U.S.A. IN 1924 OLYMPIC GAMES RUGBY: EFFORTS TO ASSUAGE TRANSNATIONAL TENSION". Canadian Journal of History of Sport 19, 15-27 (1988).
742	Conference Paper	Lucic, I., Babic, S. & Vuckov, D. Perception of Using VAR Technology in Football after Completion of Training and Education and Experiences of Croatian Video Assistant Referees (VARs) and Assistant VARs (AVARs).
743	Not relevant	Luguetti, C., Goodyear, V.A. & André, M.H. 'That is like a 24 hours-day tournament!': using social media to further an authentic sport experience within sport education. Sport, Education & Society 24, 78-91 (2019).

S1 File. Studies Identification

744	Not relevant	Luis Del Campo, V. & Morenas Martín, J. Influence of Video Speeds on Visual Behavior and Decision-Making of Amateur Assistant Referees Judging Offside Events.
745	Not relevant	Luis del Campo, V., Morenas Martín, J. & Pizzera, A. Effects of past and current motor experiences as soccer players in decision-making of amateur soccer referees. International Journal of Sport & Exercise Psychology 20, 1102-1116 (2022).
746	Not relevant	Luna, W.D., Sur, M.H. & Shapiro, D.R. Using Paralympic School Day and Sport Education Model to Increase Awareness and Inclusion of Students With Disabilities in Physical Education. JOPERD: The Journal of Physical Education, Recreation & Dance 94, 5-15 (2023).
747	Not relevant	Lunander, A. & Karlsson, N. Choosing opponents in skiing sprint elimination tournaments. Journal of Quantitative Analysis in Sports 19, 205-221 (2023).
748	Not relevant	Lupinek, J.M. The Rookie Experience: The Initiation of Unwritten Rule Development in Men's Ice Hockey. Journal of Sport Behavior 42, 63-90 (2019).
749	Not relevant	Lupo, C., et al. Elite hit ball performance profile: technical, tactical and heart rate aspects, and effects of competition on jump and strength performance. RICYDE. Revista Internacional de Ciencias del Deporte 14, 111-123 (2018).
750	Not relevant	M.b. REFEREE. Sports Illustrated 134, 26-28 (2023).
751	Not high-level matches	MacDonald et al.,Physical demands and physiological strain of American football referees while officiating
752	Not relevant	MacDonald, H.V., et al. Physical demands and physiological strain of American football referees while officiating. "Phys Sportsmed,[2020#5Year]IF->1.662#1.971" 51, 351-360 (2023).
753	Not relevant	MacDonald, HV; Colster, EC; Mulholland, AM; Holmes, CJ; Bentley, BC; Robinson, JB; Wingo, JE

754	Not relevant	Mack, R.J., Breckon, J.D., O'Halloran, P.D. & Butt, J. Enhancing Athlete Engagement in Sport Psychology Interventions Using Motivational Interviewing: A Case Study. "Sport Psychol,[2020#5Year]IF->1.515#2.080" 33, 159-168 (2019).
755	Not relevant	Mackay, L., et al. Consensus on a netball video analysis framework of descriptors and definitions by the netball video analysis consensus group. "Br J Sports Med,[2020#5Year]IF->12.022#10.481" 57, 441-449 (2023).
756	Not relevant	MacMahon, C., Helsen, W., Starkes, J. & Weston, M. Decision-making skills and deliberate practice in elite association football referees. "J Sports Sci,[2020#5Year]IF->2.597#3.060" 25, 65-78 (2007).
757	Not relevant	Magni, M., et al. Technical Differences over the Course of the Match: An Analysis of Three Elite Teams in the UEFA Champions League. Sports (2075-4663) 11, 46 (2023).
758	Not relevant	Mahedero, M.P., Calderón, A., Hastie, P. & Arias-Estero, J.L. Grouping Students by Skill Level in Mini-Volleyball: Effect on Game Performance and Knowledge in Sport Education. Perceptual & Motor Skills 128, 1851-1871 (2021).
759	Not relevant	Mahmood-ul-Hassan, S., Tabassum, M.F., Ahmad, J. & Khan, S. THE IMPLEMENTATION OF CONCEPTUAL METAPHOR THEORY IN THE CONTEXT OF FOOTBALL AND LINGUISTICS. Shield: Research Journal of Physical Education & Sports Science 12, 67-83 (2017).
760	Not relevant	Malaguti, M., Scarpino, M., Angeloni, C. & Hrelia, S. The use of dietary supplements among soccer referees: How much do they know? Journal of Human Sport & Exercise 14, 856-865 (2019).
761	Not physical or physiological variables	Mallo J, Frutos PG, Juárez D, Navarro E. Effect of positioning on the accuracy of decision making of association football top-class referees and assistant referees during competitive matches
762	Not physical or physiological variables	Mallo, J. & Aranda, J.M.G. & Navarro, Enrique.Evaluation of the physical match performance of association football referees and assistant referees

S1 File. Studies Identification

763	Not relevant	Mallo, J., Aranda, J.M.G. & Navarro, E. Evaluation of the physical match performance of association football referees and assistant referees.
764	Not English	Mallo, J., Aranda, J.M.G. & Navarro, E.Determining the definition and components of successful soccer referee performance
765	Not relevant	Mallo, J., Cala, A., González Frutos, P. & Navarro, E. Match activities of top-class female soccer assistant referees in relation to the offside line. "Eur J Sport Sci,[2020#5Year]IF->2.781#3.228" 10, 371-376 (2010).
766	Not relevant	Mallo, J., Frutos, P.G., Juárez, D. & Navarro, E. Effect of positioning on the accuracy of decision making of association football top-class referees and assistant referees during competitive matches. "J Sports Sci,[2020#5Year]IF->2.597#3.060" 30, 1437-1445 (2012).
767	Not relevant	Mallo, J., García Aranda, J.M. & Navarro, E. Physical match performance of soccfr referees ans assistant referees in relation to the level of competition.
768	Not relevant	Mallo, J., Navarro, E., Aranda, J.G. & Helsen, W. Activity profile of top-class association football referees in relation to fitness-test performance and match standard. "J Sports Sci,[2020#5Year]IF->2.597#3.060" 27, 9-17 (2009).
769	Not relevant	Mallo, J., Navarro, E., García-Aranda, J.M., Gilis, B. & Helsen, W. Analysis of the kinematical demands imposed on top-class assistant referees during competitive soccer matches.
770	Not comparisons in the time phase	Mallo, J., Veiga, S., López de Subijana, C. & Navarro, E. Activity profile of top-class female soccer refereeing in relation to the position of the ball. "J Sci Med Sport,[2020#5Year]IF->3.607#4.332" 13, 129-132 (2010).
771	Not relevant	Mallo, J; Cala, A; Frutos, P; Navarro, E.Match activities of top-class female soccer assistant referees in relation to the offside line
772	Not relevant	Mallo, J; Frutos, PG; Juárez, D; Navarro, E.Effect of positioning on the accuracy of decision making of association football top-class referees and assistant referees during competitive matches

S1 File. Studies Identification

773	Not relevant	Mallo, J; Veiga, S; de Subijana, CL; Navarro, E.Activity profile of top-class female soccer refereeing in relation to the position of the ball
774	Not physical or physiological variables	Mallo, Javier & Cala, Antonio & González-Frutos, Pablo & Navarro, Enrique.Match activities of top-class female soccer assistant referees in relation to the offside line
775	Not relevant	Małolepszy, E. & Drozdek-Małolepsza, T. Organisational development of sport in the Volhynian province during the Second Polish Republic. Studies in Sport Humanities, 9-16 (2019).
776	Not relevant	Man, F. & Stuchlikova, I. Trait-State Anxiety, Worry, Emotionality, and Self-Confidence in Top-Level Soccer Players. "Sport Psychol,[2020#5Year]IF->1.515#2.080" 9, 212-224 (1995).
777	Not relevant	Managing Globalization: The Case of Elite Basketball Policy in the People's Republic of China. "J Sport Manage,[2020#5Year]IF->2.359#2.877" 25, 408-422 (2011).
778	Not relevant	Mangan, S., Collins, K., Burns, C. & O'Neill, C. A tactical periodisation model for Gaelic football. International Journal of Sports Science & Coaching 17, 208-219 (2022).
779	Not relevant	Mangan, S., Collins, K., Burns, C. & O'Neill, C. An investigation into the physical, physiological and technical demands of small sided games using varying pitch dimensions in Gaelic football. "International Journal Of Performance Analysis In Sport,[2020#5Year]IF->1.518#1.707" 19, 971-984 (2019).
780	Not relevant	Mangan, S., Collins, K., Burns, C. & O'Neill, C. The positional technical and running performance of sub-elite Gaelic football. Science & Medicine in Football 4, 182-191 (2020).
781	Not relevant	Manso-Lorenzo, V., Evangelio, C., Ruiz-Tendero, G. & GonzÁLez-VíLlora, S. Teacher or student-centred model? Step-by-step analysis of basic psychological needs of a new sport - goubak. Journal of Physical Education & Sport 20, 3212-3221 (2020).
782	Not relevant	Manuel Bermejo, J. THE ROLE OF THE COACH AMONG YOUNG PLAYERS OF THE BALEARIC ISLANDS. Journal of Physical Education & Health 8, 25-31 (2019).

S1 File. Studies Identification

783	Not comparisons in the time phase	Maoxiang Geng.Research on the physical condition of the national referee
784	Not relevant	Maranhão, T.J. Let the Aryanists know! Brazilian race and nation in the 1938 France World Cup. Soccer & Society 20, 912-922 (2019).
785	Not relevant	Mariante Neto, F.P., Giordani Vasques, D. & Paulo Stigger, M. "IF YOU LOSE BUT YOU PUT UP A GOOD SHOW, YOU'LL FIGHT AGAIN!" - MMA AND THE CONCEPT OF SPORT. Movimento (0104754X) 27, 1-13 (2021).
786	Not relevant	Marques Inchauspe, R., Morales Barbian, P., Aguiar Lélis, G., Renata de Almeida, F. & Vaquera, A. Physiological stress of basketball referees during a national competition. Brazilian Journal of Kineanthropometry & Human Performance 23, 1-5 (2021).
787	Not relevant	Marshall, S., McNeil, N., Seal, E.-L. & Nicholson, M. The "Boys' Club", sexual harassment, and discriminatory resourcing: An exploration of the barriers faced by women sport officials in Australian basketball. "International Review For The Sociology Of Sport,[2020#5Year]IF->2.019#1.972" 58, 971-995 (2023).
788	Not relevant	Martin, A.M., Ryu, D., Jackson, R.C. & Mann, D.L. Expert Views on Evidence-Based Classification for Goalball: A Delphi Study. "Adapt Phys Act Quart,[2020#5Year]IF->1.462#2.336" 40, 257-279 (2023).
789	Not relevant	Martin, E. & Beckham, G. Force production during the sustained phase of Rugby scrums: a systematic literature review. BMC Sports Science, Medicine & Rehabilitation 12, 1-18 (2020).
790	Not relevant	Martin, J., Smith, N.C., Tolfrey, K. & Jones, A.M. Activity analysis of English Premiership rugby football union refereeing. "Ergonomics,[2020#5Year]IF->2.190#2.548" 44, 1069-1075 (2001).
791	Not relevant	Martín-Castellanos, A., et al. How do the football teams play in LaLiga? Analysis and comparison of playing styles according to the outcome. "International Journal Of Performance Analysis In Sport,[2020#5Year]IF->1.518#1.707" 24, 18-30 (2024).

S1 File. Studies Identification

792	Not relevant	Martínez de Ojeda, D., Puente-Maxera, F. & Méndez-Giménez, A. MOTIVATIONAL AND SOCIAL EFFECTS OF A MULTIANNUAL SPORT EDUCATION PROGRAM. International Journal of Medicine & Science of Physical Activity & Sport / Revista Internacional de Medicina y Ciencias de la Actividad Física y del Deporte 21, 29-46 (2021).
793	Not relevant	Martínez Reñón, C. & Collado, P.S. An assessment of the nutritional intake of soccer referees. "J Int Soc Sports Nutr,[2020#5Year]IF->5.068#4.567" 12, 1-7 (2015).
794	Not relevant	Martínez-García, M.-L. & Rodríguez-Menéndez, C. 'I can try it': negotiating masculinity through football in the playground. Sport, Education & Society 25, 199-212 (2020).
795	Not relevant	MartÍNez-Moreno, A., IbÁÑEz-PÉRez, R. & SÁNchez-Roca, C. Leadership, stress and burnout among basketball referees. Journal of Human Sport & Exercise 16, 84-96 (2021).
796	Selected	Martínez-Torremocha et al.2022Physical demands on professional Spanish football referees during matches
797	Not relevant	Martinez-Torremocha, G., et al. How Do Technical and Tactical Demands of Football Matches Affect the Physical Performance of Elite Football Referees? Exploring the Role of Experience.
798	Not relevant	Martinez-Torremocha, G., et al. Physical Demands in the Worst-Case Scenarios of Elite Futsal Referees Using a Local Positioning System.
799	Not relevant	Martínez-Torremocha, G., et al. Physical demands on professional Spanish football referees during matches. Science & Medicine in Football 7, 139-145 (2023).
800	No full text	Martínez-Torremocha, G., Martin-Sanchez, M. L., Garcia-Unanue, J., Felipe, J. L., Moreno-Pérez, V., Paredes-Herná ndez, V., … Sanchez-Sanchez, J.Physical demands on professional Spanish football referees during matches
801	Review	Martinho, D.V., Field, A., Rebelo, A., Gouveia, É.R. & Sarmento, H. A Systematic Review of the Physical, Physiological, Nutritional and Anthropometric Profiles of Soccer Referees.
802	Not relevant	Martin-Sanchez, M.L., et al. Physical demands in Spanish male and female elite football referees during the competition: a prospective observational study. Science & Medicine in Football 6, 566-571 (2022).

S1 File. Studies Identification

803	Not relevant	Masaki, N., Shuta, O., Atsushi, I., Shutaro, S. & Sumi, Y. Daytime Napping Benefits Passing Performance and Scanning Activity in Elite Soccer Players. Journal of Sports Science & Medicine 22, 75-83 (2023).
804	Conference Paper	Masal, E; Onder, I; Besoluk, S; Caliskan, H; Demirhan, E.The effect of morphological characteristics on the physical and physiological performance of Turkish soccer referees and assistant referees
805	Not relevant	Mascarenhas, D.R.D., Birtwhistle, J. & Martindale, A. First-person video recordings with eye tracking glasses and cognitive task analysis as a framework for referee decision training.
806	Not relevant	Mascarenhas, D.R.D., Collins, D., Mortimer, P.W. & Morris, B. Training Accurate and Coherent Decision Making in Rugby Union Referees. "Sport Psychol,[2020#5Year]IF->1.515#2.080" 19, 131 (2005).
807	Not relevant	Mascherini, G., et al. Eating Habits and Body Composition of International Elite Soccer Referees. "Journal Of Human Kinetics,[2020#5Year]IF->1.664#1.886" 71, 145-153 (2020).
808	Not relevant	Maslennikov, A., Soloviev, M., Vakalova, L., Zaiko, D. & Dmitriev, I. Improvement of physical condition of football referees by athletics. Journal of Physical Education & Sport 19, 8-15 (2019).
809	Not relevant	Mather, G. & Breivik, S. Is the perception of intent by association football officials influenced by video playback speed? R Soc Open Sci 7, 192026 (2020).
810	Not relevant	Matković, A., Rupčić, T. & Knjaz, D. PHYSIOLOGICAL LOAD OF REFEREES DURING BASKETBALL GAMES. "Kinesiology,[2020#5Year]IF->1.225#1.316" 46, 258-265 (2014).
811	Not high-level matches	Matthew et al.,The impact of specific high-intensity training sessions on football referees' fitness levels
812	Not comparisons in the time phase	Matthew Weston.Intensities of exercise during match-play in FA Premier League referees and players
813	Review	Matute-Llorente, A; Sanchez-Sanchez, J; Castagna, C; Casajus, JA

S1 File. Studies Identification

814	Not relevant	Maughan, R.J., Watson, P., Evans, G.H., Broad, N. & Shirreffs, S.M. Water Balance and Salt Losses in Competitive Football. International Journal of Sport Nutrition & Exercise Metabolism 17, 583-594 (2007).
815	Not relevant	Mazaheri, R., Halabchi, F., Barghi, T.S. & Mansournia, M.A. Cardiorespiratory fitness and body composition of soccer referees; do these correlate with proper performance?
816	Not relevant	Mazaheri, R., Halabchi, F., Barghi, T.S. & Mansournia, M.A. Cardiorespiratory Fitness and Body Composition of Soccer Referees; Do These Correlate With Proper Performance? Asian Journal of Sports Medicine 7, 1-5 (2016).
817	Not relevant	McEwan, G.P., Unnithan, V.B., Easton, C. & Arthur, R. Training practices and perceptions of soccer officials: Insights from the Referee Training Activity Questionnaire. International Journal of Sports Science & Coaching 18, 1173-1189 (2023).
818	Not relevant	McEwan, G.P., Unnithan, V.B., Easton, C., Glover, A.J. & Arthur, R. Validity and reliability of the physiological and perceptual responses elicited during a novel treadmill-based Soccer Referee Simulation (SRS). Sport Sciences for Health 19, 1153-1161 (2023).
819	Not relevant	Mcewan, GP; Unnithan, VB; Easton, C; Glover, AJ; Arthur, R.Decision-making accuracy of soccer referees in relation to markers of internal and external load
820	Not relevant	McFarland, A. La Furia of '29: international friendlies and triumph during historical transition in Spain. Soccer & Society 23, 1130-1142 (2022).
821	Not relevant	McGuckin, M.E.C., Turnnidge, J., Bruner, M.W., Lefebvre, J.S. & Côté, J. Exploring youth sport coaches' perceptions of intended outcomes of leadership behaviours. International Journal of Sports Science & Coaching 17, 463-476 (2022).
822	Not relevant	McKay, J. & O'Connor, D. Practicing Unstructured Play in Team Ball Sports: A Rugby Union Example. International Sport Coaching Journal 5, 273-280 (2018).
823	Not relevant	McLaughlin, D. Physical Activity Time and Being a Good Sport. Teaching Elementary Physical Education 17, 51-54 (2006).

824	Not relevant	McNamee, S., Martin, D. & Bradley, J. A transition game? A comparative analysis of possession usage following turnovers in elite Gaelic football. "International Journal Of Performance Analysis In Sport,[2020#5Year]IF->1.518#1.707" 23, 441-457 (2023).
825	Not relevant	Meckel, Y., Balikin, K. & Eliakim, A. Pre- and mid-season repeated sprint ability of soccer referees from the first and second divisions. International Journal of Sports Science & Coaching 15, 82-90 (2020).
826	Not relevant	Mendes, S., Oliveira, E.P., Monteiro, D. & Travassos, B. The Portuguese referee performance model. International Journal of Sports Science & Coaching 16, 1117-1125 (2021).
827	Not relevant	Mendes, S., Travassos, B. & Oliveira, E.P. Career development and perception of factors to the excellence of the football referee in Portugal.
828	Not relevant	Mendes, S., Travassos, B. & Patrícia Oliveira, E. Desenvolvimento de carreiras e perceção de fatores para a excelência do árbitro de futebol em Portugal. Retos: Nuevas Perspectivas de Educación Física, Deporte y Recreación 37, 694-701 (2020).
829	Not relevant	Mendes, S; Oliveira, EP; Monteiro, D; Travassos, B.The Portuguese referee performance model
830	Not relevant	Mendes, S; Travassos, B; Oliveira, EP.Career development and perception of factors to the excellence of the football referee in Portugal
831	Not relevant	Menuchi, M.R.T.P., Moro, A.R.P., Ambrósio, P.E., Pariente, C.A.B. & Araújo, D. Effects of Spatiotemporal Constraints and Age on the Interactions of Soccer Players when Competing for Ball Possession. Journal of Sports Science & Medicine 17, 379-391 (2018).
832	Not relevant	Mitchell, S. & Tierney, G.J. Sanctioning of breakdown infringements during the knockout stage of the 2019 rugby world cup. International Journal of Sports Science & Coaching 16, 407-414 (2021).
833	Not relevant	Mitrotasios, M., Gonzalez-Rodenas, J., Armatas, V. & Aranda, R. The creation of goal scoring opportunities in professional soccer. Tactical differences between Spanish La Liga, English Premier League, German Bundesliga and Italian Serie A. "International Journal Of Performance Analysis In Sport,[2020#5Year]IF->1.518#1.707" 19, 452-465 (2019).

S1 File. Studies Identification

834	Not relevant	Mitten, M.J. How is the Integrity of Sport Protected in the United States? Texas Review of Entertainment & Sports Law 19, 89-105 (2019).
835	Not relevant	Mkumbuzi, N.S., Dlamini, S.B., Chibhabha, F. & Govere, F.M. Injury, illness, and medication use surveillance during the 2020 COSAFA Women's championship: a prospective cohort study of football players from Southern Africa. Science & Medicine in Football 7, 74-80 (2023).
836	Not relevant	Mockford, S. & Pearey, A. 20 WAYS TO SHAKE UP RUGBY. Rugby World, 37-41 (2021).
837	Not relevant	Mockford, S., Dymock, A., Pearey, A., Palmer, M. & Eddison, P. WORLD CUP LOWDOWN. Rugby World, 40-77 (2022).
838	Not relevant	Modolo, F., RomÁRio Dos Santos, W., Resina De Oliveira Campos, R., Pereira Morato, M. & Pombo Menezes, R. KEY FEATURES OF BEACH HANDBALL SELF-ORGANIZATION: COMPARISON BETWEEN GENDERS. Human Movement 23, 72-80 (2022).
839	Not relevant	Moen, C., Andersen, T.E., Clarsen, B., Madsen-Kaarød, G. & Dalen-Lorentsen, T. Prevalence and burden of health problems in top-level football referees. Science & Medicine in Football 7, 131-138 (2023).
840	Not relevant	Mohamed Shapie, M.N., Oliver, J., O'Donoghue, P. & Tong, R. Activity profile during action time in national silat competition. Journal of Combat Sports & Martial Arts 4, 81-85 (2013).
841	Not relevant	Monea, D; Prodan, R; Grosu, VT.CONTINUOUS EDUCATION AND TRAINING OF ADULT SPORTSMEN - STUDY CASE: THE FOOTBALL REFEREES
842	Not relevant	Monsma, E.V., Peters, D. & Smith, R. The Art and Science of Teaching Imagery: Applications for Soccer. Teaching Elementary Physical Education 14, 17-19 (2003).
843	Not relevant	Morais, J.E., et al. Analysis of the physiological response in junior tennis players during short-term recovery: Understanding the magnitude of recovery until and after the 25 seconds rule. International Journal of Sports Science & Coaching 18, 1208-1216 (2023).

S1 File. Studies Identification

844	Not relevant	Morales, C.A. A mathematics-based new penalty area in football: tackling diving. "J Sports Sci,[2020#5Year]IF->2.597#3.060" 34, 2233-2237 (2016).
845	Not relevant	Moreno, D.R., Murias, T.F. & Barbajero, J.E. Training soccer referees and assistant referees from the flipped learning approach.
846	Not physical or physiological variables	Moreno-Perez V, Courel-Ibáñez J, Del Coso J, Sánchez-Sánchez J.The Effects of Match Congestion on Physical Performance in Football Referees
847	Not relevant	Moreno-pérez, V., et al. Eccentric hamstring muscle strength during home confinement due to the covid-19 pandemic, and football competition resumption in professional football referees: A prospective observational study.
848	Not relevant	Moreno-Perez, V., et al. Impact of COVID-19 lockdown on match activity and physical performance in professional football referees.
849	Not relevant	Moreno-Perez, V; Courel-Ibáñez, J; Del Coso, J; Sánchez-Sánchez, J
850	Not relevant	Morris, G. & O'Connor, D. Key attributes of expert NRL referees. "J Sports Sci,[2020#5Year]IF->2.597#3.060" 35, 852-857 (2017).
851	Conference Paper	Moshayedi, A.J., Chen, Z., Liao, L. & Li, S. Kinect Based Virtual Referee for Table Tennis Game: TTV (Table Tennis Var System).
852	Not relevant	Motor Learning and Control. Journal of Sport & Exercise Psychology 31, S45-S107 (2009).
853	Not relevant	Mowling, C.M., Brock, S.J. & Hastie, P.A. Fourth Grade Students' Drawing Interpretations of a Sport Education Soccer Unit. "J Teach Phys Educ,[2020#5Year]IF->1.845#2.490" 25, 9-35 (2006).

S1 File. Studies Identification

854	Not relevant	Moya-Vergara, F., et al. Evaluation of visual-motor reaction time and quality of response in rugby sevens players after the application of a neurocognitive training programme. "International Journal Of Performance Analysis In Sport,[2020#5Year]IF->1.518#1.707" 19, 1038-1051 (2019).
855	Not relevant	Mrković, R., Talović, M., Jeleškovic, E., Alić, H. & Bajramović, I. Correlation of refereeing motor characteristics and success at football referees in the sarajevo canton. Homo Sporticus 11, 19-22 (2009).
856	Not relevant	Mthombeni, S., Coopoo, Y. & Noorbhai, H. Perceptions of current support systems leading to international sporting success for South Africans. International Sports Studies 44, 22-38 (2022).
857	Not relevant	Mughal, H., Iqbal, M. & Ahmed, A.S. STRATEGIC PLANNING FOR THE IMPROVEMENT OF SPORTS AT GRASS ROOT LEVEL IN PUNJAB (HOCKEY, CRICKET AND BASKETBALL). Shield: Research Journal of Physical Education & Sports Science 15, 34-47 (2020).
858	Not relevant	Muniroglu, S. & Subak, E. Carrying a flag decreases the sprint performances of the soccer assistant referees.
859	Not relevant	MÜNİRoĞLu, S. & Subak, E. FUTBOL HAKEMLERİNİN SPRİNT PERFORMANSLARININ ÇABUKLUK-ÇEVİKLİK PERFORMANSLARIYLA İLİŞKİSİNİN İNCELENMESİ. Congress Papers of The Association of Sports Sciences, 128-131 (2019).
860	Not relevant	Müniroglu, S. The Relation Between Heart Rate and Running Distances of Football Referees During the Matches. International Journal of Applied Sports Sciences 19, 7-15 (2007).
861	Not relevant	Muñoz-Arjona, C. & Castillo-Rodríguez, A. Attitude vs. Aptitude. Effect of psychological responses on soccer Referees.
862	Not relevant	Muñoz-Arjona, C., Fernandes, B.R., Dos Santos, T.D. & Castillo-Rodríguez, A. Do psychological responses and experience influence the physical performance of soccer referees?
863	Not relevant	Muñoz-Arjona, C., Onetti-Onetti, W. & Castillo-Rodríguez, A. ¿Influyen las características corporales y la adherencia a la dieta mediterránea en la categoría arbitral? Cultura, Ciencia y Deporte 16, 529-536 (2021).

S1 File. Studies Identification

864	Not relevant	Muñoz-Arjona, C; Castillo-Rodríguez, A.Attitude vs. Aptitude. Effect of psychological responses on soccer Referees
865	Not relevant	Muñoz-Arjona, C; Fernandes, BR; Dos Santos, TD; Castillo-Rodríguez, A.Do psychological responses and experience influence the physical performance of soccer referees?
866	Not relevant	Muñoz-Arjona, C; Onetti-Onetti, W; Castillo-Rodríguez, A.Does the arbitral category influence in the adherence to the mediterranean diet and body composition?
867	Not relevant	Muscella, A., et al. Effects of training on plasmatic cortisol and testosterone in football female referees.
868	Not relevant	Muscella, A., Stefàno, E. & Marsigliante, S. The effects of training on hormonal concentrations and physical performance of football referees. Physiol Rep 9, e14740 (2021).
869	Not relevant	Muscella, A., Stefàno, E., Di Maglie, A. & Marsigliante, S. Referees' physical performance over a soccer season. Sport Sciences for Health 16, 765-773 (2020).
870	Not relevant	Muscella, A; Stefàno, E; Santo Marsigliante.The effects of training on hormonal concentrations and physical performance of football referees
871	Not relevant	Myers, N.D., Feltz, D.L., Guillén, F. & Dithurbide, L. Development of, and Initial Validity Evidence for, the Referee Self-Efficacy Scale: A Multistudy Report. Journal of Sport & Exercise Psychology 34, 737-765 (2012).
872	Not relevant	Myers, N.L., Kibler, W.B., Axtell, A.H. & Uhl, T.L. The Sony Smart Tennis Sensor accurately measures external workload in junior tennis players. International Journal of Sports Science & Coaching 14, 24-31 (2019).
873	Not relevant	Nabli, M.A., et al. Fitness Field Tests' Correlation With Game Performance in U-19-Category Basketball Referees. International Journal of Sports Physiology & Performance 11, 1005-1011 (2016).
874	Not relevant	Nahidi, N., Saemi, E., Doustan, M., Aronson, J. & Laurin, R. The Effect of Gender Stereotype Threat and Conceptions of Ability on Motor Learning and Working Memory. Journal of Motor Learning & Development 11, 338-358 (2023).

S1 File. Studies Identification

875	Not relevant	Naik, B.T., Hashmi, M.F., Geem, Z.W. & Bokde, N.D. DeepPlayer-Track: Player and Referee Tracking With Jersey Color Recognition in Soccer.
876	Not relevant	Nam, B.H., Marshall, R.C., Love, A., Graham, J. & Lim, S. Fostering Global Sport Leadership: A Partnership between a Korean Sport Organization and a U.S. University. Journal of Global Sport Management 7, 199-225 (2022).
877	Not relevant	Naraine, M.L. The Blockchain Phenomenon: Conceptualizing Decentralized Networks and the Value Proposition to the Sport Industry. International Journal of Sport Communication 12, 313-335 (2019).
878	Not relevant	Naydenova, K. & Ignatov, G. Study of the functional load of elite football assistant referees in the Republic of Bulgaria during official matches.
879	Conference Paper	Neeley, KA.A Decision Support System for the Professional Soccer Referee in Time-Sensitive Operations
880	Not relevant	Neill, K. The youdan and Cromwell cups: Sheffield football's knockout trophies. Soccer & Society 24, 62-73 (2023).
881	Not relevant	Nejić, D., Trajković, N., Nejić, K., Milenkoski, J. & Misovski, A. EFFECTS OF BEACH VOLLEYBALL TRAINING ON PHYSICAL PERFORMANCE IN YOUNG INDOOR VOLLEYBALL PLAYERS. Research in Physical Education, Sport & Health 5, 49-55 (2016).
882	Not relevant	Nenad, T., Srđan, M. & Vukašin, M. ACTIVITIES OF FOOTBALL REFEREES DURING GAMES OF DIFFERENT COMPETITION LEVELS – A CASE STUDY.
883	Not relevant	Nevill, A.M., Newell, S.M. & Gale, S. Factors associated with home advantage in english and scottish soccer matches.
884	Not relevant	Nichol, A.J., Hall, E.T., Vickery, W. & Hayes, P.R. Examining the Relationships Between Coaching Practice and Athlete "Outcomes": A Systematic Review and Critical Realist Critique. International Sport Coaching Journal 6, 13-29 (2019).

S1 File. Studies Identification

885	Not relevant	Nunes, R.F.H., et al. Recovery following Rugby Union matches: effects of cold water immersion on markers of fatigue and damage. Applied Physiology, Nutrition & Metabolism 44, 546-556 (2019).
886	Not relevant	O Brien, P., Martin, D. & Bradley, J. Differences in performance indicators between winners and losers in senior inter county hurling championship. "International Journal Of Performance Analysis In Sport,[2020#5Year]IF->1.518#1.707" 21, 630-640 (2021).
887	Not relevant	O'Brien, K.A. & Rynne, S.B. Seen but not heard: missing the mark in conceptualizing high performance officiating. "Sport in Society,[2020#5Year]IF->0.939#Not Available" 24, 1110-1121 (2021).
888	Not relevant	O'Brien, K.A., Rynne, S.B. & Mallett, C.J. The development of craft in Australian National Rugby League referees. Sport, Education & Society 28, 420-433 (2023).
889	Not relevant	Oguz, Y., et al. EVALUATION OF SUBJECTIVE HEARING IN FOOTBALL REFEREES. Ovidius University Annals, Series Physical Education & Sport/Science, Movement & Health 19, 131-135 (2019).
890	Not relevant	O'Hallarn, B., Slavich, M. & Emmons, B. "I Used to Love Scheifele:" Dominant Narratives on Reddit About the Canadian Division of the Stanley Cup Playoffs. Communication & Sport 11, 1203-1222 (2023).
891	Not comparisons in the time phase	Ohashi, J.Measuring movement speeds and distances covered during soccer match play
892	Not relevant	Oliva-Lozano, J.M., Gómez-Carmona, C.D., Pino-Ortega, J., Moreno-Pérez, V. & Rodríguez-Pérez, M.A. Match and Training High Intensity Activity-Demands Profile during a Competitive Mesocycle in Youth Elite Soccer Players. "Journal Of Human Kinetics,[2020#5Year]IF->1.664#1.886" 75, 195-205 (2021).
893	Not relevant	Oliveira, M.A.C., Dambroz, F., Santos, R. & Moniz, F. VAR implementation and soccer team performance: a comparison between the 2014 and 2018 World Cups.
894	Not relevant	Oliveira, M.C., Silva, A.I., Agresta, M.C., Barros Neto, T.L. & BrandÆo, M.F. Nível de concentração e precisão de á rbitros de futebol ao longo de uma partida. Motricidade 9, 13-22 (2013).

S1 File. Studies Identification

895	Not relevant	Omondi-Ochieng, P. Africa cup of nations: a resource-based view of football staff. Managing Sport & Leisure 24, 193-207 (2019).
896	Not relevant	Orviz-Martínez, N., Botey-Fullat, M. & Arce-García, S. Analysis of burnout and psychosocial factors in grassroot football referees.
897	Not relevant	Otte, F.W., Millar, S.-K. & Klatt, S. What do you hear? The effect of stadium noise on football players' passing performances. "Eur J Sport Sci,[2020#5Year]IF->2.781#3.228" 21, 1035-1044 (2021).
898	Not relevant	Oudejans, R.R.D., et al. How position and motion of expert assistant referees in soccer relate to the quality of their offside judgements during actual match play.
899	Not relevant	Oudejans, RRD; Bakker, FC; Verheijen, R; Gerrits, JC; Steinbrückner, M; Beek, PJ
900	Not relevant	Ouergui, I., et al. Psycho-physiological aspects of small combats in taekwondo: impact of area size and within-round sparring partners. "Biol Sport,[2020#5Year]IF->2.000#2.250" 38, 157-164 (2021).
901	Not relevant	Ouergui, I., et al. Repeated High-Intensity Technique Training and Repeated Sprint Training Elicit Similar Adjustment in Physiological Responses But Divergent Perceptual Responses and Combat-Related Performances in Adolescent Taekwondo Matches. International Journal of Sports Physiology & Performance 18, 825-832 (2023).
902	Not relevant	Owusu-Sekyere, F. & Gervis, M. In the pursuit of Mental Toughness: Is Creating Mentally Tough Players a Disguise for Emotional Abuse? International Journal of Coaching Science 10, 3-23 (2016).
903	Not high-level matches	Ozaeta E, Yanci J, Castagna C, Romaratezabala E, Castillo D.Associations between well-being state and match external and internal load in amateur referees
904	Not relevant	Ozaeta, E., Fernández-Lasa, U., Martínez-Aldama, I., Cayero, R. & Castillo, D. Match Physical and Physiological Response of Amateur Soccer Referees: A Comparison between Halves and Match Periods.

S1 File. Studies Identification

905	Not relevant	Ozaeta, E., Yanci, J., Castagna, C., Romaratezabala, E. & Castillo, D. Associations between well-being state and match external and internal load in amateur referees.
906	Not relevant	Özcan, İ., Eniseler, N. & Şahan, Ç. EFFECTS OF SMALL-SIDED GAMES AND CONVENTIONAL AEROBIC INTERVAL TRAINING ON VARIOUS PHYSIOLOGICAL CHARACTERISTICS AND DEFENSIVE AND OFFENSIVE SKILLS USED IN SOCCER. "Kinesiology,[2020#5Year]IF->1.225#1.316" 50, 104-111 (2018).
907	Not relevant	ÖZdamar, E., Hazir Aytar, S. & KİN İŞLer, A. FUTBOL İL HAKEMLERİNİN MÜSABAKA SIRASINDA ORTAYA KOYDUKLARI FİZYOLOJİK YÜKÜN LİG DÜZEYİNE GÖRE İNCELENMESİ. SPORMETRE: The Journal of Physical Education & Sport Sciences / Beden Eğitimi ve Spor Bilimleri Dergisi 19, 29-38 (2021).
908	Not relevant	Paes, M.R. & Fernandez, R. Evaluation of energy expenditure in forward and backward movements performed by soccer referees.
909	Not relevant	Paes, M.R. & Fernandez, R. Use of a modified Yo-Yo intermittent endurance level 2 test for evaluation of field soccer referees.
910	Not relevant	Paes, M.R., Fernandez, R. & da Silva, A.I. Injuries to football (soccer) referees during matches, training and physical tests. International SportMed Journal 12, 74-84 (2011).
911	Not relevant	Paes, MR; Fernandez, R.Evaluation of energy expenditure in forward and backward movements performed by soccer referees
912	Not relevant	Paes, MR; Fernandez, R.Use of a modified Yo-Yo intermittent endurance level 2 test for evaluation of field soccer referees
913	Not relevant	Pakaslahti, A. Betterness, injustice and failed athletic contests. "J Phil Sport,[2020#5Year]IF->0.867#0.814" 43, 281-293 (2016).
914	Not relevant	Palermi, S., et al. Effectiveness of the FIFA11+ Referees Injury Prevention Program in improving athletic performance in male professional soccer referees.

S1 File. Studies Identification

915	Not relevant	Palermi, S; Vecchiato, M; Spinelli, A; Gallinoro, CM; Annarumma, G; DI Gregorio, A; Nuccio, F; DI Salvatore, M; Cozzolino, A; Tuzi, M; Sirico, F
916	Not relevant	Pan, P., Li, F., Han, B., Yuan, B. & Liu, T. Exploring the impact of professional soccer substitute players on physical and technical performance. BMC Sports Science, Medicine & Rehabilitation 15, 1-11 (2023).
917	Not relevant	Paradis, K., Larkin, P. & O'Connor, D. The effects of physical exertion on decision-making performance of Australian football umpires. "J Sports Sci,[2020#5Year]IF->2.597#3.060" 34, 1535-1541 (2016).
918	Not relevant	Parmar, N., James, N., Hearne, G. & Jones, B. Using principal component analysis to develop performance indicators in professional rugby league. "International Journal Of Performance Analysis In Sport,[2020#5Year]IF->1.518#1.707" 18, 938-949 (2018).
919	Not relevant	ParniČAn, S., PerÁČEk, P. & TÓTh, I. Differentiation between checks to the head or neck as a primary cause of mild traumatic brain injury in ice hockey in Slovakia. Journal of Physical Education & Sport 20, 3445-3451 (2020).
920	Not relevant	Parpa, K. & Michaelides, M.A. Maximal Aerobic Power Using the Modified Heck Protocol: Prediction Models.
921	Not relevant	Parry, J. Sport and Olympism: Universals and Multiculturalism. "J Phil Sport,[2020#5Year]IF->0.867#0.814" 33, 188-204 (2006).
922	Not relevant	Parry, K., et al. Masculinities, Media and the Rugby Mind: An Analysis of Stakeholder Views on the Relationship Between Rugby Union, the Media, Masculine-Influenced Views on Injury, and Concussion. Communication & Sport 10, 564-586 (2022).
923	Not relevant	Passero, J.G., Barreira, J., Calderani Junior, A. & Galatti, L.R. Gender (in)equality: a longitudinal analysis of women's participation in coaching and referee positions in the Brazilian Women's Basketball League (2010-2017). Cuadernos de Psicología del Deporte 19, 252-261 (2019).
924	Not relevant	Paul, G. CROSSING THE LINE. NZ Rugby World, 74-81 (2020).

S1 File. Studies Identification

925	Not relevant	Paul, L., et al. More than rugby: A scoping review of coaches in rugby. International Journal of Sports Science & Coaching 18, 2277-2291 (2023).
926	Not relevant	Peric, A., Markovič, G., Bradic, A. & Dizdar, D. The Weighted Expert System for the Evaluation of Actual Quality of Top-level Soccer Players. Homo Sporticus 15, 10-19 (2013).
927	Not relevant	Perlmuter, R.E., Richards, F.A., Dreimiller, D., Dreimiller, J. & Hoyt, C.L. Sports officiating: career handbook, (LR Publishing Company, 1997).
928	Not relevant	Pernigoni, M., et al. Is foam rolling as effective as its popularity suggests? A randomised crossover study exploring post-match recovery in female basketball. "J Sports Sci,[2020#5Year]IF->2.597#3.060" 41, 1718-1725 (2023).
929	Not relevant	Perreau-Niel, A. & Erard, C. French football referees: an exploratory study of the conditions of access and employment for referees in terms of level and gender. Soccer & Society 16, 1-16 (2015).
930	Not relevant	Perroni, F; Amatori, S; Corsi, L; Bensi, R; Guidetti, L; Baldari, C; Rocchi, MBL; Castagna, C; Gobbi, E; Sisti, D; Buzzachera, CF
931	Not relevant	Persson, E., Andersson, M. & Blomqvist, S. Differences in Physical Demands Among Offensive and Defensive Players in Elite Men Bandy. Research Quarterly for Exercise & Sport 92, 805-812 (2021).
932	Selected	Peter Krustrup et al. 2009Activity profile and physical demands of football referees and assistant referees in international games
933	Not relevant	Petri, C., et al. Body fat assessment in international elite soccer referees.
934	Not relevant	Philippe, F.L., Vallerand, R.J., Andrianarisoa, J. & Brunel, P. Passion in Referees: Examining Their Affective and Cognitive Experiences in Sport Situations. Journal of Sport & Exercise Psychology 31, 77-96 (2009).
935	Not relevant	Phillips, J. It's official. Women's Sports & Fitness 18, 66 (1996).

936	Not relevant	Pho, A. & White, B.A. A critical note on a purported disanalogy between cycling and mixed martial arts. "J Phil Sport,[2020#5Year]IF->0.867#0.814" 49, 177-194 (2022).
937	Not relevant	Pic Aguilar, M. Temporal consistencies in two champion teams of European football? Retos: Nuevas Perspectivas de Educación Física, Deporte y Recreación 34, 94-99 (2018).
938	Not relevant	Picazo-Tadeo, A.J., González-Gómez, F. & Guardiola, J. Does the crowd matter in refereeing decisions? Evidence from Spanish soccer. International Journal of Sport & Exercise Psychology 15, 447-459 (2017).
939	Not relevant	Pietraszewski, P., et al. The Elements of Executive Attention in Top Soccer Referees and Assistant Referees. "Journal Of Human Kinetics,[2020#5Year]IF->1.664#1.886" 40, 235-243 (2014).
940	Not relevant	Pietraszewski, P; Maszczyk, A; Roczniok, R; Golas, A; Stanula, A.Differentiation of Perceptual Processes in Elite and Assistant Soccer Referees
941	Review	Pina, JAE; Passos, A; Araújo, D; Maynard, MT.Football refereeing: An integrative review
942	Not relevant	Ping Chong, Y., Yeo, E.Q.Y., Probert, J., Sim, S.H.S. & Sirisena, D. A Systematic Review and Qualitative Analysis of Concussion Knowledge amongst Sports Coaches and Match Officials. Journal of Sports Science & Medicine 19, 65-77 (2020).
943	Not relevant	Pinot, J. & Grappe, F. A six-year monitoring case study of a top-10 cycling Grand Tour finisher. "J Sports Sci,[2020#5Year]IF->2.597#3.060" 33, 907-914 (2015).
944	Not relevant	Pitassi, C. & Lacerda, L.R.d. Technological capability of doping control laboratories: a metric proposal. International Journal of Sport Policy & Politics 11, 539-557 (2019).
945	Not relevant	Pizzera, A. & Raab, M. Does Motor or Visual Experience Enhance the Detection of Deceptive Movements in Football? International Journal of Sports Science & Coaching 7, 269-284 (2012).
946	Not relevant	Pizzera, A., Laborde, S., Lahey, J. & Wahl, P. Influence of physical and psychological stress on decision-making performance of soccer referees. "J Sports Sci,[2020#5Year]IF->2.597#3.060" 40, 2037-2046 (2022).

S1 File. Studies Identification

947	Not relevant	Pizzera, A., Marrable, J. & Raab, M. The video review system in association football: implementation and effectiveness for match officials and referee education.
948	Review	Pizzera, A; Marrable, J; Raab, M.The video review system in association football: implementation and effectiveness for match officials and referee education
949	Not relevant	Pizzi, G. & Stanger, N. Consequences of teammate moral behaviour: Linking team moral norms with cohesion and collective efficacy. International Journal of Sport & Exercise Psychology 18, 437-453 (2020).
950	Not relevant	Plakias, S., et al. Identifying playing styles of european soccer teams during the key moments of the game. Journal of Physical Education & Sport 23, 878-890 (2023).
951	Not relevant	Plakias, S., et al. Identifying Soccer Teams' Styles of Play: A Scoping and Critical Review. Journal of Functional Morphology & Kinesiology 8, 39 (2023).
952	Not relevant	Plessner, H., Ermark, F., Schütz, L.-M. & Schweizer, G. Sports Performance Judgments - An Update From a Social Cognitive Perspective. Asian Journal of Sport & Exercise Psychology 3, 13-23 (2023).
953	Not relevant	Płoszaj, K., Firek, W., Gąsior, P. & Malchrowicz-Mośko, E. Positive behavior management: Assessment of rugby referees in children sport.
954	Not relevant	Pocock, C., Bezodis, N.E., Davids, K., Wadey, R. & North, J.S. Understanding key constraints and practice design in Rugby Union place kicking: Experiential knowledge of professional kickers and experienced coaches. International Journal of Sports Science & Coaching 15, 631-641 (2020).
955	Not relevant	Pokolm, M., Kirchhain, M., Müller, D., Jordet, G. & Memmert, D. Head movement direction in football - a field study on visual scanning activity during the UEFA-U17 and -U21 European Championship 2019. "J Sports Sci,[2020#5Year]IF->2.597#3.060" 41, 695-705 (2023).
956	Not relevant	Pollock, B. Three-sided football: DIY football and social transformationalism. Sport, Education & Society 26, 1026-1040 (2021).

S1 File. Studies Identification

957	Not relevant	Ponce-Bordón, J.C., et al. The Effect of the Video Assistant Referee System Implementation on Match Physical Demands in the Spanish LaLiga. "Int J Environ Res Public Health,[2020#5Year]IF->2.849#3.127" 19(2022).
958	Not relevant	Pope, C. Pitch perfect.
959	Not relevant	Práxedes, A., González, R., del Villar, F. & Gil-Arias, A. Combining Physical Education and unstructured practice during school recess to improve the students' decision-making and execution. Retos: Nuevas Perspectivas de Educación Física, Deporte y Recreación 41, 502-511 (2021).
960	Not relevant	Preatoni, E., Cazzola, D., Stokes, K.A., England, M. & Trewartha, G. Pre-binding prior to full engagement improves loading conditions for front-row players in contested Rugby Union scrums. "Scand J Med Sci Sports,[2020#5Year]IF->3.255#3.982" 26, 1398-1407 (2016).
961	Not relevant	Preissler, A.A., et al. Acomparison of the internal and external load demands imposed on professional soccer referees in FIFA's current model of physical test in relation to games.
962	Not relevant	Preissler, A.A.B., Schons, P. & Kruel, L.F.M. Did the quarantine period of covid-19 interfere with the physical demands of central referees and assistants in professional soccer in a high level competition in Brazil?
963	Not relevant	Programme & Book of Abstracts. Journal of Human Sport & Exercise 8, S469-S552 (2013).
964	Not relevant	Puente-Maxera, F., de Ojeda, D.M., Méndez-Giménez, A., Valverde, J.J. & Jiménez-Martínez, I. Effects of a Sport Education Season of an Alternative Sport on School Climate, Emotional Intelligence, and Perceived Competence of Elementary School Students in a Culturally Diverse Context. Retos: Nuevas Perspectivas de Educación Física, Deporte y Recreación 50, 1019-1028 (2023).
965	Not relevant	Pulido, J.J., Sánchez-Oliva, D., Silva, M.N., Palmeira, A.L. & García-Calvo, T. Development and preliminary validation of the Coach Interpersonal Style Observational System. International Journal of Sports Science & Coaching 14, 471-479 (2019).

S1 File. Studies Identification

966	Not relevant	Pulling, C., Eldridge, D., Ringshall, E. & Robins, M.T. Analysis of crossing at the 2014 FIFA World Cup. "International Journal Of Performance Analysis In Sport,[2020#5Year]IF->1.518#1.707" 18, 657-677 (2018).
967	Not relevant	Qin, Y., Wu, J. & Zhang, R. Can Professional Football Players Adapt to Air Pollution? Evidence From China. "Journal Of Sports Economics,[2020#5Year]IF->1.615#1.527" 23, 277-300 (2022).
968	Conference Paper	Qing-Lai, Z., Xin-Ming, T. & Zhan-Ling, M. Design of intelligent system for soccer goal detection based on opto-electronic technology.
969	Not relevant	Quintero LÓPez, C., Gil Vera, V.D., VÁSquez LÓPez, C. & ÁLzate Jaramillo, J.C. Impact of sport on social cognition: an analysis based on structural equational models. Journal of Physical Education & Sport 20, 31-36 (2020).
970	Not relevant	Raimundo Fernandes, J., Andrade De Brito, M., Jose Brito, C., Aedo-Munoz, E. & Miarka, B. Technical-tactical actions of fighters specialized in striking, grappling, and mixed combat in the Ultimate Fighting Championship. Ido Movement for Culture. Journal of Martial Arts Anthropology 22, 23-31 (2022).
971	Not relevant	Ramachandran, P., Watts, M., Jackson, R.C., Hayes, S.J. & Causer, J. Howzat! Expert umpires use a gaze anchor to overcome the processing demands of leg before wicket decisions. "J Sports Sci,[2020#5Year]IF->2.597#3.060" 39, 1936-1943 (2021).
972	Not relevant	Ramchandani, G., Wilson, D., Millar, R. & Ashworth, B. New sports and no spectators: Japan's performance at the Tokyo 2020 Olympic Games. Managing Sport & Leisure 28, 197-208 (2023).
973	Conference Paper	Ran, G; Yun, Z; Jianming, Z; Yang, Y; Ze, L; Tao, G.Numerical Simulation of Pressure difference about Curve Ball in flight
974	Not relevant	Rance, J.J. Examination of goals scored in the 2022 World Cup football tournament in Qatar. Journal of Physical Education & Sport 23, 2951-2962 (2023).
975	Not relevant	Rawat, M. & Rajsingh, S. Athletes' right to a fair trial in 'non-analytical positive doping cases': An analysis. International Journal of Sport Policy & Politics 13, 379-391 (2021).

S1 File. Studies Identification

976	Not relevant	Raya-González, J., et al. Influence of the COVID-19 lockdown on Spanish professional soccer teams' external demands according to their on-field ranking. "Biol Sport,[2020#5Year]IF->2.000#2.250" 39, 1081-1086 (2022).
977	Not relevant	Raya-González, J., Scanlan, A.T., Sánchez-Díaz, S. & Castillo, D. Sex-based differences in the external loads imposed during an official Ultimate-Frisbee competition: A Pilot Study. European Journal of Human Movement 45, 74-81 (2020).
978	Not relevant	Read, D.B., et al. The Physical Characteristics of Specific Phases of Play During Rugby Union Match Play. International Journal of Sports Physiology & Performance 13, 1331-1336 (2018).
979	Not relevant	Rebelo, A., Silva, S., Pereira, N. & Soares, J. Stress fisico do qrbitro de futebol no jogo. Revista Portuguesa de Ciencias do Desporto 2, 24-30 (2002).
980	Not relevant	Rebelo, AN; Ascensao, AA; Magalhaes, JF; Bischoff, R; Bendiksen, M; Krustrup, P
981	Not relevant	Rebelo-Gonçalves, R., Pardal, H., Coelho, L., Antunes, R. & Amaro, N. Physiological and mechanical loads in Portuguese sub-elite football refereeing - a preliminary study. Cuadernos de Psicología del Deporte 21, 213-223 (2021).
982	Not relevant	Rebolé, M; Castillo, D; Camara, J; Yanci, J.RELATIONSHIP BETWEEN THE CARDIOVASCULAR CAPACITY AND REPEATED SPRINTS ABILITY IN HIGH-STANDARD SOCCER REFEREES
983	Not relevant	Rednall, J. SPORTSMANSHIP & GAMESMANSHIP. Bowls International, 54-56 (2023).
984	Not relevant	Regnoli, R; Rovelli, M; Gianturco, V; Pregliasco, FE; Bodini, BD; Gianturco, L
985	Not relevant	Reid, H.L. Why Olympia matters for modern sport. "J Phil Sport,[2020#5Year]IF->0.867#0.814" 44, 159-173 (2017).
986	Not relevant	Reid, K. & Dallaire, C. "Because There Are So Few of Us": The Marginalization of Female Soccer Referees in Ontario, Canada. Women in Sport & Physical Activity Journal 27, 12-20 (2019).

S1 File. Studies Identification

987	Not relevant	Reid, K. & Dallaire, C. 'I'd like to think I'm a good referee': discourses of ability and the subjectivity of the female soccer referee in Ontario (Canada). Soccer & Society 21, 762-777 (2020).
988	Not physical or physiological variables	Reilly T, Gregson W.Special populations: The referee and assistant referee
989	Book	Reilly, T. & Gregson, W. Special populations: The referee and assistant referee. "J Sports Sci,[2020#5Year]IF->2.597#3.060" 24, 795-801 (2006).
990	Conference Paper	Reilly, Thomas; Gregson, Warren.Match activities of top-class female soccer assistant referees in relation to the offside line
991	Not relevant	Rekik, R.N., et al. Mechanisms of ACL injuries in men's football: A systematic video analysis over six seasons in the Qatari professional league. "Biol Sport,[2020#5Year]IF->2.000#2.250" 40, 575-586 (2023).
992	Not relevant	Rennie, M.J., et al. Phases of match-play in professional Australian Football: Distribution of physical and technical performance. "J Sports Sci,[2020#5Year]IF->2.597#3.060" 38, 1682-1689 (2020).
993	Not relevant	Rennie, MJ; Kelly, SJ; Bush, S; Spurrs, RW; Austin, DJ; Watsford, ML
994	Not relevant	Rey, R.T., Cranmer, G.A., Browning, B. & Sanderson, J. Sport Knowledge: The Effects of Division I Coach Communication on Student-Athlete Learning Indicators. International Journal of Sport Communication 15, 33-42 (2022).
995	Not relevant	Riffi Acharki, E., Spaaij, R. & Nieuwelink, H. Social inclusion through sport? Pedagogical perspectives of Dutch youth sport coaches. Sport, Education & Society 28, 144-158 (2023).
996	Selected	Riiser et al. 2017Accelerations and high intensity running in field and assistant football referees during match play

S1 File. Studies Identification

997	Not relevant	Riiser, A., et al. Accelerations and high intensity running in field and assistant football referees during match play.
998	Not relevant	Riiser, A., et al. The Construct Validity of the CODA and Repeated Sprint Ability Tests in Football Referees.
999	Not relevant	Rimkus, L., Satkunskiene, D., Kamandulis, S. & Bruzas, V. Lower-body power in boxers is related to activity during competitive matches. "International Journal Of Performance Analysis In Sport,[2020#5Year]IF->1.518#1.707" 19, 342-352 (2019).
1000	Not relevant	Riot, C., O'Brien, W. & Minahan, C. High performance sport programs and emplaced performance capital in elite athletes from developing nations. "Sport Management Review,[2020#5Year]IF->3.337#3.761" 23, 913-924 (2020).
1001	Not relevant	Robertson, J. & Constandt, B. Moral disengagement and sport integrity: identifying and mitigating integrity breaches in sport management. "European Sport Management Quarterly,[2020#5Year]IF->1.889#2.436" 21, 714-730 (2021).
1002	Not relevant	Rocamora, I., González-Víllora, S., Fernández-Río, J. & Arias-Palencia, N.M. Physical activity levels, game performance and friendship goals using two different pedagogical models: Sport Education and Direct Instruction. Physical Education & Sport Pedagogy 24, 87-102 (2019).
1003	Review	Rodríguez, S., Rodríguez-Jaime, M.F., Suarez-Cuervo, A.N. & León-Prieto, C. Incidence of injuries per 1000 hours of refereeing or training in soccer referees: A mini-review.
1004	Not relevant	Romand, P. & Pantaléon, N. A Qualitative Study of Rugby Coaches' Opinions About the Display of Moral Character. "Sport Psychol,[2020#5Year]IF->1.515#2.080" 21, 58-77 (2007).
1005	Not relevant	Romano, V., et al. Correlation between official and common field-based fitness tests in elite soccer referees.

S1 File. Studies Identification

1006	Not relevant	Romano, V., et al. Correlation between Official and Common Field-Based Fitness Tests in Elite Soccer Referees. Journal of Functional Morphology & Kinesiology 6, 1-9 (2021).
1007	Not relevant	Romano, V; Tuzi, M; Di Gregorio, A; Sacco, AM; Belviso, I; Sirico, F; Palermi, S; Nurzynska, D; Di Meglio, F; Castaldo, C; Pizzi, A; Montagnani, S
1008	Not relevant	Romero-García, G., Ortega-Toro, E. & Alarcón-López, F. FUNCIONES EJECUTIVAS Y AGENTES DEPORTIVOS. Journal of Sport & Health Research 15, 657-682 (2023).
1009	Not relevant	Rowsell, G., Coutts, A., Reaburn, P. & Hill-Haas, S. Effects of cold-water immersion on physical performance between successive matches in high-performance junior male soccer players. "J Sports Sci,[2020#5Year]IF->2.597#3.060" 27, 565-573 (2009).
1010	Not relevant	Royce, R. Game-players and game-playing: a response to kreider. "J Phil Sport,[2020#5Year]IF->0.867#0.814" 40, 225-239 (2013).
1011	Not relevant	Royce, R. Refereeing and Technology – Reflections on Collins' Proposals. "J Phil Sport,[2020#5Year]IF->0.867#0.814" 39, 53-64 (2012).
1012	Not relevant	Ruddock, A.D., et al. Combined active and passive heat exposure induced heat acclimation in a soccer referee before 2014 FIFA World Cup. "Springerplus,IF-#N/A" 5, 617 (2016).
1013	Not relevant	Ruddock, AD; Thompson, SW; Hudson, SA; James, CA; Gibson, OR; Mee, JA
1014	Not relevant	Ruiz, A.J., Albaladejo-García, C., Reina, R. & Moreno, F.J. Basketball referee's gaze behavior and stimulus selection in relation to visual angle perspective and officiating mechanics and expertise. European Journal of Human Movement 50, 4-18 (2023).
1015	Not relevant	Ruiz-Del-Solar, J., Arenas, M., Verschae, R. & Loncomilla, P. Visual detection of legged robots and its application to robot soccer playing and refereeing.

S1 File. Studies Identification

1016	Not relevant	Rundio, A. & Buning, R.J. Collegiate Sport Club Service Delivery: Moving Between Motivations and Constraints to Beneficial Outcomes. Journal of Park & Recreation Administration 40, 2-18 (2022).
1017	Not relevant	Sabag, E., Lidor, R., Arnon, M., Morgulev, E. & Bar-Eli, M. Teamwork and Decision Making among Basketball Referees: The 3PO Principle, Refereeing Level, and Experience. "Journal Of Human Kinetics,[2020#5Year]IF->1.664#1.886" 89, 1-22 (2023).
1018	Not relevant	Sadr, M.M., Saheb, T. & Farahani, A. A Mapping and Visualization of the Role of Artificial Intelligence in Sport Industry. Journal of Research in Sport Management & Marketing 5, 44-56 (2024).
1019	Not relevant	Sage, L. & Kavussanu, M. Multiple Goal Orientations as Predictors of Moral Behavior in Youth Soccer. "Sport Psychol,[2020#5Year]IF->1.515#2.080" 21, 417-437 (2007).
1020	Not relevant	Şahbaz, S., Kabadayı, M., Yılmaz, A.K. & Bostancı, Ö. EFFECT OF AN EIGHT-WEEK CORE STRENGTH TRAINING ON SOME PERFORMANCE PARAMETERS IN FOOTBALL REFEREES. Kinesiologia Slovenica 27, 155-167 (2021).
1021	Not relevant	Saldaris, J.M., Landers, G.J. & Lay, B.S. Physical and perceptual cooling: Improving cognitive function, mood disturbance and time to fatigue in the heat. Scandinavian Journal of Medicine & Science in Sports 30, 801-811 (2020).
1022	Not relevant	Salmon, D.M., et al. What they know and who they are telling: Concussion knowledge and disclosure behaviour in New Zealand adolescent rugby union players. "J Sports Sci,[2020#5Year]IF->2.597#3.060" 38, 1585-1594 (2020).
1023	Not relevant	Sam, M.P., Andrew, J.C. & Gee, S. The modernisation of umpire development: Netball New Zealand’s reforms and impacts. "European Sport Management Quarterly,[2020#5Year]IF->1.889#2.436" 18, 263-286 (2018).
1024	Not relevant	Samarein, MR; Samanipour, MH; Asjodi, F; Shokati, P; Fallahi, Z; Brownlee, TE; Brito, JP; Bragazzi, NL; Oliveira, R
1025	Not relevant	Samuel, R.D. Soccer Referees’ Transition to the Premier League: A Case Study Reflecting Individual Experiences and Consultancy. Case Studies in Sport & Exercise Psychology 3, 1-10 (2019).

S1 File. Studies Identification

1026	Not relevant	Samuel, R.D. Training prospective soccer referees using a deliberate practice perspective: The Israeli Excellence Program. Journal of Sport Psychology in Action 8, 184-196 (2017).
1027	Not relevant	Samuel, R.D., et al. The effects of the 2020–2021 Coronavirus pandemic change-event on football refereeing: evidence from the Israeli and Portuguese leagues. International Journal of Sport & Exercise Psychology 21, 33-55 (2023).
1028	Not relevant	Samuel, R.D., Galily, Y. & Tenenbaum, G. Who are you, ref? Defining the soccer referee's career using a change-based perspective. International Journal of Sport & Exercise Psychology 15, 118-130 (2017).
1029	Not relevant	Samuel, R.D., Tenenbaum, G. & Galily, Y. An integrated conceptual framework of decision-making in soccer refereeing. International Journal of Sport & Exercise Psychology 19, 738-760 (2021).
1030	Selected	Sánchez et al. 2022Association between Fitness Level and Physical Match Demands of Professional Female Football Referees
1031	Not relevant	Sánchez, M., Hernández, D., Carretero, M. & Sánchez-Sánchez, J. Level of Opposition on Physical Performance and Technical-Tactical Behaviour of Young Football Players. Apunts: Educació Física i Esports, 71-84 (2019).
1032	Not relevant	Sánchez, M., Hernández, D., Carretero, M. & Sánchez-Sánchez, J. Level of Opposition on Physical Performance and Technical-Tactical Behaviour of Young Football Players. Apunts: Educación Física y Deportes, 71-84 (2019).
1033	Not relevant	Sánchez, M., Sánchez-Sánchez, J., Bosque, M.V.D., Solano-Suárez, D. & Castillo, Y.D. Analysis of the distance covered according to absolute and relative thresholds in eleven-a-side and seven-a-side soccer referees during official matches.
1034	Not relevant	Sánchez, M., Sánchez-Sánchez, J., Villa del Bosque, M., Solano-Suárez, D. & Castillo, D. Análisis de la distancia recorrida atendiendo a umbrales absolutos y relativos en árbitros de fútbol 11 y fútbol 7 durante partidos oficiales. RICYDE. Revista Internacional de Ciencias del Deporte 16, 358-368 (2020).
1035	Not relevant	Sánchez, M.L.M., et al. Association between Fitness Level and Physical Match Demands of Professional Female Football Referees.

S1 File. Studies Identification

1036	Not English	Sánchez, M; Sánchez-Sánchez, J; del Bosque, MV; Solano-Suárez, D; Castillo, D.Analysis of the distance covered according to absolute and relative thresholds in eleven-a-side and seven-a-side soccer referees during official matches
1037	Not relevant	Sánchez-García, M., Sánchez-Sánchez, J., Rodríguez-Fernández, A., Solano, D. & Castillo, D. Relationships between Sprint Ability and Endurance Capacity in Soccer Referees. Sports (Basel) 6(2018).
1038	Not relevant	Sánchez-García, R. The spectacularization of violence in contemporary US bare-knuckle fighting. "Sport in Society,[2020#5Year]IF->0.939#Not Available" 23, 1645-1658 (2020).
1039	Not relevant	Sánchez-Oliver, AJ; Moreno-Pérez, V; Terrón-Manrique, P; Fernández-Ruiz, V; Quintana-Milla, I; Sánchez-Sánchez, J; Rodríguez, G; Ramos-Alvarez, JJ; Domínguez, R; López-Samanes, A
1040	Not relevant	Sánchez-Pay, A., Torres-Luque, G., Sanz-Rivas, D. & Courel-Ibáñez, J. The use of bounce in professional wheelchair tennis. International Journal of Sports Science & Coaching 15, 375-381 (2020).
1041	Not relevant	Santos, F., Strachan, L., Gould, D., Pereira, P. & Machado, C. The Role of Team Captains in Integrating Positive Teammate Psychological Development in High-Performance Sport. "Sport Psychol,[2020#5Year]IF->1.515#2.080" 33, 1-11 (2019).
1042	Not relevant	Santos, P., Miguel Silva, P. & Lago-Peñas, C. The ball recovery as an action related performance indicator in Football - an example using distinct operational definitions. Journal of Human Sport & Exercise 12, 96-105 (2017).
1043	Not relevant	Santos-Fernandez, E., Wu, P. & Mengersen, K.L. Bayesian statistics meets sports: a comprehensive review. Journal of Quantitative Analysis in Sports 15, 289-312 (2019).
1044	Not relevant	Santos-Silva, P.R., et al. Comparing the Aerobic Fitness of Professional Male Soccer Players and Soccer Referees.
1045	Not relevant	Sapp, R.M., Spangenburg, E.E. & Hagberg, J.M. Markers of aggressive play are similar among the top four divisions of English soccer over 17 seasons. Science & Medicine in Football 3, 125-130 (2019).

S1 File. Studies Identification

1046	Not relevant	Schaeperkoetter, C.C. Basketball officiating as a gendered arena: An autoethnography. "Sport Management Review,[2020#5Year]IF->3.337#3.761" 20, 128-141 (2017).
1047	Not relevant	Schaillée, H., et al. Gender inequality in sport: perceptions and experiences of generation Z. Sport, Education & Society 26, 1011-1025 (2021).
1048	Not relevant	Schalles, W. Rules. Amateur Wrestling News 68, 21-21 (2023).
1049	Review	Schenk, K., Bizzini, M. & Gatterer, H. Exercise physiology and nutritional perspectives of elite soccer refereeing. Scandinavian Journal of Medicine & Science in Sports 28, 782-793 (2018).
1050	Review	Schenk, K; Bizzini, M; Gatterer, H.Exercise physiology and nutritional perspectives of elite soccer refereeing
1051	Not relevant	Schiavon, M., Albertin, G., Stocco, E. & Rossi, M. Measurement of respiratory muscle endurance in soccer referees.
1052	Not relevant	Schlösser, L., et al. Validity of body fat percentage through different methods of body composition assessment in elite soccer referees. Brazilian Journal of Kineanthropometry & Human Performance 24, 1-12 (2022).
1053	Not relevant	Schmidt, S.L., et al. Decrease in Attentional Performance After Repeated Bouts of High Intensity Exercise in Association-Football Referees and Assistant Referees. "Front Psychol,[2020#5Year]IF->2.067#2.723" 10, 2014 (2019).
1054	Not relevant	Schmidt, SL; Schmidt, GJ; Padilla, CS; Simoes, EN; Tolentino, JC; Barroso, PR; Narciso, JH; Godoy, ES; Costa, RL
1055	Not relevant	Schoeman, R. & Schall, R. ANALYSIS OF POWER PLAY IN 2018 VARSITY CUP RUGBY COMPETITION. South African Journal for Research in Sport, Physical Education & Recreation 42, 133-146 (2020).
1056	Not relevant	Scholten, H. You've Got Three Choices: Give in, Give up, or Give it All You've Got: Does Contest Heterogeneity Affect Effort in Individual Competitions? "Journal Of Sports Economics,[2020#5Year]IF->1.615#1.527" 24, 932-965 (2023).

S1 File. Studies Identification

1057	Not relevant	Schütz, L.-M., Schweizer, G. & Plessner, H. The Impact of Video Speed on the Estimation of Time Duration in Sport. Journal of Sport & Exercise Psychology 43, 419-429 (2021).
1058	Not relevant	Schwarz, E., et al. Practitioner, Coach, and Athlete Perceptions of Evidence-Based Practice in Professional Sport in Australia. International Journal of Sports Physiology & Performance 16, 1728-1735 (2021).
1059	Not relevant	Schyvinck, C., Babiak, K., Constandt, B. & Willem, A. What Does Entrepreneurship Add to the Understanding of Corporate Social Responsibility Management in Sport? "J Sport Manage,[2020#5Year]IF->2.359#2.877" 35, 452-464 (2021).
1060	Not relevant	Scoppa, V. Fatigue and Team Performance in Soccer: Evidence From the FIFA World Cup and the UEFA European Championship. "Journal Of Sports Economics,[2020#5Year]IF->1.615#1.527" 16, 482-507 (2015).
1061	Not relevant	Scoz, R.D., et al. Diagnostic Validity of an Isokinetic Testing to Identify Partial Anterior Cruciate Ligament Injuries. "J Sport Rehab,[2020#5Year]IF->1.650#1.905" 29, 1086-1092 (2020).
1062	Not relevant	Segado Segado, F., Antonio Sánchez-Sáez, J., Maciá Andreu, M.J., Sánchez-Sánchez, J. & Gallardo Guerrero, A.M. Diseño y validación de un cuestionario para conocer las principales barreras y beneficios percibidos en el arbitraje femenino en el fútbol. Retos: Nuevas Perspectivas de Educación Física, Deporte y Recreación 43, 452-462 (2022).
1063	Not relevant	Seifried, C. Recognizing and Combating Emotive Language: Examples Associated With Sport. Quest (00336297) 60, 200-213 (2008).
1064	Not relevant	Seifried, C. Sport Facilities as a Broadcast Studio for Human Extensibility? Geographic Information System-Based Diagrams of a High- and Low-Identified Sport Fan. "J Sport Manage,[2020#5Year]IF->2.359#2.877" 25, 515-530 (2011).
1065	Not relevant	Seippel, Ø. Professionalization of voluntary sport organizations – a study of the Quality Club Programme of the Norwegian Football Association. "European Sport Management Quarterly,[2020#5Year]IF->1.889#2.436" 19, 666-683 (2019).

S1 File. Studies Identification

1066	Not relevant	Senécal, I., Howarth, S.J., Wells, G.D., Raymond, I. & Mior, S. The Impact of Moderate and High Intensity Cardiovascular Exertion on Sub-Elite Soccer Referee's Cognitive Performance: A Lab-Based Study. Journal of Sports Science & Medicine 20, 618-625 (2021).
1067	Not physical or physiological variables	Sergio L. Schmidt.Decrease in Attentional Performance After Repeated Bouts of High Intensity Exercise in Association-Football Referees and Assistant Referees
1068	Not relevant	Serpell, B.G., Colomer, C.M., Pickering, M.R. & Cook, C.J. Team Behavior and Performance: An Exploration in the Context of Professional Rugby Union. International Journal of Sports Physiology & Performance 18, 996-1003 (2023).
1069	Not relevant	Serrano, C; Sánchez-Sánchez, J; Felipe, JL; Hernando, E; Gallardo, L; Garcia-Unanue, J
1070	Not relevant	Serrano-Durá, J., Devís-Devís, J., Martínez-Baena, A. & Molina, P. Misconduct of Spanish grassroots soccer coaches through referees' reports: Variability by match format, age category, sex, and severity. International Journal of Sports Science & Coaching 19, 1916-1926 (2024).
1071	Not relevant	Serra-Olivares, J., García López, L.M. & Gonçalves, B. Effects of the players' level and age group category on positional tactical behaviour during 7- and 8-a-side football youth games. "International Journal Of Performance Analysis In Sport,[2020#5Year]IF->1.518#1.707" 19, 236-247 (2019).
1072	Not relevant	Sever, O., et al. How does the increase in foreign players affect football? BMC Sports Science, Medicine & Rehabilitation 15, 1-9 (2023).
1073	Not physical or physiological variables	Seydi et al.,Evaluation of the factors that affect performances of active football referees in Turkey

S1 File. Studies Identification

1074	Not relevant	Shapiro, D., Pate, J.R. & Cottingham, M. A Multi-Institutional Review of College Campus Adapted Intramural Sports Programming for College Students With and Without a Disability. Recreational Sports Journal 44, 109-125 (2020).
1075	Not relevant	Shavit, U. Being a Muslim football player in Europe. Soccer & Society 20, 271-287 (2019).
1076	Not relevant	Silva, H., Nakamura, F.Y., Beato, M. & Marcelino, R. Acceleration and deceleration demands during training sessions in football: a systematic review. Science & Medicine in Football 7, 198-213 (2023).
1077	Not relevant	Silva, J.R., Rumpf, M., Hertzog, M. & Nassis, G. Does the FIFA World Cup's Congested Fixture Program Affect Players' Performance? Asian Journal of Sports Medicine 8, 1-7 (2017).
1078	Not relevant	Silva, LLE; Neves, E; Silva, J; Alonso, L; Vale, R; Nunes, R.The haemodynamic demand and the attributes related to the displacement of the soccer referees in the moments of decision / intervention during the matches
1079	Not relevant	Silva, M.L., Oliveira, J.F.H. & Sampaio, A.J.E. Variation of decision-making of soccer referees based on experience.
1080	Conference Paper	Simoes, M; Visser, U; Buche, C; Rossi, A.Hybrid Methods for Real-Time Video Sequence Identification of Human Soccer Referee Signals
1081	Not relevant	Simon, R. Deserving to Be Lucky: Reflections on the Role of Luck and Desert in Sports. "J Phil Sport,[2020#5Year]IF->0.867#0.814" 34, 13-25 (2007).
1082	Not relevant	Simon, R.L. Does Athletics Undermine Academics? Examining Some Issues. Journal of Intercollegiate Sport 1, 40-58 (2008).
1083	Not relevant	Sinelnikov, O.A. & Hastie, P.A. Students' Autobiographical Memory of Participation in Multiple Sport Education Seasons. "J Teach Phys Educ,[2020#5Year]IF->1.845#2.490" 29, 167-183 (2010).
1084	Not relevant	Sirotic, A., Coutts, A., Knowles, H. & Catterick, C. A comparison of match demands between elite and semi-elite rugby league competition. "J Sports Sci,[2020#5Year]IF->2.597#3.060" 27, 203-211 (2009).

S1 File. Studies Identification

1085	Not relevant	SİVrİ, S. Examining the Self-Efficacy Levels of Tennis Officials Soner SİVRİ. Turkish Journal of Sport & Exercise / Türk Spor ve Egzersiz Dergisi 25, 369-378 (2023).
1086	Not comparisons in the time phase	Skidmore, N.THE PHYSIOLOGICAL DEMANDS OF PROFESSIONAL SOCCER REFEREEING ACROSS A SEASON
1087	Not relevant	Skopek, M., Heidler, J., Hnizdil, J.A.N., Kresta, J.A.N. & Vysocka, K. The use of virtual reality in table tennis training: a comparison of selected muscle activation in upper limbs during strokes in virtual reality and normal environments. Journal of Physical Education & Sport 23, 1736-1741 (2023).
1088	Not relevant	Slack, L.A., Maynard, I.W., Butt, J. & Olusoga, P. An Evaluation of a Mental Toughness Education and Training Program for Early-Career English Football League Referees. "Sport Psychol,[2020#5Year]IF->1.515#2.080" 29, 237-257 (2015).
1089	Not relevant	Sneyimani, T., Mathenjwa, M., Millard, L. & Breukelman, G.J. A Review of the Essential Visual Skills Required for Field Hockey: Beyond 20-20 Optometry. Asian Journal of Sports Medicine 14, 1-9 (2023).
1090	Not relevant	Snyder, K. & Lopez, M. Consistency, accuracy, and fairness: a study of discretionary penalties in the NFL. Journal of Quantitative Analysis in Sports 11, 219-230 (2015).
1091	Not relevant	Soebbing, B.P., Wicker, P., Weimar, D. & Orlowski, J. How do Bookmakers Interpret Running Performance of Teams in Previous Games? Evidence From the Football Bundesliga. "Journal Of Sports Economics,[2020#5Year]IF->1.615#1.527" 22, 231-250 (2021).
1092	Conference Paper	Solomon, A.V., Paik, C., Alhauli, A. & Phan, T. A decision support system for the professional soccer referee in time-sensitive operations.
1093	Not relevant	Solstad, B.E., Ivarsson, A., Haug, E.M. & Ommundsen, Y. Youth Sport Coaches' Well-Being Across the Season: The Psychological Costs and Benefits of Giving Empowering and Disempowering Sports Coaching to Athletes. International Sport Coaching Journal 5, 124-135 (2018).

S1 File. Studies Identification

1094	Not relevant	Soroka, A. & Bergier, J. THE RELATIONSHIP AMONG THE SOMATIC CHARACTERISTICS, AGE AND COVERED DISTANCE OF FOOTBALL PLAYERS. Human Movement 12, 353-360 (2011).
1095	Not relevant	Sotiriadou, K., Shilbury, D. & Quick, S. The Attraction, Retention/Transition, and Nurturing Process of Sport Development: Some Australian Evidence. "J Sport Manage,[2020#5Year]IF->2.359#2.877" 22, 247-272 (2008).
1096	Not relevant	Spitz, J., Moors, P., Wagemans, J. & Helsen, W.F. The impact of video speed on the decision-making process of sports officials. Cogn Res Princ Implic 3, 16 (2018).
1097	Not relevant	Spitz, J., Put, K., Wagemans, J., Williams, A.M. & Helsen, W.F. Does slow motion impact on the perception of foul play in football? "Eur J Sport Sci,[2020#5Year]IF->2.781#3.228" 17, 748-756 (2017).
1098	Not relevant	Spitz, J., Wagemans, J., Memmert, D., Williams, A.M. & Helsen, W.F. Video assistant referees (VAR): The impact of technology on decision making in association football referees. "J Sports Sci,[2020#5Year]IF->2.597#3.060" 39, 147-153 (2021).
1099	Not relevant	Spitz, J; Moors, P; Wagemans, J; Nelsen, WF.The impact of video speed on the decision-making process of sports officials
1100	Not relevant	Stahl, R.A. & Eckenrode, B.J. THE NONOPERATIVE REHABILITATION OF A TRAUMATIC COMPLETE ULNAR COLLATERAL LIGAMENT TEAR OF THE ELBOW IN A HIGH SCHOOL WRESTLER: A CASE REPORT. International Journal of Sports Physical Therapy 15, 1211-1221 (2020).
1101	Not relevant	Stefanou, L., Tsangaridou, N., Charalambous, C.Y. & Kyriakides, L. Examining the Contribution of a Professional Development Program to Elementary Classroom Teachers' Content Knowledge and Student Achievement: The Case of Basketball. "J Teach Phys Educ,[2020#5Year]IF->1.845#2.490" 40, 577-588 (2021).
1102	Not relevant	Steinfeldt, H., Dallmeyer, S. & Breuer, C. The Silence of the Fans: The Impact of Restricted Crowds on the Margin of Victory in the NBA. "International Journal Of Sport Finance,[2020#5Year]IF->0.550#1.050" 17, 165-177 (2022).
1103	Review	Stølen, T., Chamari, K., Castagna, C. & Wisløff, U. Physiology of soccer: An update.

1104	Not relevant	Stoney, E. & Fletcher, T. "Are Fans in the Stands an Afterthought?": Sports Events, Decision-Aid Technologies, and the Television Match Official in Rugby Union. Communication & Sport 9, 1008-1029 (2021).
1105	Not relevant	Sturm, D. From idyllic past-time to spectacle of accelerated intensity: televisual technologies in contemporary cricket. "Sport in Society,[2020#5Year]IF->0.939#Not Available" 24, 1305-1321 (2021).
1106	Not relevant	Štyriak, R., Hadža, R., Arriaza, R., Augustovičová, D. & Zemková, E. Effectiveness of Protective Measures and Rules in Reducing the Incidence of Injuries in Combat Sports: A Scoping Review. Journal of Functional Morphology & Kinesiology 8, 150 (2023).
1107	Not relevant	Suarez-Arrones, L., Calvo-Lluch, Á., Portillo, J., Sánchez, F. & Mendez-Villanueva, A. Running demands and heart rate response in rugby sevens referees. "J Strength Cond Res,[2020#5Year]IF->2.973#3.058" 27, 1618-1622 (2013).
1108	Not relevant	Sunderland, C., Taylor, E., Pearce, E. & Spice, C. Activity profile and physical demands of male field hockey umpires in international matches. "Eur J Sport Sci,[2020#5Year]IF->2.781#3.228" 11, 411-417 (2011).
1109	Not relevant	Sydney, M.G., Wollin, M., Chapman, D., Ball, N. & Mara, J.K. Substitute running outputs in elite youth male soccer players: less peak but greater relative running outputs. "Biol Sport,[2020#5Year]IF->2.000#2.250" 40, 241-248 (2023).
1110	Not relevant	Sydney, M.G., Wollin, M., Chapman, D.W., Ball, N. & Mara, J.K. Do conditioning focused various-sided training games prepare elite youth male soccer players for the demands of competition? "Biol Sport,[2020#5Year]IF->2.000#2.250" 39, 825-832 (2022).
1111	Not relevant	Szulc, A.M. THE REPORT AND ANALYSIS OF THE 2ND U21 EUROPEAN DEAF FOOTBALL CHAMPIONSHIP, STOCKHOLM, SWEDEN 2018. Human Movement 20, 80-87 (2019).
1112	Not relevant	Szwarc, A., et al. Motion analysis of elite Polish soccer goalkeepers throughout a season. "Biol Sport,[2020#5Year]IF->2.000#2.250" 36, 357-363 (2019).

1113	Not relevant	Szymski, D., et al. High injury rates and weak injury prevention strategies in football referees at all levels of play. Scandinavian Journal of Medicine & Science in Sports 32, 391-401 (2022).
1114	Not comparisons in the time phase	T. Asami, H. Togari, J. Ohashi.Analysis of movement patterns of referees during soccer matches.
1115	Selected	T.Asami,H.Togari & J.Ohashi, 1988/Asami, Togari & Ohashi.1988Analysis of Movement Pattern of Referees during soccer matches
1116	Not relevant	Tabassum, Y., Butt, M.Z.I. & Roohi, N. EFFECT OF PLAYING VENUE ON UNIVERSITY MALE VOLLEYBALL PLAYERS' PERCEPTION OF EFFORT. Shield: Research Journal of Physical Education & Sports Science 16, 102-116 (2021).
1117	Not relevant	Tabuk, M.E. Futbolda Uygulanan Video Yardımcı Hakem Sistemi Hakkında Taraftar Tutumlarını Belirlemeye Yönelik Ölçek Geliştirme Çalışması. CBÜ Beden Eğitimi & Spor Bilimleri Dergisi 17, 432-449 (2022).
1118	Not relevant	Tan, T.-C. & Lee, J.W. Technology, innovation, and the future of the sport industry in Asia Pacific. "Sport in Society,[2020#5Year]IF->0.939#Not Available" 26, 383-389 (2023).
1119	Not relevant	Taylor, L., et al. Exposure to hot and cold environmental conditions does not affect the decision making ability of soccer referees following an intermittent sprint protocol. "Front Physiol,[2020#5Year]IF->3.367#3.697" 5, 185 (2014).
1120	Not relevant	Taylor, L; Fitch, N; Castle, P; Watkins, S; Aldous, J; Sculthorpe, N; Midgely, A; Brewer, J; Mauger, A
1121	Not relevant	Tena, J.D. & Tovar, J. Emotional Shocks and Performance: Evidence from the FIFA World Cup. "International Journal Of Sport Finance,[2020#5Year]IF->0.550#1.050" 18, 150-165 (2023).
1122	Not relevant	Teoldo da Costa, I. & de Freitas Silvino, M.P. Analysis of tactical behavior in full- and small-sided games: Comparing professional and youth academy athletes to enhance player development in soccer. International Journal of Sports Science & Coaching 18, 132-142 (2023).

1123	Not relevant	Teques, P., et al. Mediating Effects of Parents' Coping Strategies on the Relationship Between Parents' Emotional Intelligence and Sideline Verbal Behaviors in Youth Soccer. Journal of Sport & Exercise Psychology 40, 153-162 (2018).
1124	Not relevant	Tessitore, A., Cortis, C., Meeusen, R. & Capranica, L. Power performance of soccer referees before, during, and after official matches.
1125	Not relevant	Unkelbach, C. & Memmert, D. Crowd Noise as a Cue in Referee Decisions Contributes to the Home Advantage. Journal of Sport & Exercise Psychology 32, 483-498 (2010).
1126	Not relevant	Urhausen, A., Vivas, J.P., Lambert, C. & Weiler, B. Cardiovascular Stress in Football Referees.
1127	Not relevant	Vachon, A., Berryman, N., Mujika, I., Paquet, J.-B. & Bosquet, L. Preconditioning Activities to Enhance Repeated High-Intensity Efforts in Elite Rugby Union Players. International Journal of Sports Physiology & Performance 17, 871-878 (2022).
1128	Not relevant	Vahed, Y., Kraak, W. & Venter, R. Changes on the match profile of the South African Currie Cup tournament during 2007 and 2013. International Journal of Sports Science & Coaching 11, 85-97 (2016).
1129	Not relevant	Valencia-Aguirre, O.H., Bravo-Navarro, W.H., Loaiza-Dávila, L.E. & Valencia-Cárdenas, M.H. Incidence of tactical formations on the results of soccer matches played at altitude. Retos: Nuevas Perspectivas de Educación Física, Deporte y Recreación 50, 408-414 (2023).
1130	Not relevant	Van Someren, K.A., et al. PART IV: PHYSIOLOGY. "J Sports Sci,[2020#5Year]IF->2.597#3.060" 19, 32-68 (2001).
1131	Not relevant	Vanessa Wergin, V., Zimanyi, Z. & Beckmann, J. A field study investigating running distance and affect of field hockey players in collective team collapse situations. International Journal of Sport & Exercise Psychology 19, 584-597 (2021).

S1 File. Studies Identification

1132	Not relevant	Vansteenkiste, M., Mouratidis, A. & Lens, W. Detaching Reasons From Aims: Fair Play and Well-Being in Soccer as a Function of Pursuing Performance-Approach Goals for Autonomous or Controlling Reasons. Journal of Sport & Exercise Psychology 32, 217-242 (2010).
1133	Not English	Vargas, GEF; da Silva, AI; Arruda, M.Anthropometric Profile and Physical Fitness of the Professional Referees Chilean Soccer
1134	Not relevant	Vasilica, I., Silva, R., Costa, P., Figueira, B. & Vaz, L. What is the Motivation to study Laws of the Game and Competition Rules in National Portuguese Football Referees? Sport Mont 18, 17-24 (2020).
1135	Not relevant	Vasilica, I., Silva, R.U.I., Costa, P., Figueira, B. & Vaz, L. Football refereeing: a systematic review and literature mapping. Journal of Physical Education & Sport 22, 388-401 (2022).
1136	Not relevant	Vella, A., et al. Possession chain factors influence movement demands in elite Australian football match-play. Science & Medicine in Football 5, 72-78 (2021).
1137	Not relevant	Vella, A., et al. Technical involvements and pressure applied influence movement demands in elite Australian Football Match-play. Science & Medicine in Football 6, 228-233 (2022).
1138	Not relevant	Venables, M. SportsTech.
1139	Not relevant	Vincent, G.E., et al. The Impact of Self-Reported Sleep Quantity on Perceived Decision-Making in Sports Officials During a Competitive Season. Research Quarterly for Exercise & Sport 92, 156-169 (2021).
1140	Not relevant	Voigt, L., et al. Advancing judgment and decision-making research in sport psychology by using the body as an informant in embodied choices. Asian Journal of Sport & Exercise Psychology 3, 47-56 (2023).
1141	Not relevant	Vollavanh, L.R., et al. Effect of Impact Mechanism on Head Accelerations in Men's Lacrosse Athletes. "J Appl Biomech,[2020#5Year]IF->1.617#1.655" 34, 396-402 (2018).
1142	Review	Volpi, P. Epidemiology and risk factors in soccer.

S1 File. Studies Identification

1143	Not relevant	Vors, O., et al. A review of the penetration of Francophone research on intervention in physical education and sport in Anglophone journals since 2010. Physical Education & Sport Pedagogy 25, 331-345 (2020).
1144	Not relevant	Wagner, H., Finkenzeller, T., Würth, S. & von Duvillard, S.P. Individual and Team Performance in Team-Handball: A Review. Journal of Sports Science & Medicine 13, 808-816 (2014).
1145	Not relevant	Wahl-Alexander, Z., Malecki, A. & Smart, S. The Effect of Prompting and Group Contingency on Middle School Students' Physical Activity During a Badminton Sport Education Season. Physical Educator 77, 208-229 (2020).
1146	Not relevant	Wahl-Alexander, Z., Richards, K.A., Washburn, N. & Sinelnikov, O. Changes in Male Campers' Goal Orientations and Motivation in Traditional Activities and a Sport Education Season in a Summer Camp. Journal of Park & Recreation Administration 36, 141-159 (2018).
1147	Not relevant	Wang, J. Key Principles of Open Motor-skill Training for Peak Performance. JOPERD: The Journal of Physical Education, Recreation & Dance 87, 8-15 (2016).
1148	Not relevant	Wang, XF.The Evaluation of Professional Skills for the Students Majoring in Basketball Field of Physical Education in Colleges
1149	Not relevant	Wass, J., et al. A comparison of match demands using ball-in-play vs. whole match data in elite male youth soccer players. Science & Medicine in Football 4, 142-147 (2020).
1150	Not relevant	Watanabe, N., Wicker, P. & Yan, G. Weather Conditions, Travel Distance, Rest, and Running Performance: The 2014 FIFA World Cup and Implications for the Future. "J Sport Manage,[2020#5Year]IF->2.359#2.877" 31, 27-43 (2017).
1151	Not relevant	Webb, T., O'Gorman, J. & Markham, L. Striving for excellence: talent identification and development in English football refereeing. "European Sport Management Quarterly,[2020#5Year]IF->1.889#2.436" 23, 351-369 (2023).
1152	Not relevant	Weber, J., et al. Heading and risk of injury situations for the head in professional German football: a video analysis of over 150,000 headers in 110,000 match minutes. Science & Medicine in Football 7, 307-314 (2023).

S1 File. Studies Identification

1153	Not relevant	Wegmann, M., et al. Cardiovascular risk and fitness in veteran football players. "J Sports Sci,[2020#5Year]IF->2.597#3.060" 34, 576-583 (2016).
1154	Not relevant	Wendt, J.T. Tokyo 2020-The Games of Hope, Solidarity and Peace. Entertainment & Sports Lawyer 38, 24-41 (2022).
1155	Not relevant	Wesołowska, J.M., et al. Comparison of the risk of cardiovascular diseases, stroke, and diabetes among the selected group of football referees and the group of general population men from Northern Poland – a pilot study.
1156	Not relevant	West, S.W., et al. Same name, same game, but is it different? An investigation of female rugby union match events in Canadian Varsity players. International Journal of Sports Science & Coaching 17, 1119-1127 (2022).
1157	Not physical or physiological variables	Westbrooks J, Low DA, Brownlee TE.
1158	Selected	Weston et al.2007Analysis of physical match performance in English Premier League soccer referees with particular reference to first half and player work rates
1159	Not comparisons in the time phase	Weston M, Castagna C, Impellizzeri FM, Rampinini E, Abt G
1160	book	Weston, M. & Helsen, W. Match officials.
1161	Review	Weston, M. Match performances of soccer referees: The role of sports science.
1162	Not relevant	Weston, M., Bird, S., Helsen, W., Nevill, A. & Castagna, C. The effect of match standard and referee experience on the objective and subjective match workload of English Premier League referees. "J Sci Med Sport,[2020#5Year]IF->3.607#4.332" 9, 256-262 (2006).

S1 File. Studies Identification

1163	Not relevant	Weston, M., Castagna, C., Helsen, W. & Impellizzeri, F. Relationships among field-test measures and physical match performance in elite-standard soccer referees. "J Sports Sci,[2020#5Year]IF->2.597#3.060" 27, 1177-1184 (2009).
1164	Not relevant	Weston, M., Castagna, C., Impellizzeri, F.M., Rampinini, E. & Abt, G. Analysis of physical match performance in English Premier League soccer referees with particular reference to first half and player work rates. "J Sci Med Sport,[2020#5Year]IF->3.607#4.332" 10, 390-397 (2007).
1165	Not relevant	Weston, M., Drust, B., Atkinson, G. & Gregson, W. Variability of soccer referees' match performances.
1166	Not relevant	Weston, M., Drust, B., Atkinson, G. & Gregson, W. Variability of soccer referees' match performances. "Int J Sports Med,[2020#5Year]IF->2.556#2.616" 32, 190-194 (2011).
1167	Not relevant	Weston, M., et al. Changes in a Top-Level Soccer Referee's Training, Match Activities, and Physiology Over an 8-Year Period: A Case Study. International Journal of Sports Physiology & Performance 6, 281-286 (2011).
1168	Not relevant	Weston, M., et al. Reduction in physical match performance at the start of the second half in elite soccer.
1169	Not relevant	Weston, M., et al. Reduction in Physical Match Performance at the Start of the Second Half in Elite Soccer. International Journal of Sports Physiology & Performance 6, 174-182 (2011).
1170	Review	Weston, M., et al. Science and medicine applied to soccer refereeing: An update.
1171	Not relevant	Weston, M., et al. Science and medicine applied to soccer refereeing: an update. "Sports Med,[2020#5Year]IF->8.551#9.761" 42, 615-631 (2012).
1172	Not relevant	Weston, M., Helsen, W., MacMahon, C. & Kirkendall, D. The Impact of Specific High-Intensity Training Sessions on Football Referees' Fitness Levels.

S1 File. Studies Identification

1173	Not relevant	Weston, M; Batterham, AM; Castagna, C; Portas, MD; Barnes, C; Harley, J; Lovell, RJ
1174	Not relevant	Weston, M; Bird, S; Helsen, W; Nevill, A; Castagna, C.The effect of match standard and referee experience on the objective and subjective match workload of English Premier League referees
1175	Not relevant	Weston, M; Castagna, C; Helsen, W; Impellizzeri, F.Relationships among field-test measures and physical match performance in elite-standard soccer referees
1176	Not relevant	Weston, M; Castagna, C; Impellizzeri, FM; Bizzini, M; Williams, AM; Gregson, W
1177	Review	Weston, M; Castagna, C; Impellizzeri, FM; Bizzini, M; Williams, AM; Gregson, W.Science and Medicine Applied to Soccer Refereeing An Update
1178	Not relevant	Weston, M; Castagna, C; Impellizzeri, FM; Rampinini, E; Abt, G.Analysis of physical match performance in English Premier League soccer referees with particular reference to first half and player work rates
1179	Not relevant	Weston, M; Castagna, C; Impellizzeri, FM; Rampinini, E; Breivik, S.Ageing and physical match performance in English Premier League soccer referees
1180	Not relevant	Weston, M; Drust, B; Atkinson, G; Gregson, W.Variability of Soccer Referees' Match Performances
1181	Not relevant	Weston, M; Drust, B; Gregson, W.Intensities of exercise during match-play in FA Premier League referees and players
1182	Not relevant	Wibowo, T.A., Ndayisenga, J. & Susanto, R. Basketball Free Throw Skill Level of Grade 8 Boys of Pius Gombong Junior High School. JUMORA: Jurnal Moderasi Olahraga 3, 190-201 (2023).
1183	Not relevant	Wicker, P. & Downward, P. The Causal Effect of Voluntary Roles in Sport on Subjective Well-Being in European Countries. "J Sport Manage,[2020#5Year]IF->2.359#2.877" 34, 303-315 (2020).

S1 File. Studies Identification

1184	Not relevant	Wicker, P., Orlowski, J. & Weimar, D. Referees' Card-Awarding Behavior and Performance Evaluation in Professional Football: The Role of Teams' Running Distance and Speed.
1185	Not relevant	Wicker, P., Orlowski, J. & Weimar, D. Referees' Card-Awarding Behavior and Performance Evaluation in Professional Football: The Role of Teams' Running Distance and Speed. "International Journal Of Sport Finance, [2020#5Year]IF->0.550#1.050" 17, 62-72 (2022).
1186	Not relevant	Wicker, P; Orlowski, J; Weimar, D.Referees' Card-Awarding Behavior and Performance Evaluation in Professional Football: The Role of Teams' Running Distance and Speed
1187	Not relevant	Widiyanto, Wirya, D.T., Hariono, A., Ndayisenga, J. & Shahril, M.I.B. ASSESSMENT OF TAEKWONDO ATTENDANCE PREDOMINANT SYSTEM DURING COMPETITION ON NEW GAME REGULATIONS STUDY IN INDONESIAN TAEKWONDO. International Journal of Sports Sciences & Fitness 11, 28-43 (2021).
1188	Not relevant	Wigo, B. WATER POLO BEHIND THE VEIL. Swimming World 64, 43-46 (2023).
1189	Not relevant	Williams, J.H., Hoffman, S., Jaskowak, D.J. & Tegarden, D. Physical demands and physiological responses of extra time matches in collegiate women's soccer. Science & Medicine in Football 3, 307-312 (2019).
1190	Not relevant	Williams, R.L., Jeffriess, M.D., Black, C.R. & Minichiello, M.L. INCORPORATING POSTERIOR CHAIN STRENGTH EXERCISES TO REDUCE INJURY RATES OF NATIONAL RUGBY LEAGUE REFEREES. Journal of Australian Strength & Conditioning 25, 70-84 (2017).
1191	Not relevant	Wilson, C; Scanlan, M; Bradbury, DG; Barley.Intervention Accuracy and Perception of Fatigue in State-Level Australian Football Umpires
1192	Not relevant	Wilson, D. & Ramchandani, G. A Comparative Analysis of Home Advantage in the Olympic and Paralympic Games 1988–2018. Journal of Global Sport Management 6, 170-184 (2021).
1193	Not relevant	Wilson, D.C., Ruddock, A.D., Ranchordas, M.K., Thompson, S.W. & Rogerson, D. Physical profile of junior and senior amateur boxers. Journal of Physical Education & Sport 20, 3452-3459 (2020).

S1 File. Studies Identification

1194	Not relevant	Wilson, F., Byrne, A. & Gissane, C. Prospective study of injury and activity profile in elite soccer referees and assistant referees.
1195	Not relevant	Wing, C., Hart, N.H., Ma'ayah, F. & Nosaka, K. Physical and technical demands of offence, defence, and contested phases of play in Australian Football. BMC Sports Science, Medicine & Rehabilitation 14, 1-13 (2022).
1196	Not relevant	Wolfe, T., Wolfe, A., Bowers, S.T. & McEntyre, K. Using Sport Education to Deliver a Powerlifting University Physical Activity Course. JOPERD: The Journal of Physical Education, Recreation & Dance 92, 11-17 (2021).
1197	Not relevant	Wolfinger, C.R. & Davenport, T.E. PHYSICAL THERAPY MANAGEMENT OF ICE HOCKEY ATHLETES: FROM THE RINK TO THE CLINIC AND BACK. International Journal of Sports Physical Therapy 11, 482-495 (2016).
1198	Not relevant	Wolf-Root, A. On being part of a game. "J Phil Sport,[2020#5Year]IF->0.867#0.814" 47, 75-88 (2020).
1199	Not relevant	Wolter, S. A Critical Discourse Analysis of espnW: Divergent Dialogues and Postfeminist Conceptions of Female Fans and Female Athletes. International Journal of Sport Communication 8, 345-370 (2015).
1200	Not relevant	Woods, C.T., Robertson, S. & Collier, N.F. Evolution of game-play in the Australian Football League from 2001 to 2015. "J Sports Sci,[2020#5Year]IF->2.597#3.060" 35, 1879-1887 (2017).
1201	Not relevant	Woods, C.T., Veale, J., Fransen, J., Robertson, S. & Collier, N.F. Classification of playing position in elite junior Australian football using technical skill indicators. "J Sports Sci,[2020#5Year]IF->2.597#3.060" 36, 97-103 (2018).
1202	Not relevant	Woodward, K. Women’s time? Time and temporality in women’s football. "Sport in Society,[2020#5Year]IF->0.939#Not Available" 20, 689-700 (2017).
1203	Not relevant	Wühr, P., Fasold, F. & Memmert, D. Soccer offside judgments in laypersons with different types of static displays.
1204	Conference Paper	Xu, J., Zhang, Y., Ye, A. & Dai, F. Real-time detection of game handball foul based on target detection and skeleton extraction.

S1 File. Studies Identification

1205	Not relevant	Yaghoubi, M., Lark, S.D., Page, W.H., Fink, P.W. & Shultz, S.P. Lower extremity muscle function of front row rugby union scrummaging. "Sport Biomech,[2020#5Year]IF->2.023#1.949" 18, 636-648 (2019).
1206	Not relevant	Yan, G., Watanabe, N.M., Shapiro, S.L., Naraine, M.L. & Hull, K. Unfolding the Twitter scene of the 2017 UEFA Champions League Final: social media networks and power dynamics. "European Sport Management Quarterly,[2020#5Year]IF->1.889#2.436" 19, 419-436 (2019).
1207	Not relevant	Yanaoka, T., Yamagami, J., Kidokoro, T., Kashiwabara, K. & Miyashita, M. Halftime rewarm-up with intermittent exercise improves the subsequent exercise performance of soccer referees.
1208	Not relevant	Yanci, J., Los Arcos, A., Grande, I. & Casajús, J.A. Change of direction ability test differentiates higher level and lower level soccer referees. "Biol Sport,[2020#5Year]IF->2.000#2.250" 33, 173-177 (2016).
1209	Not relevant	Yanci, J., Los, A.A., Grande, I. & Casajús, J.A. Change of direction ability test differentiates higher level and lower level soccer referees. "Biol Sport,[2020#5Year]IF->2.000#2.250" 33, 173-177 (2016).
1210	Not relevant	Yantha, Z.D., McKay, B. & Ste-Marie, D.M. The recommendation for learners to be provided with control over their feedback schedule is questioned in a self-controlled learning paradigm. "J Sports Sci,[2020#5Year]IF->2.597#3.060" 40, 769-782 (2022).
1211	Conference Paper	Yetongnon, K; Dipanda, A; Chbeir, R.Event Detection and Recognition Using HMM with Whistle Sounds
1212	Not relevant	Yi, Q., et al. Technical and physical match performance of teams in the 2018 FIFA World Cup: Effects of two different playing styles. "J Sports Sci,[2020#5Year]IF->2.597#3.060" 37, 2569-2577 (2019).
1213	Not relevant	Yi, Q., Jia, H., Liu, H. & Gómez, M.Á. Technical demands of different playing positions in the UEFA Champions League. "International Journal Of Performance Analysis In Sport,[2020#5Year]IF->1.518#1.707" 18, 926-937 (2018).
1214	Not relevant	Yiannaki, C., Barron, D.J., Collins, D. & Carling, C. Match performance in a reference futsal team during an international tournament -- implications for talent development in soccer. "Biol Sport,[2020#5Year]IF->2.000#2.250" 37, 147-156 (2020).

S1 File. Studies Identification

1215	Not relevant	Yol, Y., Turgay, F., Yigittürk, O., Aşıkovalı, S. & Durmaz, B. The effects of regular aerobic exercise training on blood nitric oxide levels and oxidized LDL and the role of eNOS intron 4a/b polymorphism. Biochim Biophys Acta Mol Basis Dis 1866, 165913 (2020).
1216	Not relevant	Yoo, J.J., Choi, D. & Bang, H. Consumer Response to Patriotic Ads for Domestic Versus Foreign Brands in Contexts of International Sporting Events: The Role of National Identity Activation and Group Emotion. Communication & Sport 11, 905-928 (2023).
1217	Not relevant	Yoshimitsu, K. Importance of vision and sun protection in outdoor sports: a narrative review. Journal of Physical Education & Sport 21, 3187-3194 (2021).
1218	Not relevant	Young, C.M., Luo, W., Gastin, P., Lai, J. & Dwyer, D.B. Understanding effective tactics in Australian football using network analysis. "International Journal Of Performance Analysis In Sport,[2020#5Year]IF->1.518#1.707" 19, 331-341 (2019).
1219	Selected	Yousefian et al. 2022Intensity demands and peak performance of elite soccer referees during match play
1220	Not relevant	Yousefian, F., et al. Intensity demands and peak performance of elite soccer referees during match play.
1221	Not relevant	Yukhymenko-Lescroart, M.A. Defining and Measuring Character in Sport From a Multidimensional Perspective. International Sport Coaching Journal 6, 250-253 (2019).
1222	Not comparisons in the time phase	Z.Cheng.The physical training of Chinese football association Super league referees and assistant referees
1223	Not relevant	Zadarko, E., et al. Diagnostics of selected motor skills of Oyama Karate competitors preparing for the championships. Ido Movement for Culture. Journal of Martial Arts Anthropology 19, 102-106 (2019).
1224	Not relevant	Zadorozhna, O., et al. Indicators of athletes' effectiveness as a basis of team tactical training in women epee fencing. Trends in Sport Sciences 27, 191-202 (2020).

S1 File. Studies Identification

1225	Not relevant	Zago, M., et al. Kinematic effects of repeated turns while running. "Eur J Sport Sci,[2020#5Year]IF->2.781#3.228" 19, 1072-1081 (2019).
1226	Not relevant	Zerf, M., Hadje, B., Benaouda, B. & Beboucha, W. Observation method and its weaknesses in selecting Algerian goalkeepers. Journal of Physical Education & Sport 17, 1992-1998 (2017).
1227	Conference Paper	Zhang, T; Luo, Q.Kneejerk: Intuition, Cognition and Hindsight Bias in Team Games
1228	Conference Paper	Zhang, X., Cheng, H.M., Li, J.Y., Zhou, S.Q. & Yang, T.X. Numerical simulation of pressure difference about curve ball in flight.
1229	Not relevant	Zhang, Y. & Breedlove, J. Sustaining market competitiveness of table tennis in China through the application of digital technology. "Sport in Society, [2020#5Year]IF->0.939#Not Available" 24, 1770-1790 (2021).
1230	Not relevant	Zhao, Y. & Zhang, H. Investigating the inter-country variations in game interruptions across the Big-5 European football leagues. "International Journal Of Performance Analysis In Sport, [2020#5Year] IF->1.518#1.707" 21, 180-196 (2021).
1231	Not relevant	Zhao, Y. & Zhang, H. Sabotage in dynamic tournaments with heterogeneous contestants: Evidence from European football. International Journal of Sports Science & Coaching 18, 552-562 (2023).
1232	Not comparisons in the time phase	Zheming Ma.Research on China's Super league referee characteristics and activity energy expenditure
1233	Conference Paper	Zheng, GH.The Evaluation of Professional Skills for the Students Majoring in Basketball Field of Physical Education in Colleges
1234	Not relevant	Zheng, J., Tan, T.-C. & Jiang, R.-S. Chance events and strategic competitive advantage in elite sport: crises and sport-specific chance events. "Sport in Society,[2020#5Year]IF->0.939#Not Available" 27, 33-51 (2024).

S1 File. Studies Identification

1235	Not relevant	Zhou, C., Calvo, A.L., Robertson, S. & Gómez, M.-Á. Long-term influence of technical, physical performance indicators and situational variables on match outcome in male professional Chinese soccer. "J Sports Sci,[2020#5Year]IF->2.597#3.060" 39, 598-608 (2021).
1236	Not relevant	Zhou, C., Zhang, S., Lorenzo Calvo, A. & Cui, Y. Chinese soccer association super league, 2012-2017: key performance indicators in balance games. "International Journal Of Performance Analysis In Sport,[2020#5Year]IF->1.518#1.707" 18, 645-656 (2018).
1237	Not relevant	Ziaee, A., Adib-Moghaddam, A., Elling, A., van Sterkenburg, J. & van Hilvoorde, I. Football and the media construction of Iranian national identity during the FIFA World Cup 2018 and AFC Asian Cup 2019. Soccer & Society 22, 613-625 (2021).
1238	Not comparisons in the time phase	Zou Yong.physical energy load of National- class Football Referees in Judging and Countermeasures of Improving Physical Energy Level
1239	Conference Paper	Zvonar, M; Sajdlova, Z. COMPARISON OF PHYSICAL PREPAREDNESS OF FOOTBALL REFEREES OF DIFFERENT COMPETITION LEVELS
